# Next-generation hybrid bioanalytical platforms and clinical integration of TDM technologies for precision monitoring to optimize last-resort antibiotic therapy 

**DOI:** 10.3389/fphar.2026.1761582

**Published:** 2026-07-09

**Authors:** Aparna Inamdar, Narasimha M. Beeraka, P. R. Hemanth Vikram, Bannimath Gurupadayya, S. R. Sanathan, Akila Prashant, Tegginamath Pramod Kumar, Vladimir N. Nikolenko, Y. Padmanabha Reddy, Dilipkumar Reddy Kandula, Basappa Basappa

**Affiliations:** 1 Department of Pharmaceutical Chemistry, JSS College of Pharmacy, JSS Academy of Higher Education and Research (JSS AHER), Mysuru, Karnataka, India; 2 Department of Human Anatomy and Histology, I.M. Sechenov First Moscow State Medical University (Sechenov University), Moscow, Russia; 3 Saveetha Institute of Basic Medical Sciences (SIBMS), Saveetha Institute of Medical and Technical Sciences, Saveetha University, Chennai, Tamil Nadu, India; 4 School of Medical Science and Technology, Indian Institute of Technology Kharagpur, Kharagpur, West Bengal, India; 5 Department of Pharmacy Practice, JSS College of Pharmacy, JSS Academy of Higher Education and Research (JSS AHER), Mysuru, Karnataka, India; 6 Department of Biochemistry, JSS Medical College and Hospital, JSS-AHER, Karnataka, Mysuru, India; 7 Department of Pharmaceutics, JSS College of Pharmacy Mysuru, JSS Academy of Higher Education and Research (JSSAHER), Mysuru, India; 8 Department of Pharmacology, Raghavendra Institute of Pharmaceutical Education and Research (RIPER), Chiyyedu, Andhra Pradesh, India; 9 Department of Pharmacy, Shri JJT University, Jhunjhunu, Rajasthan, India; 10 Laboratory of Chemical Biology, Department of Studies in Organic Chemistry, University of Mysore, Mysore, Karnataka, India

**Keywords:** antibiotics, antimicrobial resistance (AMR), artificial intelligence, bayesian forecasting, bioanalytical-biosensor technologies, population pharmacokinetics, precision dosing, therapeutic drug monitoring (TDM)

## Abstract

**Background:**

The global rise of multidrug-resistant (MDR) infections has intensified the clinical reliance on last-resort antibiotics such as vancomycin, linezolid, and tigecycline, particularly in critically ill patients. These agents possess narrow therapeutic windows, complex pharmacokinetics, and substantial inter-patient variability, making therapeutic drug monitoring (TDM) a cornerstone of individualized therapy. Traditional TDM methodologies though accurate are often limited by centralized processing, slow turnaround times, and cost constraints, which hinder real-time clinical decision-making in intensive care settings.

**Objective:**

This review aims to critically evaluate emerging bioanalytical platforms, modeling frameworks, and decision-support systems for optimizing TDM of last-resort antibiotics. It focuses on enhancing precision dosing, improving clinical outcomes, and addressing antimicrobial resistance through integration of innovative sensor technologies and artificial intelligence.

**Methods:**

A comprehensive literature search was conducted across databases, including PubMed, Google Scholar, Scopus, and Nature. Relevant studies were analyzed for analytical techniques, matrix types, extraction strategies, assay validation parameters, and clinical applicability. Emphasis was placed on comparing high-performance liquid chromatography (HPLC), LC–MS/MS, and immunoassays with novel approaches such as microneedle-based biosensors, real-time urinary antibiotic monitoring, wearable devices, and luciferase-based bioluminescent sensors. Mechanism-based pharmacokinetic/pharmacodynamic (PK/PD) modelling and Bayesian forecasting frameworks were reviewed for their role in adaptive dosing.

**Results:**

LC–MS/MS emerged as the most sensitive and specific platform, while immunoassays provided practical solutions for near-patient testing. Innovations such as microsampling, temperature-responsive two-dimensional chromatography, and bioluminescent sensor platforms demonstrated potential to overcome the limitations of conventional assays. Integration of population pharmacokinetic (PPK) models and AI-driven decision-support algorithms enhanced predictive precision, allowing dynamic dose optimization for β-lactam antibiotics, tetracyclines, vancomycin, linezolid, and tigecycline. A three-tiered TDM model was proposed, combining site-specific sensing, real-time analysis, and computational forecasting to improve antimicrobial stewardship.

**Conclusion:**

Emerging bioanalytical technologies and predictive PK/PD modeling are transforming TDM from a static laboratory tool into a real-time, precision-guided clinical decision platform. The integration of minimally invasive sensing technologies with AI-enabled dose optimization offers a path toward personalized antibiotic therapy, optimized clinical outcomes, and the mitigation of antimicrobial resistance in critical care settings. This paradigm shift supports a more adaptive and responsive approach to TDM, ensuring last-resort antibiotics are used effectively and sustainably.

## Highlights

- Precision TDM integration: Emerging bioanalytical platforms enable rapid and sensitive detection of last-resort antibiotics in critically ill patients, overcoming the limitations of conventional centralized laboratory testing.
- Technological advancements: Innovative approaches such as luciferase-based bioluminescent sensors, microneedle devices, and temperature-responsive chromatography enhance point-of-care antibiotic monitoring.
- Predictive modeling: Population PK/PD models and AI-driven Bayesian algorithms support individualized dose adjustments and real-time therapeutic optimization.
- Clinical relevance: A three-tiered TDM model is proposed to integrate site-specific sensing, real-time quantification, and computational forecasting, strengthening antimicrobial stewardship.
- Strategic impact: These advancements have the potential to improve treatment outcomes, reduce therapeutic failures, and mitigate the emergence of resistant bacterial strains.
- Novelty statement: This review introduces an integrative framework that merges next-generation sensing technologies with advanced PK/PD modeling and AI-based decision-support systems to modernize TDM of last-resort antibiotics. Unlike traditional static monitoring approaches, this model emphasizes real-time, minimally invasive, and site-specific drug quantification, coupled with predictive dosing strategies. Such an approach represents a paradigm shift from empirical dosing toward dynamic, precision-guided antimicrobial therapy, directly addressing the clinical and global challenge of AMR.

## Introduction

The increasing global crisis of AMR poses a profound threat to public health, jeopardizing the effectiveness of conventional antibiotics and compelling increased reliance on last-resort therapeutics (Cafaro et al., 2024). Among these, glycopeptides such as vancomycin and novel antibiotics like linezolid and tigecycline have become indispensable in managing life-threatening infections, particularly in intensive care units (ICUs), where critically ill patients are highly susceptible to MDR pathogens (Cafaro et al., 2024). Recognizing the escalating burden of AMR, the World Health Organization (WHO) and National Health Authorities have repeatedly emphasized the critical importance of rational antimicrobial use and highlighted therapeutic drug monitoring (TDM) as a central strategy to preserve drug efficacy (Cafaro et al., 2024).

TDM is a precision-guided clinical intervention that enables individualized optimization of drug dosing by quantifying antibiotic concentrations in biological matrices, predominantly plasma or whole blood (Cafaro et al., 2023). Its integration into antimicrobial stewardship programs is particularly vital for agents with narrow therapeutic indices, such as glycopeptides, whose pharmacokinetics are highly variable across patients due to differences in renal function, protein binding, and critical illness-induced pathophysiological changes (Landi et al., 2020; Pea, 2020; Pea et al., 2005). By linking pharmacokinetic (PK) parameters, including peak plasma concentration (Cmax), area under the plasma concentration–time curve over 24 h (AUC24), and trough levels (C_trough_) with pharmacodynamic (PD) indices such as the minimum inhibitory concentration (MIC), clinicians can precisely tailor therapy to maximize efficacy while minimizing toxicity (Castagnola et al., 2021; Cangemi et al., 2022; Gatti et al., 2022).

Understanding the pharmacokinetic/pharmacodynamic (PK/PD) relationship is essential because the antimicrobial effect depends not only on drug exposure but also on the pathogen’s susceptibility. Antibiotics are classified into time-dependent, concentration-dependent, or hybrid modalities (Castagnola et al., 2021; Cattaneo et al., 2020; Reed, 2000; Eyler and Shvets, 2019; Onufrak et al., 2016). Time-dependent agents exert optimal activity when plasma concentrations remain above the MIC for a substantial portion of the dosing interval (%t > MIC), making C_trough_ a key monitoring parameter (Castagnola et al., 2021; Cangemi et al., 2022; Cattaneo et al., 2020; Reed, 2000; Eyler and Shvets, 2019). The Cmax/MIC ratio best guides concentration-dependent antibiotics (Castagnola et al., 2021; Gatti et al., 2022; Cattaneo et al., 2020; Eyler and Shvets, 2019), while drugs with mixed PK/PD characteristics require evaluation of the AUC24/MIC ratio to reflect cumulative exposure relative to bacterial susceptibility (Cattaneo et al., 2020). TDM thus functions as a precision tool, safeguarding therapeutic exposure and reducing the likelihood of subtherapeutic dosing or toxicity in patients with altered physiology.

Bioanalytical methodologies are foundational to TDM, with liquid chromatography–tandem mass spectrometry (LC-MS/MS) widely regarded as the gold standard for antibiotic quantification (Cafaro et al., 2023; van der Gugten, 2020; Carvalho, 2012; Himmelsbach, 2012; Seger and Salzmann, 2020; Leung and Fong, 2014; Vogeser and Seger, 2008). LC-MS/MS combines high sensitivity and specificity with multiplexing capability, allowing simultaneous measurement of multiple drugs from minimal sample volumes, a significant advantage in vulnerable populations such as pediatrics or critically ill adults (Cafaro et al., 2023; Guerra et al., 2022; Dorofaeff et al., 2016). However, widespread clinical adoption is limited by high costs, the requirement for specialized expertise, and longer turnaround times due to complex sample preparation and analysis, which typically ranges from 5 to 10 min per sample depending on analyte properties, chromatographic conditions, and mass analyzer configuration (Cattaneo et al., 2020).

Where rapid or point-of-care monitoring is required, immunoassays provide a practical alternative, particularly when LC-MS/MS resources are unavailable (Barco et al., 2016). These assays can deliver timely concentration data, though their application is limited to select antibiotics and often requires cross-validation against LC-MS/MS for accuracy (Cattaneo et al., 2020). The integration of these bioanalytical strategies into ICU workflows enables dynamic, individualized dosing, enhancing clinical outcomes and supporting stewardship of critical antibiotics.

This review systematically aims to critically synthesize emerging bioanalytical platforms, predictive pharmacokinetic/pharmacodynamic (PK/PD) modeling frameworks, and AI-integrated decision-support systems for optimizing TDM of last-resort antibiotics, specifically vancomycin, linezolid, tigecycline, and β-lactams, with a unifying focus on bridging the analytical-clinical gap to enable precision antimicrobial therapy in critical care settings (Abdul-Aziz et al., 2020). The diversity of technologies discussed (biosensors, chromatographic innovations, wearable devices, AI/ML algorithms, and population PK models) is not incidental but deliberate: each serves the singular translational goal of real-time, precision TDM for last-resort antibiotics in critically ill patients, and their convergence represents the next frontier of antimicrobial stewardship (Rawson et al., 2021). This integrative approach is consistent with the framework adopted by leading multi-technology TDM reviews in the field (Brasier et al., 2023). Overall, the review is organized as follows: TDM at the site of infection and the three-tiered sensing model using advanced biosensor platforms. The clinical relevance of TDM for β-lactams, vancomycin, linezolid, and tigecycline in the ICU, including emerging bioanalytical techniques and population PK analysis. The critical value identification framework and AI-driven predictive TDM systems, including their current limitations. Mechanism-based PK/PD modeling and model-informed precision dosing (MIPD). Clinical trial evidence for TDM-guided precision dosing and its implications for combating AMR. The review includes future directions for clinical translation of these converging technologies.

### Literature search

We conducted a comprehensive narrative literature review, by searching such as PubMed/MEDLINE, Scopus, Google Scholar, Web of Science, Embase, and National Library of Medicine (NLM) databases respectively. A supplementary search of ClinicalTrials.gov was also conducted for registered clinical TDM trials. The search terms and Boolean strategy were applied using key MeSH and free-text terms used including therapeutic drug monitoring, TDM, antibiotics, antimicrobial resistance, vancomycin, linezolid, tigecycline, LC-MS/MS, biosensor, microneedle, wearable sensor, artificial intelligence, machine learning, population pharmacokinetics, Bayesian forecasting, PK/PD modelling, and precision dosing. Boolean operators (AND, OR, NOT) were applied to refine searches. Peer-reviewed original articles, systematic reviews, clinical guidelines, and position papers published between 1980 to till today were included. Non-English publications without available translations, conference abstracts without full-text data, and opinion pieces without supporting evidence were excluded. For article selection, the titles and abstracts were screened for relevance by three independent authors (AI, NB, HVPR). Full-text articles were reviewed for final inclusion. The final selection comprised over 240 peer-reviewed sources, of which 258 were cited in the manuscript. The literature searches were conducted between August 2025 and May 2026, with a final update completed in May 2026.

### TDM at site of infection and sensors to tackle antibiotic resistance

**FIGURE 1:**
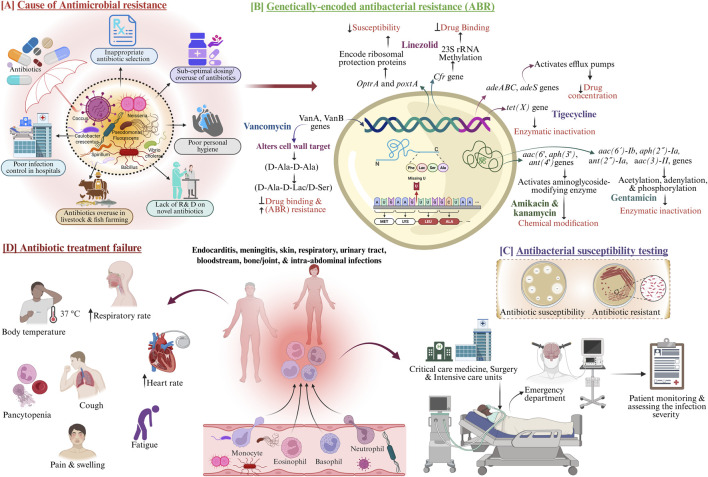
Mechanistic landscape of antimicrobial resistance and antibiotic treatment failure. This schematic illustrates the multifactorial causes and consequences of antimicrobial resistance (AMR) and antibiotic therapy failure. **(A)** Overuse and misuse of antibiotics in humans, livestock, and aquaculture, along with inadequate infection control and suboptimal dosing, accelerate resistance development. **(B)** Genetically encoded antibacterial resistance (ABR) mechanisms include target site modification, efflux pump activation, enzymatic inactivation, and drug-binding site alteration. Examples include *VanA/VanB*-mediated cell wall remodeling conferring vancomycin resistance, *Cfr*-dependent 23S rRNA methylation diminishing linezolid binding, and aminoglycoside-modifying enzymes that inactivate amikacin and gentamicin. **(C)** Antibacterial susceptibility testing distinguishes resistant from susceptible strains, guiding evidence-based therapy in critical care and surgical settings. **(D)** Antibiotic treatment failure manifests clinically as persistent infections (endocarditis, meningitis, sepsis) accompanied by systemic inflammatory symptoms such as fever, tachycardia, fatigue, and pancytopenia reflecting immunopathological dysregulation.

Multifaceted mechanisms contributing to AMR and subsequent therapeutic failure. Improper antibiotic usage, suboptimal dosing, and lack of new drug development drive the selection of resistant microbial populations. Genetically encoded mechanisms, including target modification, efflux pump activation, and enzymatic degradation, enable pathogens to evade last-resort antibiotics such as vancomycin, linezolid, tigecycline, amikacin, and gentamicin. The figure also highlights how these mechanisms reduce drug binding affinity and intracellular accumulation, leading to clinical treatment failure. Furthermore, it illustrates the importance of antimicrobial susceptibility testing in infection management and emphasizes the link between resistance, systemic inflammation, and poor treatment outcomes in critically ill patients (Figure 1). The integration of TDM directly at the site of infection (SOI) using advanced sensing platforms represents a transformative strategy to confront the escalating global challenge of antibiotic resistance (Brasier et al., 2023). This approach can be conceptualized as a three-tiered TDM model that extends conventional serum-based monitoring into a spatiotemporal, multi-compartmental framework, enabling the quantitative assessment of antibiotic distribution across interconnected physiological compartments (Brasier et al., 2023). This model systematically integrates multi-compartmental sensing to assess drug concentration dynamics in real time: (1) upstream compartment (systemic circulation): The first level focuses on the systemic bioavailability of antibiotics, traditionally assessed through blood sampling. This upstream compartment establishes baseline pharmacokinetic parameters such as peak plasma concentration, clearance, and elimination half-life. (2) local compartment (SOI microenvironment): The second level captures drug distribution at the infection site, for instance, epithelial lining fluid in pulmonary infections. This compartment is critical for understanding tissue penetration and bioavailability, which determines whether the antibiotic reaches therapeutic levels at the SOI. Real-time assessment at this level can provide direct insights into local pharmacodynamics. (3) Downstream compartment (distal biofluids): the third level involves the monitoring of drug diffusion and elimination markers in distal or excretory biofluids, such as exhaled breath condensate, sweat, saliva, tears, or urine. These fluids can be accessed non-invasively or minimally invasively and serve as surrogate matrices for real-time drug level estimation (Brasier et al., 2023). Overall, this tiered design aims to provide a real-time understanding of pharmacokinetics and tissue-specific antibiotic penetration profiles (Brasier et al., 2023).

Recent advancements in wearable biosensing technologies have created new possibilities to operationalize this multi-level TDM concept through minimally invasive or non-invasive methods, bypassing the need for centralized laboratory infrastructure. These next-generation devices include lightweight, cost-effective, and capable of continuous data acquisition, enabling precision drug monitoring in real-world clinical settings (Brasier et al., 2023). By capturing dynamic concentration gradients across biofluids, wearable sensors could facilitate individualized dosing regimens, improve therapeutic efficacy, and reduce the risk of resistance emergence through optimized antibiotic exposure windows. As proposed by Brasier et al., this integrated model represents, to the best of our knowledge, one of the earliest conceptual frameworks to propose continuous, multi-compartmental visualization of antibiotic pharmacodynamics simultaneously across blood, infection site, and distal biofluids, building upon the three-level model (Brasier et al., 2023).

Historically, over the past 6 decades, biosensors have evolved from bulky laboratory instruments to portable and miniaturized diagnostic tools, gradually replacing conventional benchtop systems in centralized laboratories (Abdul-Aziz et al., 2020). Commercial handheld point-of-care devices such as glucose and lactate meters have revolutionized self-monitoring of metabolic biomarkers, offering rapid and decentralized diagnostics (Brasier et al., 2023). However, despite their advantages, most of these biosensors rely on invasive blood sampling, which may increase the risk of infection and compromise patient adherence (Brasier et al., 2023). To overcome these barriers, current research is shifting toward the development of wearable and skin-integrated biosensors that allow for non-invasive and minimally invasive monitoring (Jager et al., 2019). These devices not only provide drug-level information but also offer broader health insights, integrating multiple physiological markers into a unified sensing interface.

A wide array of biofluids can serve as alternative analytical matrices, including sweat, (Roberts et al., 2014a; Skhirtladze et al., 2006), saliva (Shipkova and Jamoussi, 2022), tears (Nordmann et al., 2011), and interstitial fluid (ISF) (Nordmann et al., 2011; Roberts et al., 2016), which are rich in clinically relevant biomarkers and accessible without the need for venipuncture. Sweat, in particular, contains diverse electrolytes, metabolites, and xenobiotics that reflect systemic physiological states. Its high gland density (exceeding 100 glands per cm^2^ in certain regions) (Kim et al., 2019) facilitates flexible sampling strategies, while its pH and composition dynamically vary according to environmental and metabolic stimuli. Innovative sweat-monitoring platforms including temporary tattoo sensors, skin patches, wristbands, and epidermal microfluidic devices have demonstrated the ability to track small-molecule analytes such as glucose, alcohol, uric acid, tyrosine, and cortisol, with reported correlations to blood concentrations (Roberts et al., 2014a; Wang et al., 2022; Yang et al., 2020; Sempionatto et al., 2021; Lee et al., 2016). Furthermore, sweat-based sensing is being explored for the detection of exogenous compounds, including therapeutic drugs (Brasier and Eckstein, 2020). Yet, the mechanistic understanding of the pharmacokinetic relationship between blood and sweat remains incomplete, necessitating further research on diffusion kinetics, biofluid partitioning, and inter-individual variability.

Parallel to sweat sensing, breath analysis has emerged as another non-invasive platform for drug monitoring. The discovery of volatile organic compounds in exhaled breath associated with metabolic processes has stimulated the development of gas sensor arrays, or “electronic noses,” which mimic olfactory receptor function (Khatib and Haick, 2022). These platforms have been successfully used in the clinical management of respiratory conditions such as asthma and for the non-invasive diagnosis of systemic diseases including diabetes, viral infections (Bordbar et al., 2022), and lung cancer (Chen et al., 2020). Additionally, the detection of respiratory biomarkers such as hydrogen peroxide via paper-based sensing systems integrated into face masks (Maier et al., 2019) exemplifies the potential of this technology in infectious disease monitoring. Groundbreaking work has demonstrated nucleic acid detection of COVID-19 directly from exhaled breath using CRISPR-based wearable biosensors (Nguyen et al., 2021). Notably, studies monitoring antibiotic pharmacokinetics in exhaled breath condensate (Ates et al., 2022) reveal parallel transport behaviors with blood, suggesting the lung-blood interface as an efficient conduit for non-invasive drug level assessment.

Saliva is another attractive alternative matrix due to its ease of collection and large daily output (Zheng et al., 2021). However, its clinical utility is complicated by challenges including microbial contamination, food debris interference, and drug ion-trapping phenomena. Sensor biofouling remains a key limitation, although recent material engineering advances aim to enhance sensor biocompatibility. Similarly, urine, a metabolic byproduct of renal excretion can be efficiently sampled and analyzed using lateral flow devices (Kenaan et al., 2020; Chan et al., 2022). Because recurrent urinary tract infections often involve multidrug-resistant pathogens, real-time urinary antibiotic monitoring could play a pivotal role in improving treatment outcomes and reducing antimicrobial misuse.

Tear fluid offers distinct advantages as a biomatrix because of its low and stable volume, continuous replenishment, and relatively low propensity for biofouling compared to saliva, ISF, or sweat (Jeon et al., 2021). Tear-based sensing technologies, often integrated into contact lenses, are being developed to monitor ocular biomarkers and to support early detection and treatment of ocular infections (Jeon et al., 2021; Ma et al., 2021). Likewise, stool analysis has become a powerful tool for assessing gut microbiota, evaluating probiotic colonization, and diagnosing inflammatory bowel conditions (Karu et al., 2018). Its potential application for antibiotic pharmacokinetic monitoring in patients with altered gut transit represents an emerging frontier in personalized antimicrobial therapy.

In summary, integrating TDM across upstream, local, and downstream compartments using non-invasive sensing technologies could fundamentally redefine antibiotic stewardship. By moving beyond static, blood-centric models toward continuous, wearable-enabled biofluid monitoring, clinicians can achieve more precise dosing strategies, reduce resistance development, and improve patient outcomes. This systems-level approach bridges pharmacology, bioengineering, and clinical microbiology, heralding a new era of spatiotemporal precision in antimicrobial therapy. The proposed multi-level TDM framework is designed to provide a systems-level approach for continuous antibiotic monitoring across various biological matrices, providing a comprehensive, spatiotemporal view of antibiotic pharmacokinetics at and around the site of infection (Brasier et al., 2023).

This tri-compartmental approach departs from conventional, blood-only TDM strategies by incorporating wearable and skin-integrated biosensing systems capable of continuous, autonomous data collection. Moreover, integrating these data streams with machine learning-based pharmacokinetic modeling can allow for individualized dose adjustment and prediction of drug behavior in complex physiological states (Brasier et al., 2023). The translation of this three-tiered TDM model into clinical practice holds the potential to revolutionize antimicrobial therapy and combat antibiotic resistance. First, by enabling continuous monitoring of drug levels in real time, clinicians can ensure that antibiotic exposure remains within the optimal therapeutic window maximizing efficacy while minimizing subtherapeutic dosing that drives resistance selection. Second, real-time drug concentration feedback allows personalized dosing strategies for patients with variable pharmacokinetic profiles (e.g., critically ill, elderly, or patients with organ dysfunction), addressing the heterogeneity in tissue penetration often overlooked by standard dosing regimens. This can be particularly impactful in conditions where plasma concentrations do not accurately reflect drug levels at the SOI, such as in pulmonary infections, urinary tract infections, or gut-related pathologies (Brasier et al., 2023). Third, the use of non-invasive biosensing platforms can significantly reduce patient burden, improve compliance, and decrease hospital visits, making TDM feasible not only in intensive care units but also in outpatient and resource-limited settings. Furthermore, integrating these sensors with digital health platforms can enable remote monitoring, telemedicine-driven dose optimization, and earlier detection of treatment failure. Lastly, by providing a comprehensive picture of drug pharmacokinetics across multiple physiological compartments, this framework supports more rational antimicrobial stewardship programs, improves clinical decision-making, and helps delay the emergence of multidrug-resistant organisms (Brasier et al., 2023).

### Analytical validation of a versatile biosensor for on-site TDM

To enable the seamless clinical integration of TDM, antibiotic levels can be accurately determined using either blood-based point-of-care testing (POCT) or non-invasive biofluid sampling strategies. In this context, a versatile polymer-based biosensing platform has been developed, employing an antibody-free assay for real-time, on-site antibiotic quantification (Brasier et al., 2023). The analytical performance of the biosensor was rigorously assessed in a controlled animal study, where antibiotic concentrations were determined across multiple biological matrices, including whole blood, plasma, saliva, urine, and exhaled breath condensate (EBC) (Brasier et al., 2023). This multi-matrix approach enabled a comprehensive pharmacokinetic assessment of drug distribution and clearance *in vivo*. Temporal evaluation of antibiotic levels in both plasma and EBC demonstrated the platform’s capacity to capture dynamic clearance profiles with high temporal resolution. Matrix-dependent variations in measured concentrations were systematically analyzed by comparing plasma reference values with those from non-invasive samples, thereby elucidating biofluid-specific transport and partitioning phenomena (Brasier et al., 2023). In addition, the biosensor demonstrated the ability to track β-lactam concentrations in untreated whole blood samples, supporting its feasibility as a POCT device suitable for bedside or decentralized clinical use. Multiplexing functionality was further validated by simultaneous multianalyte and multi-sample analysis, paving the way for high-throughput, real-time monitoring in personalized antibiotic therapy (Brasier et al., 2023).

### Current challenges in breath-based antibiotic detection

Recent innovations have focused on developing non-invasive strategies for antibiotic quantification, particularly in exhaled breath condensate. For example, a fluorescence spectrometry technique using copper nanocrystals has been applied to measure vancomycin levels in EBC following drug administration (Rahimpour et al., 2021). Although spike–recovery tests confirmed detectability, temporal drug profiling and plasma-EBC correlation analyses were not performed. Similarly, UV–visible spectroscopy has been used to detect tobramycin in the breath of healthy volunteers after controlled inhalation (Khoubnasabjafari et al., 2021); however, this study also did not investigate correlations between plasma and EBC concentrations. Chromatographic methods have been explored to quantify antibiotics in EBC samples from patients treated with piperacillin/tazobactam or meropenem, employing ultrahigh-pressure liquid chromatography coupled with high-resolution mass spectrometry (Herregodts et al., 2019). While preliminary correlation analyses were conducted, no clinically reliable relationship between EBC and plasma levels was established.

To date, in-depth biosensor-based investigations examining time-resolved correlations between antibiotic levels in EBC and plasma remain scarce. Consequently, there is no standardized interpretive framework to guide clinical translation of EBC-derived TDM data. This lack of consensus raises several critical clinical questions that must be addressed to advance precision antimicrobial therapy. First, it is essential to determine whether antibiotic concentrations can be measured with adequate sensitivity to enable reliable PK/PD modeling. Second, understanding how transport mechanisms and matrix effects impact drug concentration measurements in non-invasive biological samples is crucial for ensuring analytical accuracy. Finally, establishing a quantitative correlation between blood and non-invasive fluid levels on a single analytical platform could transform therapeutic drug monitoring, enabling real-time, patient-specific dosing strategies.

### Pharmacokinetic correlation using an advanced microfluidic biosensor

To address these unresolved questions, an advanced microfluidic biosensing platform integrating synthetic-biology-enabled, antibody-free β-lactam detection chemistry was developed and validated to enable real-time, multi-matrix pharmacokinetic correlation analysis (Ates et al., 2022). This platform was validated in preclinical studies on Landrace pigs, receiving under-, standard-, and over-dosed regimens of piperacillin/tazobactam. For the first time, real-time detection and temporal monitoring of piperacillin/tazobactam in EBC were demonstrated, accompanied by a systematic plasma–EBC correlation analysis (Ates et al., 2022). The biosensor is capable of detecting drug concentrations in the ng mL^-1^ range, significantly exceeding the sensitivity of conventional chromatographic techniques (Ates et al., 2022; Trittler et al., 2002). Benchmarking against HPLC, the current gold standard confirmed excellent analytical concordance in plasma measurements (Ates et al., 2022). The influence of matrix effects was further explored through parallel measurements in plasma, EBC, saliva, and urine, enabling a more nuanced understanding of inter-matrix drug kinetics. Whole blood samples were analyzed without any pre-treatment, demonstrating the platform’s potential for rapid, point-of-care deployment in both hospital and outpatient settings (Ates et al., 2022).

### Multiplexing capability and translational clinical potential

A major advantage of this microfluidic biosensor lies in its multiplexing capacity, which allows the simultaneous quantification of multiple β-lactam antibiotics and multi-matrix analysis on a single chip. For example, piperacillin/tazobactam levels were measured in plasma, EBC, saliva, and urine simultaneously, enabling the creation of cross-correlation databases that can inform precision dosing algorithms (Ates et al., 2022). This integrated, low-cost, and high-sensitivity TDM platform has the potential to transform clinical practice by moving away from empirical dosing toward real-time, individualized therapeutic optimization. By overcoming the limitations of traditional, invasive, and laboratory-dependent assays, it lays the foundation for a next-generation antimicrobial stewardship framework, one that is proactive, data-driven, and clinically actionable (Ates et al., 2022).

### Antimicrobial resistance and antibiotic optimization by TDM

AMR represents one of the most critical global health threats of the 21st century, undermining decades of medical advancements and posing severe challenges to patient outcomes and healthcare systems worldwide (Naghavi et al., 2024). A cornerstone of AMR mitigation involves precision-guided antibiotic dosing, with TDM serving as a central tool to optimise drug exposure and minimise resistance development (Rawson et al., 2021) by ascertaining quantitative measurement of antibiotic plasma concentrations to guide dosing adjustments, thereby achieving optimal therapeutic windows while avoiding subtherapeutic exposure or toxic accumulation.

Among currently available antimicrobials, vancomycin remains a first-line agent for treating life-threatening Gram-positive infections, including methicillin-resistant Staphylococcus aureus (MRSA), where maintaining steady-state concentrations within the narrow therapeutic index is vital for both clinical efficacy and renal safety (Rybak et al., 2020; Rose et al., 2022). However, the clinical impact of TDM is often curtailed by the intrinsic delays of conventional laboratory workflows. Standard TDM techniques are dependent on centralised analytical platforms, and results frequently take several hours, delaying timely dose optimisation (Mabilat et al., 2020). Currently, widely used assays for vancomycin quantification such as LC/MS, ELISA, and fluorescence-based immunoassays are technically robust but often cost-intensive, centralised, and time-consuming (Bian et al., 2021). These assays usually involve sample transportation and laboratory processing, with turnaround times of 2–3 h and actual clinical dose modification occurring 12 h or more post-collection (Klapkova et al., 2020). Despite technological progress, there remains an unmet need for rapid, decentralised, and cost-efficient TDM strategies that can be deployed at the bedside.

Recent advances in point-of-care (POC) diagnostic technologies provide a potential paradigm shift in antibiotic stewardship. Lateral flow assays (LFAs), historically used for qualitative diagnostics, align strongly with the WHO ASSURED criteria (affordable, sensitive, specific, user-friendly, rapid, robust, equipment-free, and deliverable) (Calucho et al., 2020; Liu et al., 2021). Emerging portable detection modalities such as smartphone-integrated imaging have further expanded LFAs into quantitative detection platforms for antibiotic monitoring (Urusov et al., 2019). A typical LFA consists of a nitrocellulose membrane coated with immobilised biomolecules, functioning in either sandwich or competitive assay configurations. Because vancomycin is a small molecule, competitive assay formats are required (Calucho et al., 2020; Nuntawong et al., 2022; Sajid et al., 2015). Although traditional competitive LFAs can generate rapid quantitative signals, they often suffer from narrow dynamic ranges and dependence on external detectors, making accurate detection at clinically relevant vancomycin levels (15–20 mg/L) challenging (Bian et al., 2021; Sajid et al., 2015; Jacinto et al., 2018). To address this, previous authors (Riezk et al., 2025) designed an innovative competitive LFA incorporating two test lines an antibody line and an avidin line to improve sensitivity and broaden the quantifiable range. This dual-line format leverages biotin-avidin interactions to capture excess labeled conjugate, enabling differential signal analysis between the two lines and enhancing analytical accuracy across a broader concentration range (Riezk et al., 2025). In phase one, the assay was designed and optimised for serum-based vancomycin quantification and benchmarked against standard ELISA and conventional competitive LFA platforms. In phase two, spiked serum samples were used to validate assay precision, reproducibility, specificity, and practical TDM applicability (Riezk et al., 2025).

Conventional single-line LFAs showed limited sensitivity and narrow detection ranges, often below the clinically relevant threshold for effective TDM (Riezk et al., 2025). This limitation arises from the inverse signal-concentration relationship intrinsic to competitive formats, where increasing vancomycin concentrations displace the labeled conjugate from the antibody site, resulting in lower signal intensities at higher concentrations (Riezk et al., 2025). Consequently, traditional LFAs require impractical sample dilutions (2,000–5,000 fold) to detect therapeutic trough levels (Rybak et al., 2020; Verch et al., 2016; Li W. et al., 2024). Such dilution introduces significant pipetting variability, compromising reliability in real-world POC environments. Additionally, conventional LFAs are highly susceptible to environmental variability, including illumination conditions, camera parameters, and angle of detection, often leading to coefficients of variation exceeding 20% (Li W. et al., 2024; Cheng et al., 2017; Li et al., 2017; Scherr et al., 2016; Xiao et al., 2018; Ruppert et al., 2019; Guler et al., 2017; Saisin et al., 2018; Park et al., 2022). While some assays attempt to mitigate this through test-to-control line signal ratios, these control lines typically act only as procedural checks, with minimal contribution to analytical accuracy (Li et al., 2019). In contrast, dual-line LFA design introduces an avidin-coated secondary test line, which produces a positive concentration-dependent signal by capturing free biotinylated conjugate (Riezk et al., 2025). The ratio between the two test lines, rather than absolute intensity, forms the analytical basis greatly improving robustness against environmental and instrumental variability (Riezk et al., 2025). This internal ratiometric normalisation compensates for variations in external illumination or camera performance, maintaining analytical fidelity even under ambient clinical conditions (Riezk et al., 2025). By integrating this dual-line strategy with a custom R-based image analysis algorithm, the assay achieved enhanced sensitivity, expanded detection range, and environmental stability, making it a strong candidate for near-patient vancomycin monitoring (Riezk et al., 2025). Future development will involve clinical validation with patient-derived samples, comparative benchmarking with gold-standard LC-MS/MS, and stability testing under real-world field conditions. Incorporating a dedicated internal control line with orthogonal signal reporters may further enhance assay reliability (Riezk et al., 2025). In summary, this work introduces a next-generation competitive dual-line LFA for vancomycin TDM, combining quantitative analytical performance with point-of-care deployability. The platform’s speed, cost-effectiveness, and robustness position it as a transformative tool for antimicrobial stewardship, enabling precision dosing and potentially reducing AMR propagation through optimised antibiotic exposure (Riezk et al., 2025).

However, the clinical utility of conventional TDM methods such as LC/MS and ELISA is limited by centralised processing, extended turnaround times, and cost constraints (Mabilat et al., 2020; Bian et al., 2021; Klapkova et al., 2020). These delays often result in suboptimal dose adjustments, compromising both efficacy and safety, and potentially accelerating the development of resistance. Future steps include clinical validation with patient samples, head-to-head comparisons with gold-standard LC-MS/MS methods, and integration into existing stewardship programs. This dual-line LFA platform represents a clinically translatable, scalable solution to enhance precision antibiotic therapy and address the global AMR crisis (Riezk et al., 2025).

### Clinical relevance of TDM of β-Lactam antibiotics, tetracycline antibiotics, vancomycin, linezolid, and tigecycline in ICU settings

TDM of antibiotics is increasingly recognized as an essential component of precision antimicrobial therapy, particularly in critically ill patients admitted to ICUs. In these settings, altered pathophysiological states such as augmented renal clearance, hypoalbuminemia, capillary leak, and dynamic fluid shifts significantly modify drug pharmacokinetics and pharmacodynamics. These changes can lead to subtherapeutic exposures or toxicity when standard dosing regimens are applied. Hence, TDM plays a pivotal role in individualized dosing, ensuring optimal therapeutic efficacy and minimizing the emergence of AMR.

### TDM of β-lactam antibiotics to tackle antimicrobial resistance

β-Lactam antibiotics, including penicillins, cephalosporins, monobactams, and carbapenems, remain the cornerstone of empirical therapy in ICU patients. Achieving and maintaining adequate plasma concentrations relative to the MIC is critical to prevent therapeutic failure and resistance selection. A robust analytical infrastructure is required to support real-time, high-throughput TDM. In a previous report (Colin et al., 2013). A UPLC–MS/MS platform capable of simultaneously quantifying 12 β-lactam antibiotics in human plasma was developed. The assay used a mixed-mode SPE extraction protocol, demonstrating exceptional analytical performance with low LOQs, minimal matrix interference, and high reproducibility. This strategy enables rapid and reliable β-lactam measurement, supporting dose individualization. However, clinical translation requires validation in larger patient populations with variable pharmacokinetic profiles.

Similarly, Ohmori et al. (2011) described an LC–MS/MS method for eight β-lactams in serum using a high-resolution octadecyl silica column and SPE preparation. This method exhibited broad linearity, low LOQs, and high recovery (>80%) for most analytes, except doripenem and meropenem. Such variability in recovery describes the need for method optimization for specific β-lactams, especially those with unstable structures or low protein binding in critically ill patients. Šestáková et al. (2017) compared micellar electrokinetic capillary chromatography (MEKC) and LC–MS/MS for cefepime quantification in serum and plasma, demonstrating comparable analytical performance and validating MEKC as an alternative tool for routine TDM. This is clinically significant, as cefepime underexposure is a known predictor of poor outcomes in septic shock. Addressing specific populations, a HILIC/ESI-MS method for cefuroxime, cefoxitin, and cefazolin detection in breast milk and plasma was reported (Kiriazopoulos et al., 2017), enabling pharmacokinetic evaluation in lactating patients, a clinically relevant but often overlooked population. Bellouard et al. (2020) further expanded analytical capability by developing a rapid LC–MS/MS assay for simultaneous quantification of eight β-lactams in plasma and CSF. This method demonstrated broad applicability in over 2,200 clinical plasma samples, highlighting the feasibility of implementing advanced LC–MS/MS workflows in high-volume clinical laboratories.

### Analytical advancements supporting real-time ICU TDM

A rapid HPLC-UV platform for simultaneous detection of 12 β-lactams was developed by Verdier et al. (2011), offering accuracy and reproducibility with <8% CV and minimal interference, allowing integration into routine ICU workflows. Stability studies by Martens-Lobenhoffer et al. (Verdier et al., 2011) revealed that ceftolozane remains stable in plasma at refrigerated and frozen temperatures but degrades at ambient conditions, emphasizing the importance of timely sample transport in real-world ICU settings. Abdulla et al. (2017) developed an ultrafast HILIC–UPLC–MS/MS method targeting nine β-lactams in plasma, achieving high sensitivity and precision suitable for critically ill patients, where dynamic clearance profiles demand frequent monitoring. Volumetric absorptive microsampling (VAMS) has emerged as an alternative to traditional DBS. Moorthy et al. (2020) demonstrated a HPLC–MS/MS method for cefepime quantification using VAMS with low matrix effects and acceptable stability, offering a minimally invasive TDM strategy suitable for frequent sampling in ICU patients with limited vascular access. Rigo-Bonnin et al. (2017) introduced a UPLC–MS/MS workflow for β-lactams in patients on continuous infusion, enabling real-time pharmacokinetic adjustment. Likewise, Magréault et al. (2022) established a high-throughput LC–MS/MS platform for 10 β-lactams using automated protein precipitation and online sample handling, reducing turnaround time and supporting near-real-time therapeutic decision-making in septic patients.

### Extending TDM to other critical antibiotics in ICU

Beyond β-lactams, TDM of tetracyclines, vancomycin, linezolid, and tigecycline is also clinically crucial. Vancomycin TDM is well-established to minimize nephrotoxicity and ensure therapeutic exposure, particularly against *Staphylococcus aureus* (MRSA). Linezolid, with variable clearance in critically ill patients, benefits from TDM to prevent hematological toxicity and therapeutic failure. Tigecycline, characterized by extensive tissue distribution and variable serum concentrations, requires TDM to avoid subtherapeutic exposures in severe infections. Tetracyclines, though less commonly monitored, may require TDM in patients with altered clearance or those on extracorporeal support.

Model framework and clinical workflow for TDM of critical antibiotics in ICU settings: To achieve precision antimicrobial therapy in critically ill patients, a structured and adaptive TDM framework is required to integrate analytical data with real-time clinical decision-making (Kousi et al., 2025). This model involves three interconnected layers: (1) Pharmacological assessment layer: this foundational layer involves identifying antibiotic classes that exhibit high pharmacokinetic variability, narrow therapeutic indices, or PK/PD targets that strongly influence clinical outcomes (Kousi et al., 2025). Key antibiotic groups include β-lactams, vancomycin, linezolid, tigecycline, and tetracyclines. In ICU patients, pathophysiological factors such as augmented renal clearance, fluid overload, sepsis-induced capillary leak, extracorporeal support, and dynamic organ dysfunction alter drug distribution and clearance (Kousi et al., 2025). Therefore, individualized TDM strategies are triggered early in the treatment course (usually within the first 24–48 h of therapy). At this stage, the pharmacodynamic targets (e.g., %fT > MIC for β-lactams, AUC/MIC for vancomycin or linezolid, or steady-state concentration for tigecycline) are defined. Real-time physiological parameters, renal function indices, and infection severity scores are incorporated into dose prediction algorithms (Kousi et al., 2025). (2) Analytical quantification layer: This layer integrates advanced bioanalytical platforms with clinically viable sampling strategies. Plasma remains the gold standard; however, complementary use of non-invasive and microsampling techniques (e.g., VAMS, saliva, exhaled breath condensate, and interstitial fluid) improves patient comfort and enables serial sampling in unstable patients. High-performance platforms such as LC–MS/MS, HILIC–UPLC–MS/MS, or MEKC provide the accuracy and sensitivity necessary for detecting antibiotics at subtherapeutic, therapeutic, and supratherapeutic levels. Automated protein precipitation and microfluidic-based platforms allow high-throughput and low-turnaround analysis, which is critical for ICU settings where therapeutic windows are short. Emerging point-of-care biosensors and lateral flow immunoassays are integrated at bedside to generate real-time data, which can be transmitted to clinical decision-support systems for immediate interpretation. (3) Clinical translation layer: The final layer transforms TDM data into actionable clinical interventions. Measured concentrations are mapped against target PK/PD indices, allowing dose optimization in real time. Decision-support algorithms or Bayesian forecasting models can automatically suggest dose escalation, de-escalation, or interval modification based on patient-specific pharmacokinetics (Kousi et al., 2025).

**FIGURE 2:**
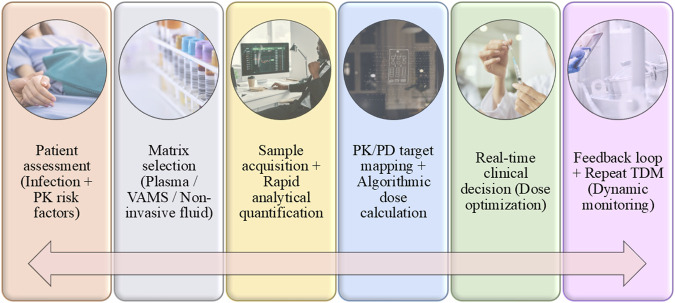
Schematic representation of a closed-loop precision therapeutic drug monitoring (TDM) workflow for optimizing last-resort antibiotic therapy in critically ill patients. The framework integrates patient assessment, matrix selection, rapid bioanalytical quantification, PK/PD-guided dose calculation, and real-time clinical dose optimization to enable individualized antimicrobial exposure. Continuous feedback through repeat TDM and dynamic monitoring supports adaptive dosing, improves antimicrobial stewardship, and minimizes the emergence of antimicrobial resistance.

A closed-loop workflow can be established (Figure 2):

- Sample collection (plasma, VAMS, or alternative matrices) →
- Rapid quantification (on-site or near-patient platforms) →
- PK/PD model integration and interpretation →
- Dose adjustment (guided by clinical pharmacists and intensivists).

This framework not only enhances antimicrobial exposure precision but also contributes to antimicrobial stewardship, minimizing the emergence of resistant organisms while improving clinical outcomes.

### Conceptual workflow diagram

This structured approach can be integrated into electronic health record systems to facilitate automated alerts for subtherapeutic or supratherapeutic exposures and guide antimicrobial stewardship programs in ICU environments (Kousi et al., 2025).

### TDM of tetracyclines and last-resort antibiotics in critical care settings

#### TDM of tetracyclines to tackle AMR

TDM represents a cornerstone of precision medicine for antimicrobial agents with narrow therapeutic indices and variable pharmacokinetics in critically ill patients. Among tetracyclines, omadacycline has gained prominence as a next-generation (Wang et al., 2025), semi-synthetic antibiotic with activity against multidrug-resistant pathogens. Quantification of drug exposure through TDM is essential to optimize dosing and suppress resistance emergence.

Liquid chromatography–tandem mass spectrometry (LC–MS/MS) provides superior analytical sensitivity and specificity, enabling the reliable detection of single peaks within the complex milieu of blood matrices (Wang et al., 2025). In this context, an optimized LC–MS/MS protocol was established employing acetonitrile as a protein precipitant and 0.1% formic acid in aqueous phase to enhance chromatographic peak resolution. Separation on a 50 mm C18 reverse-phase column allowed rapid retention of omadacycline with reduced run time, meeting regulatory limits for LLOQ with high analytical accuracy, precision, and minimal matrix interference (Wang et al., 2025). This methodology fulfils rigorous clinical validation requirements and is suitable for routine implementation in hospital laboratories (Wang et al., 2025).

In a clinical evaluation of 127 patients, plasma omadacycline levels post-intravenous infusion were distributed within the expected therapeutic range, with observed variability across individuals. Previous reports indicate peak plasma concentrations of approximately 1,990 ng/mL in healthy adults under similar dosing regimens (Yang et al., 2022). In contrast, this study found marginally reduced mean concentrations, likely influenced by patient-specific physiological alterations such as advanced age, severe pneumonia, comorbidities, and altered drug distribution volumes. Gender differences have also been documented, with women demonstrating higher plasma concentrations than men (Wang et al., 2024), a finding relevant to patient stratification in real-world clinical settings. Additional variability arises from diet and other uncontrolled clinical variables (Tzanis et al., 2017). Limitations included the lack of isotopic internal standards and the absence of standardized dosing timing among participants, which may have contributed to observed inter-individual differences.

Notably, few studies have defined a clear reference range for omadacycline plasma concentrations. Existing data are primarily derived from preclinical investigations of AUC/MIC targets against common respiratory pathogens (Lepak et al., 2017; Rodvold and Pai, 2019; Lepak et al., 2019). Establishing robust PK/PD breakpoints through well-designed clinical pharmacokinetic studies is therefore an urgent research priority. Future work should integrate longitudinal TDM, real-world pharmacodynamic analyses, and population PK modeling to establish individualized dosage algorithms for critically ill patients receiving omadacycline therapy.

Mechanistically, omadacycline evades classical tetracycline resistance mechanisms, including efflux pumps and ribosomal protection, thereby maintaining efficacy against resistant strains (Durães and Sousa, 2019). As a time-dependent antibiotic, its clinical efficacy correlates strongly with the 24-h area under the plasma concentration–time curve to minimum inhibitory concentration (AUC_0_–_24_ h/MIC) ratio (Yaghoubi et al., 2022). However, critically ill patients often exhibit altered pharmacokinetic behavior due to capillary leak, fluid shifts, and organ dysfunction, necessitating TDM-driven adaptive dosing (Annoni et al., 2020; Taccone et al., 2010; Jacobs et al., 2018; Beumier et al., 2014; Joukhadar et al., 2001; Donadello et al., 2015). LC–MS technology remains the gold standard for quantification (Seger and Salzmann, 2020), though few validated methods for omadacycline quantitation exist (Flarakos et al., 2017; Suhang et al., 2024; Hu et al., 2024). The development of rapid, sensitive, and clinically deployable LC–MS platforms will facilitate PK/PD-guided dose individualization in this high-risk population (Wang et al., 2025).

#### TDM of vancomycin, linezolid, and tigecycline to combat AMR

Vancomycin, linezolid, and tigecycline are key agents in the treatment of multidrug-resistant infections, including those caused by MRSA, VRE, and CRE. Their use is particularly prevalent in ICUs, where pharmacokinetics are profoundly influenced by pathophysiological changes such as augmented renal clearance, hypoalbuminemia, vasopressor use, extracorporeal support, and dynamic fluid shifts. These factors significantly affect drug disposition and exposure profiles, thereby complicating standardized dosing strategies.

#### Vancomycin

Vancomycin remains the gold standard for MRSA infections but is associated with notable limitations including slow bactericidal activity, tissue penetration challenges, nephrotoxicity, and emerging resistance (Revilla et al., 2010; Edwards et al., 2012). PK/PD modeling identifies an AUC/MIC ratio ≥400 as the critical efficacy threshold (Brink, 2012). In ICU patients, vancomycin disposition is highly variable (Pea et al., 2005), leading to frequent subtherapeutic exposure when standard dosing is applied. Population pharmacokinetic (PPK) modeling and Bayesian dose optimization offer a precise approach to individualizing therapy and reducing mortality in patients with difficult-to-treat infections (Ibrahim et al., 2000; Alqahtani et al., 2018) (Table 1). Studies have demonstrated covariate influences such as body weight, creatinine clearance, burn injury, and dopamine co-therapy on clearance and volume of distribution (Alqahtani et al., 2018; Okada et al., 2018), supporting the need for model-based TDM in this subgroup (Lin et al., 2021).

**TABLE 1:** Emerging bioanalytical strategies and clinical implications for precision antibiotic therapeutic drug monitoring (TDM) and AMR mitigation.

Novel bioanalytical strategy/antibiotic	Method/analytical platform	Implication in TDM	Implication to enhance precision dosage therapy against AMR-resistant bacteria	Refs
Polymer-based microfluidic biosensor for β-Lactams (Piperacillin/Tazobactam)	Antibody-free synthetic-biology-enabled electrochemical detection	Enables real-time, multiplexed quantification of β-lactams across plasma, saliva, urine, and EBC; supports on-site monitoring without pre-treatment	Facilitates dynamic dose adjustment, prevents subtherapeutic exposure, and optimizes PK/PD targets for resistant Gram-negative infections	Brasier et al. (2023), Ates et al. (2022), Trittler et al. (2002)
Fluorescence spectrometry for Vancomycin	Copper nanocrystal-assisted fluorescence spectroscopy	Demonstrates feasibility of non-invasive vancomycin detection in exhaled breath condensate (EBC)	Promotes localized pulmonary drug exposure monitoring, guiding optimized dosing against MRSA and VRSA infections	Rahimpour et al. (2021)
UV–Visible spectroscopy for Tobramycin	Breath-based optical quantification	Enables rapid, non-invasive antibiotic screening from inhaled aerosols	Provides insights into drug absorption at respiratory infection sites, supporting targeted therapy against Pseudomonas aeruginosa	Khoubnasabjafari et al. (2021)
UHPLC–HRMS for β-Lactams (Meropenem, Piperacillin/Tazobactam)	Ultrahigh-pressure liquid chromatography–high-resolution mass spectrometry	Quantifies antibiotic levels in exhaled breath condensate with high analytical resolution	Advances in translational PK correlation between plasma and pulmonary biofluids, enabling precision therapy for ventilator-associated infections	Herregodts et al. (2019)
Dual-Line competitive lateral Flow Assay (LFA) for Vancomycin	Biotin–avidin enhanced dual-test line format with R-based image quantification	Achieves rapid bedside detection with improved sensitivity, reproducibility, and environmental robustness	Provides decentralized, low-cost monitoring of vancomycin exposure to minimize nephrotoxicity and resistance emergence in MRSA therapy	Bian et al. (2021), Calucho et al. (2020), Nuntawong et al. (2022), Sajid et al. (2015), Jacinto et al. (2018), Riezk et al. (2025)
Volumetric Absorptive Microsampling (VAMS) for β-Lactams	LC–MS/MS analysis coupled with microsampling	Minimally invasive sampling with reduced matrix interference, suitable for frequent ICU monitoring	Enables individualized dose titration in critically ill patients with variable clearance, enhancing antimicrobial stewardship	Moorthy et al. (2020)
HILIC–UPLC–MS/MS for multiple β-Lactams	Automated protein precipitation and hydrophilic interaction chromatography	Rapid, high-throughput quantification for multi-drug TDM	Supports near-real-time therapeutic optimization and stewardship in septic ICU patients	Abdulla et al. (2017), Magréault et al. (2022)
Wearable biosensing devices (e.g., Sweat/Interstitial Fluid Sensors)	Continuous electrochemical or optical sensing integrated into skin patches	Provides non-invasive, continuous monitoring of systemic and tissue drug concentrations	Enhances compliance and real-time therapy adjustment, reducing risk of resistance in chronic infection management	Brasier et al. (2023), Jager et al. (2019)
Tear-integrated biosensors for ocular antibiotics	Contact lens–based sensing platform	Enables continuous, local-level antibiotic detection for ocular infections	Improves therapeutic targeting and early detection of resistance at ocular sites	Jeon et al. (2021), Ma et al. (2021)
Three-tiered spatiotemporal TDM model	Multi-compartmental (upstream, local, downstream) biosensing integration	Offers real-time pharmacokinetic mapping across blood, infection site, and distal fluids	Provides systems-level control over antibiotic exposure, mitigating resistance via tissue-targeted dosing	Brasier et al. (2023)
AI-Integrated Bayesian forecasting framework	Machine learning–based PK/PD predictive modelling	Converts biosensor data into dose predictions for individualized therapy	Enables automated, adaptive dosing to sustain optimal drug levels and suppress resistance evolution	Kousi et al. (2025)

Emerging point-of-care solutions, such as quantitative lateral flow assays, are being developed to enable decentralized vancomycin monitoring. A dual-line competitive binding design with gold nanoparticle–vancomycin conjugates allows smartphone-based quantification over a dynamic range offering high analytical accuracy and clinical applicability for rapid dose adjustments (Riezk et al., 2025).

#### Linezolid and tigecycline

Linezolid’s PK/PD efficacy is primarily driven by time above MIC, while tigecycline’s therapeutic success correlates with AUC/MIC (El-Gaml et al., 2022; Hui et al., 2022; Hui et al., 2024; De Pascale et al., 2014). Both drugs exhibit extensive distribution, and their pharmacokinetics are strongly affected by altered protein binding and organ function in critical illness. Drug interactions particularly nephrotoxic or serotonergic co-medications further complicate optimal dosing. TDM of linezolid can minimize hematological and neurologic toxicities, while tigecycline TDM can help counteract variable exposure seen in patients on renal replacement or extracorporeal support (Rao et al., 2020; Tietjen et al., 2022).

In critically ill patients, fixed-dose regimens are inadequate due to large inter- and intra-patient pharmacokinetic variability. TDM provides a dynamic, evidence-based framework for individualized dosing, allowing clinicians to: (1) achieve PK/PD targets (e.g., AUC/MIC ≥400 for vancomycin, time above MIC for linezolid, AUC/MIC for tigecycline and omadacycline). (2) reduce toxicity risk through early detection of drug accumulation. (3) optimize efficacy in the presence of organ dysfunction or extracorporeal support. (4) support antimicrobial stewardship, minimizing resistance emergence (Rao et al., 2020; Tietjen et al., 2022).

The integration of advanced analytical methods such as LC–MS/MS, microfluidic biosensors, and lateral flow devices with clinical PK/PD modeling represents a critical evolution in precision antimicrobial therapy in the ICU (Rao et al., 2020; Tietjen et al., 2022). These approaches will support real-time therapeutic decision-making, improve patient outcomes, and contribute to global efforts against antimicrobial resistance.

### Emerging bioanalytical techniques for TDM

Accurate quantification of antibiotics in biological matrices is essential for TDM, which relies on bioanalytical methods that combine sensitivity and selectivity. Traditional TDM analytical platforms include HPLC, LC-MS/MS, and immunoassay-based techniques. The selection of an appropriate analytical method depends on clinical requirements, desired sensitivity, turnaround time, availability of instrumentation, and matrix complexity. Commercially available vancomycin assays, such as LC/MS, ELISA, and fluorescence-based detection, often involve centralized laboratory processing (Bian et al., 2021). This process entails sample transportation, resulting in a turnaround time of 2–3 h, while dose adjustments may not occur until 12 h post-collection (Klapkova et al., 2020). Despite technological advances, improvements in assay speed, simplicity, and cost-effectiveness are limited (Klapkova et al., 2020), highlighting the need for decentralized, rapid, and user-friendly platforms for point-of-care vancomycin monitoring (Calucho et al., 2020; Liu et al., 2021). As we discussed above, traditionally qualitative, LFAs now exploit advanced detection systems, including smartphone-based imaging, for quantitative measurement of antibiotics (Urusov et al., 2019). In these assays, a nitrocellulose membrane with immobilized biomolecules can use sandwich or competitive formats. Small-molecule antibiotics require a competitive LFA configuration (Calucho et al., 2020; Nuntawong et al., 2022; Sajid et al., 2015). However, conventional competitive LFAs face challenges such as narrow dynamic ranges and dependence on specialized detectors, limiting their utility at clinically relevant concentrations (15–20 mg/L) for vancomycin (Hale et al., 2017). To address these limitations, a novel competitive LFA featuring two test lines, an antibody line and an avidin line has been developed. This dual-line design leverages biotin-avidin interactions to sequester excess conjugate, enabling differential signal analysis. By correlating the intensity of both lines, accurate quantification across a broader dynamic range is achieved. Validation involved a two-phase study: first, the LFA was optimized for vancomycin quantification in serum and benchmarked against ELISA and traditional LFAs; second, spiked serum samples assessed accuracy, reproducibility, specificity, and applicability for TDM (Riezk et al., 2025).

HPLC remains a staple in antibiotic TDM, offering moderate sensitivity, high reproducibility, and compatibility with UV or fluorescence detection. Nonetheless, low-concentration antibiotics such as tigecycline and linezolid necessitate the heightened sensitivity and specificity of LC-MS/MS, which also allows multiplexed analyte detection, establishing it as the gold standard in bioanalysis. Immunoassays like fluorescence polarization immunoassay (FPIA), enzyme-multiplied immunoassay technique (EMIT), and ELISA remain widely used for vancomycin due to rapid processing and integration with hospital laboratory workflows, though they may suffer from cross-reactivity and limited specificity.

FPIA, extensively applied in China and abroad, provides rapid TDM information for vancomycin dose adjustments (Farin et al., 1998; Pfaller et al., 1984). However, no specific TDM system for norvancomycin exists. Currently, norvancomycin concentrations are determined by HPLC or microbiological assays (de Jesús Valle et al., 2008; Najjar et al., 1995). Both approaches present challenges: HPLC is technically demanding, and microbiological assays are time-intensive and susceptible to interference from concomitant medications. This study employed FPIA and HPLC to measure serum norvancomycin in 300 clinical samples, deriving a correlation-based FPIA algorithm subsequently validated in 70 additional samples (Wu et al., 2012).

Blood plasma and serum are rich in diagnostic information but require extensive processing for analysis. Real-time analyte detection is particularly critical for TDM. Surface-enhanced Raman scattering (SERS) represents a highly sensitive, label-free technique with molecular specificity (Bantz et al., 2011; Anker et al., 2008; Anema et al., 2011), adaptable to small molecules, drugs, proteins, nucleic acids, cells, and microorganisms (Kneipp et al., 2010; Cao et al., 2002; Qian et al., 2008). Therapeutic drugs, often containing conjugated rings, exhibit strong Raman scattering, enabling sensitive detection (Taylor et al., 2014). While SERS has been applied to saliva and urine, blood analysis necessitates prior separation to overcome competitive adsorption by proteins and metabolites, which impede analyte access to SERS-active hotspots and reduce assay sensitivity (Schlücker, 2014; Moskovits, 2005; Bonifacio et al., 2014; Stosch et al., 2005).

To overcome these obstacles, a hierarchical zwitterionic surface modification for SERS-based optofluidic systems was developed. The inner layer consists of self-assembled monolayers (SAM) with functional thiols to attract analytes or amplify weak Raman signals, while the outer layer employs zwitterionic poly (carboxybetaine acrylamide) (pCBAA) grafted via surface-initiated atom transfer radical polymerization (SI-ATRP) to resist protein fouling. This configuration enables continuous real-time quantification of doxorubicin in undiluted human plasma, alongside monitoring of other TDM-requiring drugs, fructose, and pH, providing a generalized platform for SERS-based biosensing in complex biological matrices (Sun et al., 2016).

### Population pharmacokinetic analysis for TDM of antibiotics: bayesian model

A previous study (Lin et al., 2021) detailed population pharmacokinetic (PopPK) investigation was performed using 993 serum concentration–time data points obtained from 374 adult patients. Of these, 294 subjects were allocated for model construction and 80 for external validation, both subsets being randomly assigned through *Kinetica* software (Alqahtani et al., 2018; Lin et al., 2021). The analysis employed a nonlinear mixed-effects modeling framework, a robust approach capable of distinguishing inter-individual variability from residual unexplained variance, thereby improving model precision and clinical applicability (Alqahtani et al., 2018; Lin et al., 2021).

Model selection and performance evaluation were guided by maximum likelihood estimation (MLE) metrics, including the log-likelihood (LL), Akaike Information Criterion (AIC), and Bayesian Information Criterion (BIC). Superior model performance was indicated by lower AIC/BIC and higher LL values. In addition, diagnostic plots including conditional weighted residuals, population-predicted *versus* observed concentrations, and residual distributions were visually inspected to confirm homoscedasticity, lack of bias, and appropriate random error dispersion (Lin et al., 2021). External validation was executed using an 80-patient subset through the Bayesian feedback method implemented in *Kinetica*. This approach integrates prior population information with individual patient data to refine parameter estimation and assess predictive adaptability under real-world variability. The mean prediction error (MPE) and mean absolute error (MAE) were calculated as indices of model bias and precision, respectively (Lin et al., 2021). The final PopPK model demonstrated robust predictive capability, confirming its suitability for individualized TDM of antibiotics. The integration of EM-based population modeling with Bayesian feedback enables dynamic, patient-specific dose optimization. This model-informed precision dosing framework enhances clinical decision-making by minimizing underexposure and toxicity risks, particularly for narrow therapeutic index agents such as vancomycin and related glycopeptides (Lin et al., 2021).

### Implications of bioluminescent sensors for effective TDM of antibiotics

Achieving an optimal balance between therapeutic efficacy and toxicity remains a cornerstone of precision pharmacotherapy. For many antibiotics and narrow therapeutic index drugs, TDM provides essential feedback to ensure drug concentrations remain within a safe and effective range (Griss et al., 2014). Traditional TDM methods, however, depend on centralized laboratories equipped with sophisticated analytical systems, imposing logistical delays and limiting real-time dose optimization. The advent of semisynthetic bioluminescent sensors introduces a transformative alternative for near-patient and at-home drug monitoring, offering rapid, accurate, and low-cost quantification directly from minimal blood samples (Griss et al., 2014). These bioluminescent sensors operate on a modular platform that integrates a protein-based recognition element with a synthetic chemical moiety, collectively designed to confer selectivity toward diverse analytes, including immunosuppressants, anticancer drugs, antiepileptics, and antibiotics (Griss et al., 2014). Minimal sample volumes merely a drop of serum or plasma can be applied onto a paper substrate, and luminescent emission is captured using a simple point-and-shoot or smartphone-based imaging system. This simplified, portable configuration has the potential to democratize access to TDM, particularly in resource-limited or decentralized clinical settings, enhancing both treatment safety and patient autonomy (Griss et al., 2014).

A key innovation in this domain is the development of luciferase-based indicators of drugs (LUCIDs), a new class of ratiometric, bioluminescent biosensors engineered to fulfill the stringent requirements of modern point-of-care testing: quantitative accuracy, minimal operator intervention, compatibility with portable devices, and single-drop sample handling (Griss et al., 2014). Structurally, each LUCID consists of three integral components: (i) a receptor protein selective for the target analyte, (ii) a luciferase enzyme acting as the light-emitting source, and (iii) a synthetic molecule harboring both a fluorophore and a ligand capable of binding the receptor protein. When the tethered ligand binds intramolecularly, the fluorophore and luciferase are brought into close proximity, enabling bioluminescent resonance energy transfer (BRET) (Griss et al., 2014). Upon analyte binding, the ligand is displaced, causing a measurable decrease in BRET efficiency. Quantification is achieved by recording the emission ratio of blue light (from the luciferase) to red light (from the fluorophore), providing a self-referenced, concentration-independent signal (Griss et al., 2014).

This approach builds on prior studies demonstrating the feasibility of Förster resonance energy transfer (FRET)-based biosensors (Bru et al., 2009; Brun et al., 2012; Brun et al., 2011; Masharina et al., 2012), yet LUCIDs offer distinct advantages by eliminating dependence on external excitation light sources, enhancing detection sensitivity, and reducing background noise attributes that are particularly valuable for portable and battery-operated diagnostic devices. By integrating bioluminescence-driven quantification into TDM, LUCIDs mark a significant leap toward personalized, real-time pharmacokinetic monitoring (Griss et al., 2014).

Decentralizing diagnostics from the laboratory to the individual represents a paradigm shift akin to the evolution of personal health tracking. Analogous to how wearable technologies and smartphones have revolutionized fitness monitoring, bioluminescent TDM systems promise individualized therapeutic oversight from virtually any setting. While electrochemical biosensors (e.g., glucose meters) remain the predominant point-of-care devices, few quantitative biosensors for other analytes have achieved clinical implementation (Turner, 2013). LUCIDs address this gap by providing a novel, luciferase-based bioluminescent mechanism that enables accurate drug quantification in complex biological matrices using only ambient-level technology. A defining feature of the LUCID design is its adaptability (Griss et al., 2014). By reconfiguring the receptor–ligand pair through protein engineering and rational ligand design, the system can be tailored to detect virtually any small-molecule analyte. Two parameters are critical in this process: (i) the geometry of the binding protein, which governs energy transfer efficiency, and (ii) the binding affinity of the tethered ligand, which determines the sensor’s responsiveness within the therapeutic range (Griss et al., 2014). Rigid polyproline linkers connecting the SNAP-tag and luciferase can minimize background BRET in the sensor’s open state, while optimized attachment points ensure maximal transfer efficiency in the closed state. When neither terminus aligns appropriately, circular permutation strategies can be employed to reposition the termini near the binding pocket, enhancing signal dynamics (Griss et al., 2014) (Table 2).

**TABLE 2:** Emerging bioanalytical strategies for Therapeutic Drug Monitoring (TDM) of critical antibiotics and their role in precision dosing against AMR pathogens.

Novel bioanalytical strategy/Antibiotic	Method/Analytical platform	Implication in TDM	Implications to enhance precision dosage therapy against AMR-resistant bacteria	Refs
Omadacycline (Next-Generation Tetracycline)	LC–MS/MS using acetonitrile protein precipitation and C18 reverse-phase column with 0.1% formic acid mobile phase	Provides high analytical precision and sensitivity for omadacycline quantification in plasma; meets LLOQ and regulatory standards for clinical validation	Enables individualized dose optimization in critically ill patients; minimizes resistance emergence by maintaining target AUC0–24 h/MIC ratios against MDR respiratory pathogens	Riezk et al. (2025), Wang et al. (2025), Yang et al. (2022), Tzanis et al. (2017), Lepak et al. (2017), Rodvold and Pai (2019), Lepak et al. (2019), Durães and Sousa (2019), Yaghoubi et al. (2022), Annoni et al. (2020), Flarakos et al. (2017), Suhang et al. (2024), Hu et al. (2024)
Vancomycin	LC–MS/MS, HPLC, and dual-line competitive LFA with gold nanoparticle conjugation and smartphone quantification	Allows both centralized (LC–MS/MS) and rapid bedside (LFA) monitoring with wide dynamic range (2.88–45,000 ng/mL)	Supports Bayesian model-informed dosing to maintain AUC/MIC ≥400; reduces nephrotoxicity and MRSA resistance by real-time dose correction	Riezk et al. (2025), Revilla et al. (2010), Edwards et al. (2012), Brink (2012), Ibrahim et al. (2000), Alqahtani et al. (2018), Okada et al. (2018), Lin et al. (2021), Rao et al. (2020), Tietjen et al. (2022), Hale et al. (2017)
Linezolid	LC–MS/MS and immunoassay-based quantification integrated with PK/PD modeling	Facilitates TDM-guided optimization of linezolid plasma exposure to reduce hematologic and neurologic toxicities	Enhances efficacy in critically ill patients with renal or hepatic dysfunction, preventing resistance development in VRE and MRSA strains	Rao et al. (2020), Tietjen et al. (2022)
Tigecycline	LC–MS/MS coupled with microextraction; validated for low-concentration antibiotic quantitation	Provides accurate monitoring of tigecycline plasma levels under extracorporeal support and renal replacement	Enables adaptive dosing to sustain AUC/MIC targets and overcome variable clearance in multidrug-resistant Enterobacteriaceae infections	Rao et al. (2020), Tietjen et al. (2022)
Norvancomycin	Fluorescence polarization immunoassay (FPIA) and HPLC correlation algorithm	Offers a faster, less resource-intensive assay compared with microbiological methods for serum level estimation	Facilitates accurate, real-time vancomycin analogue monitoring to optimize therapy and minimize cross-resistance	Farin et al. (1998), Pfaller et al. (1984), de Jesús Valle et al. (2008), Najjar et al. (1995), Wu et al. (2012)
Surface-Enhanced Raman Scattering (SERS)–Based Optofluidic Platform	Hierarchical zwitterionic surface-modified SERS chip with antifouling SAM and poly (carboxybetaine acrylamide) layer	Enables continuous, label-free quantification of small-molecule drugs (e.g., doxorubicin, vancomycin analogues) in undiluted plasma	Establishes a generalizable real-time TDM platform capable of detecting nanomolar drug levels in complex biological matrices to prevent resistance	Bantz et al. (2011), Anker et al. (2008), Anema et al. (2011), Kneipp et al. (2010), Cao et al. (2002), Qian et al. (2008), Taylor et al. (2014), Schlücker (2014), Moskovits (2005), Bonifacio et al. (2014), Stosch et al. (2005), Sun et al. (2016)
Population Pharmacokinetic (PopPK) Model–Bayesian Framework	Nonlinear mixed-effects modelling using large serum datasets and Bayesian feedback validation	Allows individualized dose prediction by integrating population PK parameters with patient-specific data	Reduces underexposure and toxicity for narrow therapeutic index antibiotics such as vancomycin; supports precision antimicrobial stewardship	Ibrahim et al. (2000), Alqahtani et al. (2018), Lin et al. (2021)
Luciferase-Based Bioluminescent Biosensors (LUCIDs)	Protein–luciferase hybrid sensors employing bioluminescent resonance energy transfer (BRET)	Achieves portable, rapid, and quantitative antibiotic TDM from single-drop samples using smartphone imaging	Empowers near-patient and at-home antibiotic monitoring; improves treatment adherence and adaptive dosing in resource-limited settings	Griss et al. (2014), Bru et al. (2009), Brun et al. (2012), Brun et al. (2011), Masharina et al. (2012)

The sensor’s functionality is governed by the equilibrium between its open and closed states, a process defined by the relative affinities of the tethered ligand and free analyte (Krishnamurthy et al., 2007). Empirical studies estimate an effective molarity of approximately 100 μM, implying that a tethered ligand with an affinity below 10 µM will favor the closed (high BRET) configuration under analyte-free conditions. The concentration–response (c_50_) of the sensor thus becomes tunable by adjusting ligand affinity to align with clinically relevant drug levels. Unlike intermolecular assays, this intramolecular displacement mechanism confers higher precision, faster response kinetics, and improved reproducibility.

LUCIDs represent the first generation of BRET-based biosensors capable of quantifying small molecules with substantial ratio metric change, surpassing the signal dynamics of previously reported indicators (Saito et al., 2010). This advancement establishes a foundation for next-generation diagnostics that could integrate microfluidic channels, multiplexed analyte detection, and automated data processing for real-time decision support in antimicrobial stewardship and dose optimization. Future prototypes envision a fully self-contained paper-based assay, wherein the luciferase substrate and sensor proteins are immobilized on layered membranes. Upon application of a blood drop, serum components would diffuse through a red blood cell–filtering layer, activating the luminescent reaction. A handheld photometric reader or even a smartphone camera could then measure the emitted blue and red-light intensities, automatically computing the analyte concentration via onboard algorithms. This system would require no specialized training, making it equally suitable for clinicians and patients. Stability studies suggest that dried protein reagents and luciferase substrates can retain activity for extended periods, enabling long-term storage and transportation under ambient conditions (Pollock et al., 2012). With continued optimization, LUCIDs could become a universal bioluminescent TDM platform, providing accessible, rapid, and cost-effective therapeutic monitoring across global healthcare infrastructures. By bridging the gap between synthetic biology, optics, and clinical pharmacology, these biosensors hold the potential to redefine precision dosing paradigms and extend the reach of personalized medicine (Griss et al., 2014).

### Minimally invasive microneedle-based TDM for antibiotics

A recent investigation (Ranamukhaarachchi et al., 2016a) introduced a painless and minimally invasive alternative for TDM through the development of hollow microneedle systems capable of extracting *ultra-trace* volumes (<1 nL) of interstitial fluid (ISF) for drug quantification. Unlike conventional venipuncture-based TDM methods, this innovative system utilizes a microneedle lumen functionalized as an *in situ* microreactor, which enables simultaneous analyte capture and binding during sample collection, eliminating the need for additional transfer steps (Ranamukhaarachchi et al., 2016a). The extracted analytes are immediately processed within an integrated optofluidic biosensing platform, which employs a high-sensitivity optical absorbance detection scheme for rapid and quantitative analysis (Ranamukhaarachchi et al., 2016a). In conventional assays, vancomycin, a glycopeptide antibiotic requires sample volumes of 50–100 μL and exhibits a LoD in micromoles (μM) (Ranamukhaarachchi et al., 2016a). In contrast, the newly proposed microneedle-optofluidic biosensor achieves a substantial analytical enhancement, detecting vancomycin at concentrations below nmol nM of sample. This unprecedented sensitivity demonstrates the system’s capacity for real-time, bedside monitoring, potentially reducing patient discomfort, hospitalization duration, and healthcare expenditure (Ranamukhaarachchi et al., 2016a).

Vancomycin serves as an exemplary candidate for interstitial fluid-based TDM, owing to the strong pharmacokinetic correlation between interstitial fluid and plasma concentrations (Kiang et al., 2012). Clinically, vancomycin (VANC) is employed as a last-line therapeutic agent against MRSA infections (Jarvis, 1998; Loll et al., 1999). It is typically administered intravenously, maintaining therapeutic peak levels between 20 and 40 μg/mL and trough concentrations of 3–10 μg/mL (Kiang et al., 2012; Jarvis, 1998; Loll et al., 1999; Hierholzer et al., 1995). However, exceeding this therapeutic window is associated with nephrotoxicity and irreversible ototoxicity (Hierholzer et al., 1995). To mitigate these risks, several commercial immunoassay kits are available, including VANC Flex® (Siemens Healthcare Diagnostics, United Kingdom), QMS® Vancomycin (Thermo Fisher Scientific, United States), and Emit® 2000 Vancomycin (Beckman Coulter, United States). These kits, while analytically robust, necessitate multiple invasive blood draws (>1 mL) per day and large serum volumes (50–100 μL), thereby increasing patient burden, procedural costs, and the risk of iatrogenic complications. Consequently, self-administered point-of-care TDM systems would represent a transformative advancement in precision antibiotic dosing.

Microneedle technology offers a promising avenue toward this goal. Microneedles, typically sub-millimetre in dimension, were originally designed for transdermal drug delivery (Mansoor et al., 2013; Sivamani et al., 2005; Stoeber and Liepmann, 2005; Mansoor et al., 2015; Prausnitz and Langer, 2008). By penetrating the stratum corneum, the skin’s primary diffusion barrier, they enable direct access to the viable epidermis and dermis layers (Ranamukhaarachchi et al., 2016b). Importantly, hollow microneedles can also function in reverse, serving as micro-sampling devices for the extraction of blood (Li et al., 2013) or interstitial fluid (Mukerjee et al., 2004). Interstitial fluid, which bathes all tissue cells, is a biochemically rich yet particulate-free matrix, containing markedly fewer proteins, 5 to 10 times less protein than serum, making it ideal for biosensing. However, the limited ISF volume within the skin (epidermis: ∼20 nL/mm^2^; dermis: ∼800 nL/mm^2^) (Groenendaal et al., 2010) presents a significant challenge for efficient extraction. Previous studies have achieved ISF sampling up to 200 nL per microneedle via capillary action over 15–20 min (Mukerjee et al., 2004) or 1–10 μL using vacuum-assisted glass microneedles under negative pressures of 27–67 kPa for 2–10 min (Wang et al., 2005).

Recent advances in lab-on-a-chip architecture have integrated hollow microneedles for ISF-driven biosensing (Mukerjee et al., 2004; Strambini et al., 2015). However, most designs still rely on post-extraction ISF transfer, which introduces delays, signal loss, and volume constraints. To overcome these limitations, the present study reports a unified microneedle-optofluidic system that performs dual functions: (i) passive ISF collection (sub-nanoliter scale) via capillary forces, and (ii) real-time biochemical reaction and quantification of VAN within the same microneedle lumen (Ranamukhaarachchi et al., 2016b). The inner lumen operates as a bioreactive domain where VAN binding occurs concurrently with sampling, followed by optical signal transduction through a microfluidic channel that introduces reagents for detection. This configuration enables ultra-sensitive quantification within therapeutic ranges, even at picoliter-scale sample inputs, and offers an elegant solution for continuous, painless, and patient-friendly TDM applications in infectious disease management (Ranamukhaarachchi et al., 2016a).

### Novel two-dimensional temperature-responsive chromatography system using dual PNIPAAm-modified columns for safe and efficient TDM

Various analytical platforms, including immunoassay-based and chromatographic techniques, have been employed for TDM (Koch-Weser, 1972; Gross, 1998; Ates et al., 2020; Kumps, 1982). Among these, liquid chromatography remains the most versatile and selective approach for serum drug analysis, as it circumvents the need for specific ligand interactions (Wong, 1989). However, conventional liquid chromatography methods pose practical challenges in clinical environments. They frequently rely on organic solvent-based mobile phases to fine-tune analyte retention, exposing operators to volatile solvents that are unsuitable for point-of-care or hospital laboratories. Moreover, pre-analytical sample pretreatment, involving protein precipitation or denaturation with organic solvents, is labor-intensive and time-consuming, thus delaying timely therapeutic decisions. To address these limitations, a temperature-responsive, two-dimensional chromatography system was developed using dual poly (N-isopropylacrylamide) (PNIPAAm)-modified bead-packed columns. This approach eliminates the need for organic solvents and extensive sample pretreatment, enabling a rapid, safe, and fully aqueous TDM platform. PNIPAAm-based temperature-responsive chromatography harnesses the unique phase-transition properties of this polymer to modulate analyte retention by simply altering column temperature (Kanazawa et al., 1996; Nagase and Okano, 2016; Nagase and Kanazawa, 2020; Nagase et al., 2022a).

**FIGURE 3:**
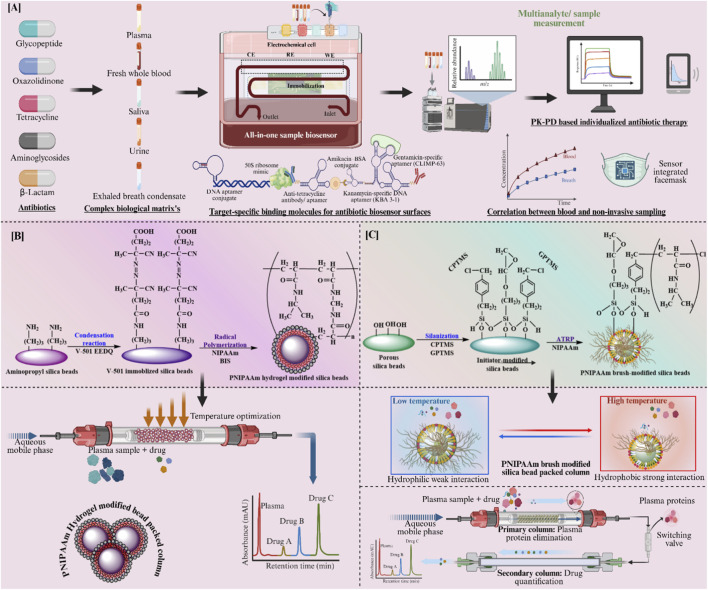
Emerging hybrid analytical and biosensing platforms for multi-analyte antibiotic quantification and PK–PD correlation. This multi-panel figure presents novel analytical architectures designed for next-generation therapeutic drug monitoring (TDM). **(A)** Depicts the integration of multi-matrix sampling (plasma, saliva, urine, breath condensate) into biosensor-based antibiotic detection, employing aptamer-antibody conjugates for specific binding and real-time quantification using electrochemical or mass spectrometric readouts. **(B)** Demonstrates the fabrication and application of PNIPAAm hydrogel-modified silica beads for temperature-responsive chromatography. Radical polymerization and functional surface modification enable selective analyte retention and release based on hydrophilic–hydrophobic phase transitions. **(C)** Illustrates a two-dimensional, dual-column chromatographic system combining protein elimination and drug quantification columns for high-throughput, precise TDM. This hybrid thermoresponsive technology enhances separation efficiency and analytical stability for β-lactam, oxazolidinone, tetracycline, and aminoglycoside antibiotics.

Thermoresponsive polymers such as poly(N-alkyl acrylamide)s, poly(alkyloxide) copolymers, and poly(2-alkyl-2-oxazoline)s have been explored for similar applications (Roy et al., 2013), yet PNIPAAm remains the benchmark material owing to its sharp and reversible phase transition near physiological temperature (32 °C) (Halperin et al., 2015; Nagase, 2021; Nagase et al., 2018a). This polymer undergoes hydration–dehydration transitions, switching between hydrophilic and hydrophobic states across its lower critical solution temperature (LCST) (Halperin et al., 2015; Nagase, 2021; Nagase et al., 2018a; Nagase et al., 2015). Structural fluctuations within PNIPAAm chains swelling at low temperatures and contraction above LCST have been widely exploited across diverse biomedical domains, including thermally modulated drug and gene delivery systems (Nakayama and Okano, 2011; Akimoto et al., 2014; Kono, 2001; Nagase et al., 2019; Maekawa-Matsuura et al., 2019; Nemoto et al., 2019), biosensing and imaging platforms (Mori et al., 2001; Mori and Maeda, 2004; Miyamoto et al., 2007; Tang et al., 2018; Matsuura et al., 2018; Malmstadt et al., 2003), chemically actuated nanodevices (Masuda et al., 2015a; Masuda et al., 2015b; Homm et al., 2017), cell separation technologies (Nagase et al., 2012; Nagase et al., 2017a; Nagase et al., 2017b; Nagase et al., 2020), and temperature-controlled cell culture matrices for tissue engineering (Nagase et al., 2022a; Yamada et al., 1990; Yamato et al., 2007; Nagase et al., 2009; Matsuura et al., 2014; Nagase et al., 2018b; Nagase et al., 2017c; Nakao et al., 2019). Therefore, Figure 2 presents emerging hybrid analytical technologies designed to revolutionize antibiotic TDM through multi-analyte detection and real-time monitoring. Novel biosensors incorporating aptamer and antibody interfaces allow specific antibiotic detection across complex biological matrices such as plasma, saliva, urine, and exhaled breath. Temperature-responsive chromatographic systems using PNIPAAm-modified silica beads offer dynamic analyte separation and efficient protein elimination for enhanced quantification accuracy. The integration of electrochemical detection, mass spectrometry, and microfluidic platforms enables seamless PK–PD correlation. These innovations improve analytical throughput, reduce sample processing time, and support decentralized TDM implementation at the point of care (Figure 3).

Notably, PNIPAAm brush-type stationary phases synthesized via atom transfer radical polymerization exhibit superior performance, offering high uniformity, controlled surface architecture, and predictable analyte interaction profiles (Nagase et al., 2022a; Nagase et al., 2008). Analyte immobilized onto a chromatographic stationary phase, PNIPAAm’stunable hydrophobicity allows temperature-dependent modulation of analyte–polymer interactions, thereby enabling analyte separation using entirely aqueous mobile phases. The retention behavior and column performance depend on the surface graft density and molecular chain length of PNIPAAm immobilized on the stationary matrix (Nagase and Kanazawa, 2020). A two-dimensional (2D) temperature-responsive chromatography platform, consisting of two PNIPAAm brush-modified bead-packed columns connected via a switching valve and in this configuration, the primary column performs serum pretreatment and initial analyte extraction, while the secondary column conducts precise quantitative separation and detection (Majors, 1980; Bushey and Jorgenson, 1990). PNIPAAm brush-modified silica beads were synthesized via atom transfer radical polymerization and used as packing materials for both columns. The dual-column system demonstrated effective separation of protein-bound and free drug fractions, thereby validating its suitability for clinical TDM applications (Nagase et al., 2022a).

The temperature-dependent elution profiles of model drugs revealed sharp and reproducible peaks at 30 °C–40 °C, consistent with PNIPAAm’s volume phase transition behavior. At elevated temperatures, polymer contraction reduced analyte diffusion into the PNIPAAm hydrogel matrix, thereby enhancing elution efficiency (Nagase et al., 2022a). Using this all-aqueous mobile phase, drugs were successfully separated from serum proteins, and concentrations were quantified through calibration-based correlation, confirming the analytical reproducibility and robustness of the system. Importantly, this process circumvented the use of organic solvents entirely, aligning with hospital safety standards and reducing both cost and environmental impact (Nagase et al., 2022a). Further refinement was achieved using a thermoresponsive cationic copolymer, *poly(N-isopropylacrylamide-co–n-butyl methacrylate-co–N,N-dimethylaminopropyl acrylamide)* [P(NIPAAm-co-BMA-co-DMAPAAm)], which was immobilized on silica beads to enhance selectivity for charged analytes. Structural characterization confirmed the successful incorporation of cationic functionalities, providing tunable electrostatic interactions complementary to PNIPAAm’s hydrophobicity. Optimized chromatographic conditions identified 30 °C as the ideal operational temperature, ensuring efficient analyte recovery and minimal matrix interference (Nagase et al., 2022a).

Evaluation using a panel of 13 clinically relevant drugs, including voriconazole, demonstrated reliable quantification in human serum using an all-aqueous mobile phase at physiologically compatible pH values (Nagase et al., 2022a). The system’s performance describes its feasibility as a green analytical technology suitable for on-site, real-time therapeutic drug monitoring, consistent with the growing demand for solvent-free, patient-safe analytical methodologies (Nagase et al., 2022a; Nagase et al., 2007; Nagase et al., 2022b). Collectively, these findings highlight that dual PNIPAAm-based two-dimensional temperature-responsive chromatography provides a novel, solvent-free, and automated solution for clinical TDM. This innovation combines the principles of polymer thermodynamics and analytical chemistry to achieve rapid, reproducible, and environmentally sustainable quantification of drug concentrations directly from serum matrices paving the way for next-generation hospital-integrated monitoring systems (Nagase et al., 2022a; Nagase et al., 2022b).

### Need for the identification and characterization of critical values in TDM and AI implications

TDM represents a cornerstone of precision pharmacotherapy, allowing clinicians to tailor drug dosing to individual PK/PD characteristics (Xiao et al., 2024; Hagel et al., 2019). It encompasses a broad spectrum of drug classes, such as antibiotics, cardioactive agents, immunosuppressants, antiepileptics, and psychotropic medications (Kang and Lee, 2009; Fang et al., 2024). Among these, vancomycin, a glycopeptide antibiotic remains indispensable for treating Gram-positive bacterial infections, particularly those caused by methicillin-resistant *Staphylococcus aureus* (Rybak, 2006; Kalil et al., 2014). When serum drug concentrations exceed a defined critical threshold level that may induce severe or life-threatening toxicity immediate clinical evaluation and intervention become imperative. Timely recognition and management of such critical values in TDM are vital to prevent iatrogenic harm and ensure therapeutic success (Bidu et al., 2020). Laboratory professionals play an instrumental role in promptly communicating critical results to attending physicians, thus bridging analytical data with clinical decision-making (Li et al., 2020). Although reporting systems for critical values are well-established in fields such as clinical chemistry, hematology, coagulation, and microbiology (Li et al., 2020; Pai et al., 2011) comprehensive frameworks for TDM-specific critical values remain underexplored. Most existing TDM studies are drug-centric rather than systemic, limiting insights into cross-category patterns of critical thresholds.

A previously study (Xiao et al., 2024) retrospectively analyzed TDM critical values to delineate their distribution and clinical associations across three key therapeutic agents such as vancomycin, digoxin, and tacrolimus (Xiao et al., 2024). Drugs selected for TDM generally share key pharmacological characteristics, including a quantifiable relationship between plasma concentration and therapeutic effect, narrow therapeutic indices, substantial interindividual variability, prolonged treatment duration, and potential for severe toxicity upon overdose (Ates et al., 2020). Ensuring analytical integrity and prompt interpretation of critical values is paramount, especially in critically ill patients where overdose events may rapidly translate into life-threatening toxicities (Sergi, 2018; Coetzee et al., 2020).

Notably, the prevalence of laboratory critical values exhibits significant heterogeneity among different analytes. A previous report reported 601 (0.83%) critical values among electrolyte and glucose measurements in 72,259 clinical chemistry samples (Niu et al., 2013). Another study assessed hematology, chemistry, coagulation, and microbiology parameters, identifying a 0.49% (38,020/7,706,962) incidence, with the majority (63%) originating from inpatients (Li et al., 2020). However, systematic data on TDM-related critical values remain scarce for effective antibiotics (Xiao et al., 2024).

A national survey from China indicated that vancomycin, valproic acid, carbamazepine, phenytoin sodium, and methotrexate are the most frequently monitored drugs for TDM (Yin et al., 2023). Despite its clinical utility, the role of vancomycin trough concentration remains debated (Xiao et al., 2024). Recent pharmacometric evidence indicates that AUC provides a more accurate index of cumulative drug exposure and correlates more strongly with therapeutic efficacy and nephrotoxicity risk. Consequently, while American and Japanese guidelines now endorse AUC-guided monitoring exclusively, Chinese and European recommendations still include both AUC and trough levels (Matsumoto et al., 2022). Nevertheless, in resource-limited or critically ill settings, trough concentrations continue to serve as pragmatic surrogates for AUC, particularly in non-dialysis patients (Yu et al., 2023). Similarly, tacrolimus TDM predominantly relies on trough monitoring in most transplant centers, though the superiority of AUC-guided strategies warrants prospective validation (Brunet et al., 2019).

Laboratory turnaround time (TAT) represents a critical determinant in the efficiency of TDM-based critical value reporting. In a closed-loop notification system for non-TDM parameters, median total and reporting times were 393 and 41 min, respectively (Li et al., 2020). Median TAT for TDM critical values ranged from 186 to 251 min, with reporting times between 108 and 170 min. Delays largely stemmed from multi-campus logistics and centralized laboratory operations. To mitigate such delays, decentralization of analytical workflows via on-site TDM laboratories or AI-integrated biosensing platforms can substantially enhance responsiveness (Xiao et al., 2024; Goswami et al., 2010; Chauhan et al., 2014).

Currently, analytical methodologies for TDM encompass immunoassays, chromatographic separations, biosensors, electrochemical detection, capillary electrophoresis, and microbial assays (Ates et al., 2020; Fang et al., 2024; Wicha et al., 2021). Although LC–MS offers superior selectivity and sensitivity, its routine adoption remains constrained by cost, technical expertise, and infrastructural demands (Wicha et al., 2021). Emerging biosensor-based TDM platforms integrating microfluidics, nanomaterials, and artificial intelligence (AI)-driven analytics represent a promising frontier for real-time, bedside monitoring of therapeutic agents potentially transforming critical value detection and reporting paradigms (Xiao et al., 2024). Future multicentric, prospective investigations integrating pharmacogenetics, AI-assisted prediction models, and real-world clinical outcomes are warranted to elucidate the dynamic evolution of critical drug levels. In summary, TDM continues to be an indispensable component of individualized pharmacotherapy, enabling precise dose optimization to maintain a balance between efficacy and toxicity (Xiao et al., 2024). The identification and systematic characterization of TDM critical values hold immense potential to improve patient safety. AI-supported analytics can revolutionize the management of critical values in modern precision medicine (Xiao et al., 2024).

### Integration of artificial intelligence into predictive TDM frameworks

AI/ML technologies are rapidly transforming the landscape of therapeutic drug monitoring by enabling predictive modeling, individualized dosing algorithms, and automated critical value surveillance. AI-driven pharmacometric models can integrate multidimensional data including pharmacokinetics, pharmacodynamics, genomics, metabolomics, and clinical parameters to forecast drug concentration-time profiles with remarkable precision. Deep learning architectures, such as recurrent neural networks (RNNs) and transformer-based models, can continuously learn from real-world hospital data streams to anticipate impending toxic or subtherapeutic concentrations even before laboratory confirmation (Arnold et al., 2025). These models can flag potential critical values, recommend dosage adjustments, and trigger early clinical alerts, thus shortening the latency between laboratory detection and clinical action (Arnold et al., 2025).

Moreover, the incorporation of pharmacogenomic data such as polymorphisms in drug-metabolizing enzymes (e.g., *CYP450*, *CYP3A5*, *UGT1A9*) and transporters (*ABCB1*, *SLCO1B1*) can enhance AI algorithms’ ability to predict interindividual differences in drug exposure and toxicity risk. By coupling genomic data with Bayesian forecasting, AI-assisted TDM systems can provide real-time, adaptive dose recommendations that evolve with patient-specific factors such as organ dysfunction, drug-drug interactions, or inflammatory biomarker levels (Arnold et al., 2025).

The emerging paradigm of closed-loop TDM, powered by AI and biosensor technologies, envisions a system where patient samples are analyzed at the point of care, data are automatically interpreted by predictive algorithms, and optimized dosing suggestions are relayed to clinicians in real time (Arnold et al., 2025). Such integration not only reduces turnaround time but also minimizes human error in data interpretation. In the near future, hybrid models combining electronic health records, laboratory data, and wearable biosensing inputs could autonomously identify patients at risk of crossing critical thresholds, initiating proactive interventions before clinical deterioration occurs (Arnold et al., 2025).

In essence, the convergence of TDM with AI-enabled analytics heralds a shift from reactive to predictive pharmacotherapy, transforming critical value management into an anticipatory, precision-guided process that ensures optimal drug efficacy, minimizes toxicity, and enhances overall patient safety in both inpatient and outpatient settings (Arnold et al., 2025).

### Machine learning and artificial intelligence in AMR prediction and TDM optimization

ML and AI have emerged as transformative tools for antimicrobial resistance prediction and personalized TDM, owing to their ability to integrate complex clinical, microbiological, and genomic datasets (Arnold et al., 2025). The ML-based AMR predictions, numerous investigations predominantly conducted in high-resource healthcare systems have utilized multifaceted data sources, including demographic characteristics, prior antibiotic exposures, historical antimicrobial susceptibility testing results (Arnold et al., 2025), and microbiome profiles to forecast drug resistance in infected patients and optimize antibiotic dosage at both national and international levels of surveillance and intervention programs (Liu et al., 2017). By incorporating next-generation sequencing data such as whole genome sequencing (WGS), metagenomics, and transcriptomics, AI/ML approaches can discern the presence of resistance-conferring genes, single-nucleotide polymorphisms, and transcriptomic signatures associated with altered drug sensitivity. These computational frameworks have been instrumental in elucidating resistance evolution and supporting precision-based antimicrobial therapy design (Arnold et al., 2025).

With the continuing decline in sequencing costs and the exponential rise in publicly available genomic datasets, model interpretability and predictive resolution for AMR are rapidly advancing (Evans et al., 2021). Predictive ML models provide a powerful approach for extracting latent patterns from large-scale surveillance data, employing analytical modalities such as temporal trend modeling, network topology analysis, and phylogenetic inference to track the dynamics of resistance gene dissemination. These methods can detect rare phenotypic behaviors, micro-epidemic clusters, and overexpression of antibiotic-resistance determinants, key elements for early containment of resistance spread in hospital and community settings (Arnold et al., 2025; Vandenberg et al., 2020).

The diagnostic utility of matrix-assisted laser desorption/ionization time-of-flight mass spectrometry (MALDI-TOF MS) remains contingent upon the comprehensiveness of reference spectral databases, which often lack adequate representation of rare or novel microbial taxa (Arnold et al., 2025; Ates et al., 2024). These limitations stem primarily from restricted data coverage rather than the technological methodology itself. AI-driven solutions, however, can enhance MALDI-TOF performance by automating spectral pattern recognition and database enrichment, allowing for rapid, objective identification of resistant pathogens. By reducing the dependence on manual interpretation, AI integration into diagnostic microbiology can drastically shorten turnaround times, streamline workflows, and improve diagnostic precision compared to conventional culture-based methods (Arnold et al., 2025; Ates et al., 2024).

Intravenous antibiotic administration particularly with β-lactam antibiotics constitutes the cornerstone of sepsis management (Williams et al., 2023). Prompt initiation of antimicrobial therapy, ideally within 1 hour of diagnosis and following appropriate specimen collection, is critical to improving patient survival (Hagel et al., 2022). However, the heterogeneity of sepsis presentation frequently impedes early detection, leading to therapeutic delays and subsequent organ dysfunction. Critically ill patients often exhibit profound alterations in pharmacokinetics due to sepsis-induced pathophysiology and intensive care interventions (Jager et al., 2016; Adnan et al., 2013; Roberts et al., 2010), resulting in highly variable β-lactam exposures (Roberts et al., 2014a). The increased antibiotic pressure in ICUs further promotes the emergence of less susceptible pathogens, complicating dose optimization and attainment of pharmacodynamic targets (Gonçalves-Pereira and Póvoa, 2011).

TDM offers a precision-based strategy to maintain plasma antibiotic concentrations within target therapeutic windows, potentially mitigating treatment failure and toxicity. Nevertheless, several logistical and operational barriers, including cost, turnaround time, and analytical availability, limit the widespread implementation of β-lactam TDM in clinical settings (Ates et al., 2020). Consequently, clinical prioritization strategies must identify patient subgroups most likely to derive measurable benefits (Legg et al., 2023). Despite multiple randomized controlled trials assessing β-lactam TDM in ICUs (Hagel et al., 2019; Abdulla et al., 2020; De Waele et al., 2014; Sime et al., 2015), Evidence remains inconclusive regarding its direct impact on patient survival. Therefore, holistic evaluation frameworks encompassing clinical cure, microbiological eradication, resistance emergence, and cost-effectiveness are necessary for meaningful outcome interpretation (Ates et al., 2022; Ates et al., 2024; Roberts et al., 2010; Gonçalves-Pereira and Póvoa, 2011).

Traditional statistical methods struggle to process the multidimensional, non-linear, and heterogeneous data typical of ICU cohorts. ML techniques offer a compelling alternative by learning complex, dynamic associations from large temporal datasets (Arnold et al., 2025). By integrating clinical, biochemical, and PK/PD information, ML algorithms can generate a latent multidimensional “patient state” representation that captures disease progression and treatment response in real time. This data-driven representation allows differentiation between stable and deteriorating states, facilitating continuous monitoring of patient trajectories and quantifying therapeutic response patterns. Such analyses provide novel mechanistic insights into the dynamic interplay among antibiotic exposure, immune response, and recovery outcomes.

ML methodologies to quantify the influence of TDM on patient recovery dynamics, focusing on three interlinked domains: (1) modelling patient state trajectories during piperacillin/tazobactam therapy, (2) evaluating the impact of TDM-guided dose adjustments on patient recovery rates, and (3) determining the association between TDM implementation and patient survival. A randomized controlled trial data (DRKS00011159), demonstrates that ML algorithms can effectively identify high-dimensional clinical features that distinguish between healthy and critical states. These models hold potential as computational biomarkers for disease severity stratification and as a foundation for developing a continuous, data-derived “multidimensional SOFA score.” The integration of ML within TDM thus bridges the gap between empirical therapy and individualized precision medicine, transforming static drug monitoring into a dynamic, adaptive clinical tool.

Recent advancements further conclude the superior predictive capability of ML over traditional (PopPK) models (Arnold et al., 2025; Li QY. et al., 2024). As described previously (Arnold et al., 2025; Li QY. et al., 2024), Most ML frameworks achieved lower prediction errors and enhanced accuracy compared with PopPK-based predictions. Deep learning models, such as those developed by Nigo et al., outperformed Bayesian algorithms in predicting vancomycin trough and random concentrations by incorporating broader, nonlinear patient-specific variables (Nigo et al., 2022). Similarly, the ML model developed by Woillard et al. (Woillard et al., 2021), for tacrolimus AUC estimation using limited concentration data surpassed the conventional maximum *a posteriori* Bayesian estimation (MAP-BE) approach, highlighting the potential of ML to reduce sampling frequency and resource use. Moreover, ML-driven covariate analyses have yielded results comparable to PopPK models but with markedly improved computational efficiency (Sibieude et al., 2021).

From a methodological perspective, ML algorithms can be classified into supervised, unsupervised, and reinforcement learning categories based on the nature of input data and training strategies (Eckardt et al., 2021). Supervised learning relies on labeled datasets to predict or classify outcomes, while unsupervised learning uncovers hidden structures within unlabeled datasets, facilitating hypothesis generation and pattern discovery. Reinforcement learning, through iterative trial-error feedback, is particularly suited for modeling optimal dynamic dosing strategies in adaptive clinical environments. However, real-world data rarely produce a single robustly supervised model capable of universal generalization. To overcome this limitation, ensemble learning frameworks such as boosting, bagging, and stacking combine multiple weakly supervised models into a stronger, unified predictor. Among studies examined, ensemble models constituted over half of all final ML algorithms (N = 33, 51.6%), with boosting-based architectures (e.g., XGBoost, LightGBM, CatBoost) being most prevalent (66.7%), followed by bagging (Random Forest; 21.2%) and stacking (12.1%). Additionally, 26.6% of studies incorporated neural network architectures, emphasizing the growing utility of deep learning paradigms in clinical pharmacology (Li QY. et al., 2024).

The analytical performance of MALDI-TOF MS in AMR diagnostics remains inherently dependent on the diversity of its spectral reference databases, which often lack sufficient representation of rare or emerging pathogens. Such limitations primarily arise from sampling bias and incomplete dataset acquisition, rather than shortcomings in analytical technology (Arnold et al., 2025; Ates et al., 2024; Li QY. et al., 2024). AI-driven augmentation of diagnostic workflows has thus become indispensable, accelerating spectrum interpretation, automating pattern recognition, and reducing manual labor in microbiological testing (Arnold et al., 2025; Ates et al., 2024; Li QY. et al., 2024). These algorithmic efficiencies not only shorten diagnostic turnaround times but also enhance reproducibility and throughput compared to conventional phenotypic methods, offering a paradigm shift in routine antimicrobial diagnostics (Arnold et al., 2025; Ates et al., 2024; Li QY. et al., 2024).

For instance, the intravenous antibiotic therapy, particularly involving β-lactams, represents a cornerstone of sepsis management (Williams et al., 2023). Timely antibiotic administration, ideally within the first hour following diagnostic suspicion and culture acquisition, is associated with improved survival outcomes (Hagel et al., 2022). Nonetheless, the inherently heterogeneous presentation of sepsis often delays recognition and treatment initiation, elevating risks of multiorgan failure and adverse outcomes. Critically ill patients in ICUs frequently experience altered pharmacokinetics due to dynamic pathophysiological states, volume shifts, and organ dysfunction (Jager et al., 2016; Adnan et al., 2013; Roberts et al., 2010), leading to unpredictable β-lactam exposures (Roberts et al., 2010). As ICU environments are hotspots for antimicrobial use, selective pressure often results in isolates exhibiting decreased susceptibility, complicating attainment of optimal pharmacodynamic targets (Gonçalves-Pereira and Póvoa, 2011).

**FIGURE 4:**
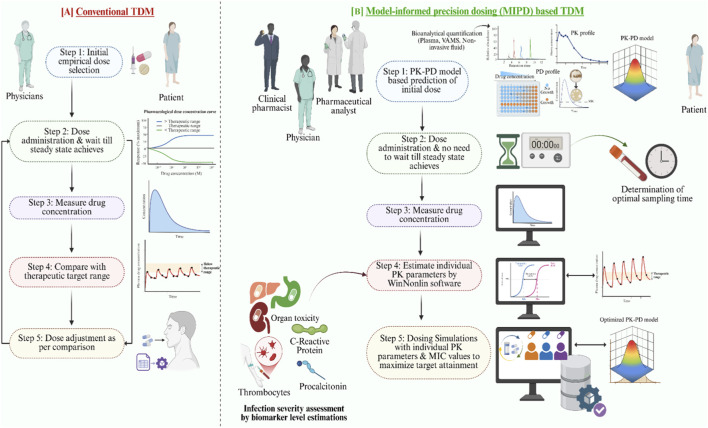
Comparative framework of conventional and model-informed precision dosing (MIPD) for therapeutic drug monitoring (TDM). This figure contrasts traditional empirical TDM approaches with advanced model-informed precision dosing for individualized antibiotic optimization. **(A)** Conventional TDM follows a sequential cycle of empirical dose selection, steady-state sampling, plasma concentration measurement, and delayed dose adjustment based on therapeutic targets. This process is time-intensive and reactive. **(B)** In contrast, the MIPD approach integrates pharmacokinetic/pharmacodynamic (PK/PD) modeling, real-time bioanalytical quantification (via LC-MS/MS, volumetric absorptive microsampling, or non-invasive matrices), and simulation-based dose prediction. Parameters are refined using software platforms such as WinNonlin, enabling early optimization without steady-state waiting. This integrative workflow allows continuous feedback between biomarker estimation (e.g., C-reactive protein, procalcitonin) and infection severity, ensuring maximized target attainment and minimized toxicity in critically ill patients.

**FIGURE 5:**
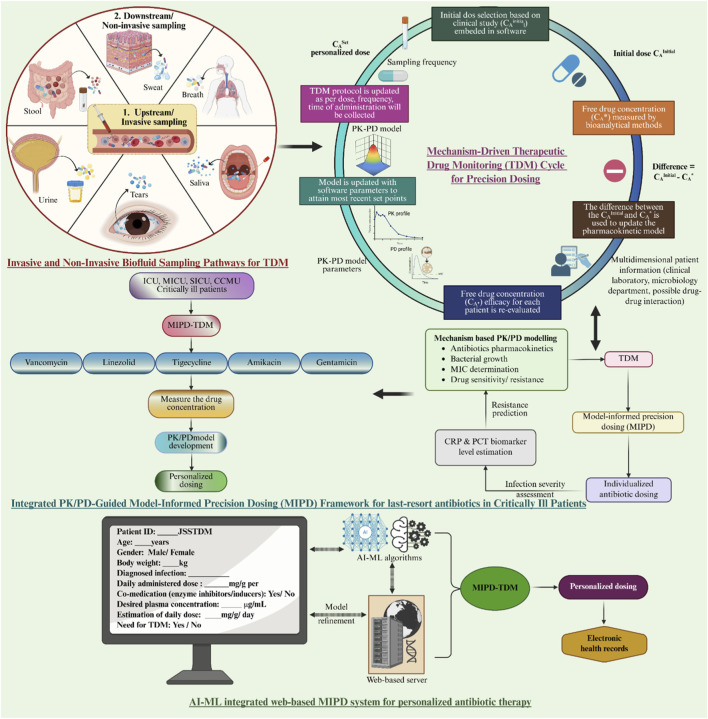
Integrated model-informed precision dosing (MIPD)–machine learning (ML) framework for individualized antibiotic therapy. This integrative schematic delineates a next-generation precision TDM ecosystem linking invasive and non-invasive biofluid sampling, pharmacokinetic modeling, and artificial intelligence (AI)-driven decision support. Upstream sampling (plasma, urine) and downstream non-invasive fluids (sweat, saliva, tears, breath) provide real-time bioanalytical inputs for PK-PD modeling cycles. Mechanism-driven TDM updates dosing protocols by dynamically correlating measured and predicted drug concentrations (C_meas vs. C_pred) to refine model accuracy. The MIPD–TDM module interfaces with AI/ML-based clinical databases for individualized dosing simulations of vancomycin, linezolid, tigecycline, amikacin, and gentamicin. Integration with electronic health records supports adaptive feedback for infection severity assessment and therapeutic optimization. This system represents a transformative shift toward personalized, data-driven antimicrobial stewardship.

TDM offers a precision-based strategy to individualize dosing and ensure sustained antibiotic concentrations within therapeutic windows, mitigating risks of both underexposure and toxicity. However, widespread clinical implementation of TDM of β-lactam/vancomycin/linezolid/tigecycline faces logistical, financial, and operational barriers, including assay unavailability, delayed result reporting, and resource constraints (Ates et al., 2020). Consequently, prioritization of patient subsets most likely to benefit from TDM becomes essential (Legg et al., 2023). Although multiple randomized controlled trials have assessed the impact of β-lactam TDM in ICU settings (Hagel et al., 2019; Hagel et al., 2022; Abdulla et al., 2020; De Waele et al., 2014; Sime et al., 2015), definitive improvements in mortality or clinical cure rates remain elusive for vancomycin/linezolid/tigecycline. Addressing this evidence gap requires integrating multidimensional outcome measures encompassing microbiological eradication, resistance emergence, morbidity, mortality, and cost-effectiveness to construct a holistic framework for optimizing β-lactam/vancomycin/linezolid/tigecycline therapy for tackling AMR and precision-based antibiotic therapy (Roberts et al., 2010; Gonçalves-Pereira and Póvoa, 2011; Roberts et al., 2014b) (Figures 4, 5).

Conventional statistical models often struggle to accommodate the non-linear, temporally dependent nature of such clinical data. ML approaches, however, excel at disentangling complex variable interdependencies. By leveraging high-dimensional temporal datasets, ML can generate dynamic patient-state representations, facilitating differentiation between stable and deteriorating conditions. This enables continuous, data-driven tracking of recovery trajectories and therapeutic response. Through such modelling, it becomes possible to quantify the influence of TDM interventions on clinical progression, integrating physiological, biochemical, and treatment-related variables into a unified analytical construct (Ates et al., 2020; Arnold et al., 2025; Ates et al., 2024; Li QY. et al., 2024).

ML-based inference to elucidate the relationship between TDM and patient recovery dynamics. Specifically, it aims to: (1) quantify patient-state variations during piperacillin/tazobactam therapy, (2) determine the influence of TDM-guided dosing on these dynamics, and (3) assess survival outcomes (Ates et al., 2020; Arnold et al., 2025; Ates et al., 2024; Li QY. et al., 2024). The computational biomarkers may serve as proxies for disease stratification and contribute to the development of a continuous, multidimensional alternative to the conventional Sequential Organ Failure Assessment (SOFA) score. The integration of ML-driven analyses thus reveals the concealed relationships between antibiotic exposure, host response, and therapeutic success, reinforcing the clinical value of data-informed TDM strategies pertinent to β-lactam/vancomycin/linezolid/tigecycline therapy for tackling AMR and precision-based antibiotic therapy (Ates et al., 2020; Arnold et al., 2025; Ates et al., 2024; Li QY. et al., 2024).

Broadly, ML serves as a complementary paradigm to mechanism-driven pharmacological modelling. As biomedical data from multi-omics, electronic health records, and real-world clinical settings expand, ML integration promises to enhance dose individualization and adaptive therapy optimization (Ates et al., 2020; Arnold et al., 2025; Ates et al., 2024; Li QY. et al., 2024). Based on analytical strategies, ML algorithms can be categorized into supervised, unsupervised, and reinforcement learning paradigms. Supervised learning employs labeled datasets to predict predefined outcomes, while unsupervised approaches explore unlabeled datasets to uncover hidden biological structures or relationships. Reinforcement learning, through iterative feedback and reward optimization, supports the discovery of adaptive therapeutic strategies (Ates et al., 2020; Arnold et al., 2025; Ates et al., 2024; Li QY. et al., 2024). Collectively, these advances illustrate that ML and AI approaches complement traditional mechanistic modelling by uncovering latent, data-driven relationships between therapy (Ates et al., 2020; Arnold et al., 2025; Ates et al., 2024; Li QY. et al., 2024), host response, and resistance evolution. As diverse, large-scale datasets continue to expand spanning clinical, genomic, and environmental domains ML-based frameworks will be integral to precision pharmacotherapy, enabling proactive AMR prediction, individualized dosing, and real-time therapeutic optimization of β-lactam/vancomycin/linezolid/tigecycline therapy for tackling AMR and precision-based antibiotic therapy (Ates et al., 2020; Arnold et al., 2025; Ates et al., 2024; Li QY. et al., 2024).

### Critical limitations of AI/ML integration in TDM

#### Challenges to clinical translation

Most currently available AI/ML-enabled TDM frameworks for last-resort antibiotics have been developed using relatively small, single-centre datasets, increasing susceptibility to overfitting and limiting model robustness across heterogeneous patient populations. Although several models demonstrate strong in-sample predictive performance, their clinical utility may diminish when applied to populations with differing demographics, comorbidities, organ dysfunction profiles, antimicrobial resistance patterns, and microbiological characteristics. Therefore, rigorous model development strategies incorporating appropriate cross-validation, calibration testing, and prospective external validation are essential before clinical implementation. Previous studies have emphasized that predictive AI models frequently fail to generalize adequately when trained on limited datasets (Kelly et al., 2019). Furthermore, external validation across large, multicentre, and ethnically diverse cohorts remains insufficient for the majority of AI-assisted TDM platforms, representing a major translational limitation in precision antibiotic dosing applications (Al-Ewaidat and Naffaa, 2025). Regulatory agencies such as the U.S. Food and Drug Administration and the European Medicines Agency increasingly mandate robust external validation and clinical evidence prior to deployment of AI-enabled clinical decision-support systems, yet only a limited number of current TDM algorithms satisfy these requirements, thereby hindering broader clinical adoption (De and Lohani, 2026).

Another important limitation involves the interpretability of advanced deep learning and ensemble-based predictive models. Many AI-driven TDM systems operate as “black-box” frameworks with limited mechanistic transparency, potentially reducing clinician trust and hindering adoption in high-acuity environments such as intensive care units, where therapeutic decisions require clear justification and traceability. In the context of critically ill patients receiving last-resort antibiotics, explainability and transparency are particularly important because dosing decisions directly influence toxicity risk, antimicrobial exposure, and therapeutic efficacy. Concerns regarding the lack of interpretability in clinical AI systems have been extensively discussed in biomedical AI literature (Topol, 2019). Similarly, the challenges associated with explainability and clinician acceptance of complex machine-learning frameworks in healthcare have also been highlighted in translational medical informatics studies (Khosravi et al., 2024). Consequently, explainable AI approaches and clinically interpretable hybrid pharmacometric-AI models are increasingly advocated to improve clinician confidence and facilitate bedside integration.

From a regulatory perspective, most AI-based dosing recommendation systems are now classified as Software as a Medical Device (AI-SaMD), requiring demonstration of analytical validity, clinical safety, algorithmic transparency, cybersecurity safeguards, and continuous post-market performance monitoring. However, the majority of currently reported AI-enabled TDM tools remain at an early developmental stage and have not yet fully addressed these stringent regulatory expectations, thereby limiting widespread clinical translation. Recent regulatory analyses have further emphasized that many AI-enabled healthcare systems still lack adequate evidence regarding safety, reproducibility, and lifecycle monitoring required for regulatory approval and real-world implementation (Ebad et al., 2025). Furthermore, existing AI/ML-assisted TDM frameworks have largely been developed in high-income healthcare settings with access to advanced laboratory infrastructure, high-quality electronic health records, and large longitudinal patient datasets. In contrast, low- and middle-income countries, which bear a disproportionately high burden of antimicrobial resistance, often face major challenges related to data quality, interoperability, digital infrastructure, and standardized clinical data acquisition. This disparity raises concerns regarding algorithmic bias, limited generalizability, and inequitable deployment of precision antibiotic monitoring technologies. Recent global digital-health studies have highlighted the urgent need for equitable and inclusive AI model development incorporating diverse datasets from underrepresented healthcare systems to improve fairness and global applicability (Ciecierski-Holmes et al., 2022). Therefore, future next-generation hybrid bioanalytical and AI-integrated TDM platforms should prioritize inclusive multicentre collaborations incorporating diverse LMIC datasets to ensure equitable, globally applicable precision dosing strategies for optimizing last-resort antibiotic therapy.

### Advances in mechanism-based PK/PD models for precision antibiotic dosing: towards integrating TDM in clinical decision-making

The integration of mechanism-based PK/PD models into TDM frameworks represents a transformative approach for optimizing antibiotic therapy. Unlike conventional PK/PD indices that rely on single-point summaries of drug exposure, mechanism-based models provide a dynamic, time-resolved depiction of drug-bacteria interactions (Wicha et al., 2021). This approach enables individualized dose optimization, incorporation of resistance development, and the rational design of combination regimens (Wicha et al., 2021). Here, recent advances in mechanism-based PK/PD modeling, their translational potential, and their emerging role in model-informed precision dosing (MIPD), highlighting opportunities for clinical implementation and future research directions. Traditional antibiotic dosing strategies often rely on population-based PK/PD indices, which correlate exposure metrics at single timepoints with clinical efficacy (Wicha et al., 2021; Nielsen and Friberg, 2013). While informative, these indices fail to capture the full temporal dynamics of drug–pathogen interactions, limiting their predictive power for individual patients (Wicha et al., 2021; Nielsen and Friberg, 2013). Mechanism-based PK/PD models overcome this limitation by simultaneously modeling the pharmacokinetics of the antibiotic and the pharmacodynamics of bacterial growth and killing, thereby providing a holistic framework for dose individualization related to β-lactam/vancomycin/linezolid/tigecycline therapy for tackling AMR (Wicha et al., 2021; Nielsen and Friberg, 2013). Figure 3 figure contrasts the traditional TDM workflow with the advanced model-informed precision dosing (MIPD) approach. Conventional TDM relies on empirical dosing, steady-state measurements, and delayed adjustments, often leading to therapeutic inefficiency. In contrast, MIPD incorporates PK/PD models, optimal sampling strategies, and real-time quantification to personalize antibiotic therapy. Using tools such as WinNonlin software, individualized PK parameters and microbial MIC values are integrated for predictive dose optimization. The inclusion of biomarkers like CRP and procalcitonin enhances infection severity assessment, supporting rapid, evidence-based adjustments. This model-driven framework accelerates clinical decision-making and reduces adverse drug events in intensive care settings (Figure 4).

### Components of Mechanism-Based PK/PD models

Mechanism-based models typically consist of three interrelated modules: (1) bacterial growth module characterizes intrinsic bacterial replication rates and natural clearance. (2) drug kinetics module describes temporal changes in drug concentrations within biological compartments. (3) effect module defines how drug exposure influences bacterial killing or growth inhibition. These models are often parameterized using *in vitro* time–kill curve experiments, which quantify bacterial dynamics under constant or dynamically varying drug concentrations that mimic clinical PK profiles (Nielsen and Friberg, 2013). Mechanism-based models offer several critical advantages over conventional approaches by retaining the temporal structure of PK/PD data, these models improve translational accuracy across species, accounting for interspecies differences in pharmacokinetics (Kristoffersson et al., 2016) and also by temporal modeling that facilitates the prediction of resistance emergence and allows integration into dosing optimization strategies (Rees et al., 2016). The inclusion of combination antibiotic PD interactions enables rational design of synergistic or additive regimens (Yadav et al., 2017; Aranzana-Climent et al., 2020). Both *in vitro* and *in vivo* datasets including immune response data can be combined, enhancing translational fidelity and supporting patient-specific dose adjustment (Sou et al., 2021).

Empirical studies demonstrate that mechanism-based models derived from *in vitro* data accurately replicate PK/PD index results obtained from dose-fractionation studies in neutropenic mouse models (Kristoffersson et al., 2016). Translating these findings to humans remains challenging due to variability in patient populations, infecting organisms, infection sites, and practical constraints in exploring multiple dosing regimens. Consequently, clinical validation of *in vitro* and preclinical targets remains limited, highlighting a critical area for ongoing research. Early characterization of bacterial parameters such as maximal killing rate, potency (EC50), and growth rate can enable bedside application of pre-existing models, even in the presence of sparse patient data (Wicha et al., 2021; Nielsen and Friberg, 2013).

### Role of TDM and MIPD in precision-antibiotic therapy

TDM is particularly valuable for vulnerable populations, including critically ill patients, and is recommended for antibiotics with narrow therapeutic windows, such as aminoglycosides, glycopeptides, beta-lactams, vancomycin/linezolid/tigecycline therapy for tackling AMR and precision-based antibiotic therapy. Mechanism-based MIPD approaches can streamline TDM workflows, enhance prediction accuracy, and allow integration of patient-specific biomarkers of treatment response and toxicity. Rapid and sensitive bioanalytical techniques are critical for implementing mechanism-based MIPD. Emerging strategies such as dried blood spot sampling, noninvasive fluid collection, and wearable sensors for real-time drug monitoring may overcome current logistical limitations. Well-designed prospective clinical trials are necessary to quantify the clinical and pharmacoeconomic benefits of precision dosing (Wicha et al., 2021; Nielsen and Friberg, 2013). Early incorporation of PK/PD-informed evaluation during drug development may accelerate identification of clinical scenarios in which precision dosing yields maximal benefit. Mechanism-based PK/PD models represent a paradigm shift in antibiotic dosing, bridging the gap between *in vitro*, preclinical, and clinical data. By integrating TDM, resistance dynamics, combination therapy effects, and patient-specific biomarkers, these models offer unprecedented opportunities to enhance individualized therapy and optimize clinical outcomes. Continued research and technological innovation will be pivotal in translating these models into routine clinical practice.

### Trials in TDM for precision therapeutic-based antibiotics

For instance, sepsis, a life-threatening syndrome characterized by dysregulated host immune responses to infection, continues to carry hospital mortality rates exceeding 40% (Hagel et al., 2019). Conventional antibiotic therapy often fails to achieve optimal pharmacokinetic/pharmacodynamic targets due to interindividual variability, delayed therapy initiation, or inappropriate dosing. Recent evidence supports the use of prolonged or continuous infusion strategies for β-lactam antibiotics, such as piperacillin/tazobactam (TZP), to improve drug exposure. Integrating TDM offers a personalized approach to dosing, potentially enhancing efficacy while minimizing toxicity.

TDM-guided dosing significantly enhances PK/PD target attainment in critically ill patients (Hagel et al., 2019), a critical determinant of antimicrobial efficacy. By individualizing therapy based on measured drug concentrations, clinicians can avoid underdosing that leads to treatment failure and overdosing that predisposes to toxicity. This approach is particularly relevant in the dynamic physiological context of sepsis, where conventional dosing may be inadequate. Furthermore, precision-guided therapy may reduce the emergence of antibiotic resistance by maintaining effective drug concentrations without unnecessary exposure. The study also highlights the practical feasibility of daily TDM in intensive care settings, reinforcing its potential as a standard of care for high-risk patients receiving β-lactam antibiotics. Integration with continuous infusion regimens may further optimize time-dependent killing and improve clinical outcomes (Hagel et al., 2019). Daily TDM-guided dosing of meropenem or piperacillin/tazobactam improves PK/PD target attainment and supports the resolution of organ dysfunction, outperforming conventional dosing strategies. Adoption of TDM in clinical practice may enhance patient outcomes, reduce toxicity, and mitigate antimicrobial resistance, providing a foundation for precision-guided critical care therapeutics.

A prospective, partially blinded, randomized controlled trial was conducted in critically ill patients with normal renal function receiving either meropenem (MEM) or piperacillin/tazobactam (PTZ). Patients in the intervention arm underwent daily TDM, with dose adjustments performed as indicated by real-time PK measurements. The predefined PD target was 100% fT > 4×MIC, representing the percentage of time during the dosing interval in which free drug concentrations exceeded four times the MIC (De Waele et al., 2014). Daily TDM-guided dosing in critically ill patients with normal renal function significantly improves PK/PD target attainment compared to conventional dosing strategies. By tailoring therapy to individual PK profiles, this approach not only enhances antimicrobial efficacy but may also reduce the risk of toxicity and the emergence of resistance, supporting its integration into precision antibiotic therapy protocols (De Waele et al., 2014).

### Precision dosing to counter β-lactam resistance

The recent trials investigating TDM-guided therapy of β-lactam antibiotics, such as piperacillin/tazobactam and meropenem, highlight the critical role of individualized dosing in mitigating antimicrobial resistance. In critically ill patients, conventional dosing often fails to achieve pharmacodynamic targets due to altered pharmacokinetics, leading to subinhibitory antibiotic exposure, a key driver of resistance selection. Daily TDM enables dose optimization to maintain drug concentrations above the MIC for extended periods, enhancing bacterial killing while minimizing selective pressure for resistant strains. Continuous or prolonged infusion strategies, combined with real-time TDM, provide a precision approach that could be generalized to other time-dependent antibiotics, ensuring maximal efficacy and reduced emergence of β-lactam-resistant pathogens.

### Optimizing tetracycline use through PK/PD guidance

Tetracycline-class antibiotics, including doxycycline and minocycline, exhibit complex pharmacokinetics influenced by absorption variability and tissue penetration. Subtherapeutic exposure in critically ill or obese patients may inadvertently promote resistance, particularly among Gram-negative pathogens. Leveraging insights from TDM-based β-lactam trials, adaptive dosing guided by PK/PD monitoring could ensure sustained therapeutic concentrations of tetracyclines, particularly in tissues where bacterial biofilms form. Personalized dosing strategies may also minimize off-target effects that contribute to microbiome disruption, a recognized contributor to horizontal gene transfer and resistance propagation.

### Vancomycin and targeted concentration management

Glycopeptide antibiotics like vancomycin pose significant challenges due to narrow therapeutic windows and nephrotoxicity risks. TDM-guided strategies have already been recognized as essential in vancomycin stewardship. Integrating lessons from β-lactam PK/PD trials, precise measurement of trough or AUC values allows clinicians to maintain bactericidal concentrations against MRSA while preventing underexposure that fosters vancomycin-intermediate or -resistant *Staphylococcus aureus* (VISA/VRSA). By dynamically adjusting doses based on real-time PK data, TDM not only enhances efficacy but also extends the useful lifespan of vancomycin as a critical agent in AMR management.

### Linezolid and Tigecycline use beyond conventional dosing

For oxazolidinones like linezolid and glycylcyclines such as tigecycline, prolonged or suboptimal dosing can rapidly select for resistant strains. Linezolid’s reversible myelosuppression often limits dosage escalation, while tigecycline’s low serum concentrations may be insufficient against bloodstream infections. Incorporating TDM-informed dose adjustment, inspired by recent β-lactam trials, allows clinicians to balance efficacy and toxicity, particularly in critically ill patients with variable drug clearance. These strategies are crucial for preserving the activity of last-line antibiotics and delaying the spread of multidrug-resistant organisms.

### Integrating TDM into a comprehensive AMR strategy

Collectively, these findings demonstrate the importance of a precision-based, TDM-informed framework in combating AMR across multiple antibiotic classes. By ensuring optimal PK/PD target attainment, healthcare providers can maximize bactericidal activity while reducing selective pressures that drive resistance. When combined with antimicrobial stewardship programs, infection source control, and rapid diagnostic testing, TDM-guided therapy provides a rational and evidence-based approach to antibiotic management. Future research should focus on expanding TDM protocols to a broader spectrum of antibiotics, integrating real-time resistance surveillance, and developing predictive PK/PD models to support dynamic dosing in diverse patient populations, ultimately enhancing the effectiveness of interventions against AMR.

## Conclusion

Therapeutic drug monitoring is evolving beyond conventional analytical methods into a sophisticated, AI-augmented precision medicine tool. The integration of LC–MS/MS, biosensors, microneedle platforms, and PK/PD modeling offers unprecedented opportunities to optimize antibiotic dosing in critically ill patients. This integrated TDM framework not only enhances clinical efficacy and safety but also plays a strategic role in combating the growing threat of antimicrobial resistance.

### Future directions

Large-scale clinical trials are needed to validate the proposed three-tiered TDM model and establish standardized protocols for bedside implementation. Further development of portable, multiplexed biosensors and wearable devices will enhance real-time monitoring capabilities. Expanding AI and machine learning frameworks will enable adaptive, patient-specific dosing recommendations and predictive therapeutic outcomes. Development of regulatory pathways and healthcare infrastructure to support decentralized TDM platforms will accelerate clinical adoption. Embedding advanced TDM technologies within antimicrobial stewardship programs can help shape a proactive, data-driven response to the global AMR crisis.

## References

[B1] Abdul-Aziz M. H. Alffenaar J.-W. C. Bassetti M. Bracht H. Dimopoulos G. Marriott D. (2020). Antimicrobial therapeutic drug monitoring in critically ill adult patients: a position paper. Intensive Care Medicine 46 (6), 1127–1153. 10.1007/s00134-020-06050-1 32383061 PMC7223855

[B2] Abdulla A. Bahmany S. Wijma R. A. van der Nagel B. C. Koch B. C. (2017). Simultaneous determination of nine β-lactam antibiotics in human plasma by an ultrafast hydrophilic-interaction chromatography–tandem mass spectrometry. J. Chromatogr. B 1060, 138–143. 10.1016/j.jchromb.2017.06.014 28618388

[B3] Abdulla A. Ewoldt T. Hunfeld N. Muller A. Rietdijk W. Polinder S. (2020). The effect of therapeutic drug monitoring of beta-lactam and fluoroquinolones on clinical outcome in critically ill patients: the DOLPHIN trial protocol of a multi-centre randomised controlled trial. BMC Infectious Diseases 20 (1), 57. 10.1186/s12879-020-4781-x 31952493 PMC6969462

[B4] Adnan S. Li J. X. Wallis S. C. Rudd M. Jarrett P. Paterson D. L. (2013). Pharmacokinetics of meropenem and piperacillin in critically ill patients with indwelling surgical drains. Int. J. Antimicrob. Agents 42 (1), 90–93. 10.1016/j.ijantimicag.2013.02.023 23590897

[B5] Akimoto J. Nakayama M. Okano T. (2014). Temperature-responsive polymeric micelles for optimizing drug targeting to solid tumors. J. Controlled Release 193, 2–8. 10.1016/j.jconrel.2014.06.062 25037017

[B6] Al-Ewaidat O. A. Naffaa M. M. (2025). Emerging AI-and biomarker-driven precision medicine in autoimmune rheumatic diseases: from diagnostics to therapeutic decision-making. Rheumato 5 (4), 17. 10.3390/rheumato5040017

[B7] Alqahtani S. A. Alsultan A. S. Alqattan H. M. Eldemerdash A. Albacker T. B. (2018). Population pharmacokinetic model for vancomycin used in open heart surgery: model-based evaluation of standard dosing regimens. Antimicrob. Agents Chemotherapy 62 (7), e00088-18. 10.1128/aac.00088-00018 PMC6021682 29686154

[B8] Anema J. R. Li J.-F. Yang Z.-L. Ren B. Tian Z.-Q. (2011). Shell-isolated nanoparticle-enhanced raman spectroscopy: expanding the versatility of surface-enhanced raman scattering. Annu. Rev. Anal. Chem. 4 (1), 129–150. 10.1146/annurev.anchem.111808.073632 21370987

[B9] Anker J. N. Hall W. P. Lyandres O. Shah N. C. Zhao J. Van Duyne R. P. (2008). Biosensing with plasmonic nanosensors. Nat. Materials 7 (6), 442–453. 10.1038/nmat2162 18497851

[B10] Annoni F. Grimaldi D. Taccone F. S. (2020). Individualized antibiotic therapy in the treatment of severe infections. Expert Rev. Anti-infective Ther. 18 (1), 27–35. 10.1080/14787210.2020.1696192 31755789

[B11] Aranzana-Climent V. Buyck J. M. Smani Y. Pachón-Diaz J. Marchand S. Couet W. (2020). Semi-mechanistic PK/PD modelling of combined polymyxin B and minocycline against a polymyxin-resistant strain of Acinetobacter baumannii. Clin. Microbiol. Infect. 26 (9), 1254–1259. 10.1016/j.cmi.2020.01.017 32006693

[B12] Arnold A. McLellan S. Stokes J. M. (2025). How AI can help Us beat AMR. Npj Antimicrob. Resist. 3 (1), 18. 10.1038/s44259-025-00085-4 40082590 PMC11906734

[B13] Ates H. C. Roberts J. A. Lipman J. Cass A. E. Urban G. A. Dincer C. (2020). On-site therapeutic drug monitoring. Trends Biotechnology 38 (11), 1262–1277. 10.1016/j.tibtech.2020.03.001 33058758

[B14] Ates H. C. Mohsenin H. Wenzel C. Glatz R. T. Wagner H. J. Bruch R. (2022). Biosensor‐enabled multiplexed on‐site therapeutic drug monitoring of antibiotics. Adv. Mater. 34 (2), 2104555. 10.1002/adma.202104555 34545651 PMC11468941

[B15] Ates H. C. Alshanawani A. Hagel S. Cotta M. O. Roberts J. A. Dincer C. (2024). Unraveling the impact of therapeutic drug monitoring *via* machine learning for patients with sepsis. Cell Rep. Med. 5 (8), 101681. 10.1016/j.xcrm.2024.101681 39127039 PMC11384951

[B17] Bantz K. C. Meyer A. F. Wittenberg N. J. Im H. Kurtuluş Ö. Lee S. H. (2011). Recent progress in SERS biosensing. Phys. Chemistry Chemical Physics 13 (24), 11551–11567. 10.1039/c0cp01841d 21509385 PMC3156086

[B18] Barco S. Castagnola E. Gennai I. Barbagallo L. Loy A. Tripodi G. (2016). Ultra high performance liquid chromatography-tandem mass spectrometry vs. commercial immunoassay for determination of vancomycin plasma concentration in children. Possible implications for everyday clinical practice. J. Chemotherapy 28 (5), 395–402. 10.1080/1120009X.2016.1157947 27238431

[B19] Bellouard R. Deslandes G. Morival C. Li J. Boutoille D. Jolliet P. (2020). Simultaneous determination of eight β-lactam antibiotics in human plasma and cerebrospinal fluid by liquid chromatography coupled to tandem mass spectrometry. J. Pharm. Biomed. Analysis 178, 112904. 10.1016/j.jpba.2019.112904 31606563

[B20] Beumier M. Casu G. S. Hites M. Seyler L. Cotton F. Vincent J.-L. (2014). β-lactam antibiotic concentrations during continuous renal replacement therapy. Crit. Care 18 (3), R105. 10.1186/cc13886 24886826 PMC4075122

[B21] Bian L. Liang J. Zhao H. Ye K. Li Z. Liu T. (2021). Rapid monitoring of vancomycin concentration in serum using europium (III) chelate nanoparticle-based lateral flow immunoassay. Front. Chem. 9, 763686. 10.3389/fchem.2021.763686 34733823 PMC8558538

[B22] Bidu N. S. Fernandes B. J. Bastos R. E. Pedreira J. N. Couto R. D. (2020). Should the vancomycin minimal inhibitory concentration be used as an infant critical care regular criteria? Curr. Pharm. Biotechnol. 21 (11), 1052–1058. 10.2174/1389201021666200327162402 32216735

[B23] Bonifacio A. Dalla Marta S. Spizzo R. Cervo S. Steffan A. Colombatti A. (2014). Surface-enhanced Raman spectroscopy of blood plasma and serum using Ag and Au nanoparticles: a systematic study. Anal. Bioanal. Chem. 406 (9), 2355–2365. 10.1007/s00216-014-7622-1 24493335

[B24] Bordbar M. M. Samadinia H. Hajian A. Sheini A. Safaei E. Aboonajmi J. (2022). Mask assistance to colorimetric sniffers for detection of Covid-19 disease using exhaled breath metabolites. Sensors Actuators B Chem. 369, 132379. 10.1016/j.snb.2022.132379 PMC9279257 35855726

[B25] Brasier N. Eckstein J. (2020). Sweat as a source of next-generation digital biomarkers. Digit. Biomarkers 3 (3), 155–165. 10.1159/000504387 32095774 PMC7011725

[B26] Brasier N. Ates H. C. Sempionatto J. R. Cotta M. O. Widmer A. F. Eckstein J. (2023). A three-level model for therapeutic drug monitoring of antimicrobials at the site of infection. Lancet Infect. Dis. 23 (10), e445–e453. 10.1016/s1473-3099(23)00215-3 37348517

[B27] Brink A. J. (2012). Does resistance in severe infections caused by methicillin-resistant *Staphylococcus aureus* give you the ‘creeps’? Curr. Opinion Critical Care 18 (5), 451–459. 10.1097/MCC.0b013e3283578968 22941206

[B28] Brun M. A. Tan K.-T. Nakata E. Hinner M. J. Johnsson K. (2009). Semisynthetic fluorescent sensor proteins based on self-labeling protein tags. J. Am. Chem. Soc. 131 (16), 5873–5884. 10.1021/ja900149e 19348459

[B29] Brun M. A. Griss R. Reymond L. Tan K.-T. Piguet J. Peters R. J. (2011). Semisynthesis of fluorescent metabolite sensors on cell surfaces. J. Am. Chem. Soc. 133 (40), 16235–16242. 10.1021/ja206915m 21879732

[B30] Brun M. A. Tan K.-T. Griss R. Kielkowska A. Reymond L. Johnsson K. (2012). A semisynthetic fluorescent sensor protein for glutamate. J. Am. Chem. Soc. 134 (18), 7676–7678. 10.1021/ja3002277 22533301

[B31] Brunet M. Van Gelder T. Åsberg A. Haufroid V. Hesselink D. A. Langman L. (2019). Therapeutic drug monitoring of tacrolimus-personalized therapy: second consensus report. Ther. Drug Monitoring 41 (3), 261–307. 10.1097/FTD.0000000000000640 31045868

[B32] Bushey M. M. Jorgenson J. W. (1990). Automated instrumentation for comprehensive two-dimensional high-performance liquid chromatography of proteins. Anal. Chemistry 62 (2), 161–167. 10.1021/ac00201a015 2310013

[B33] Cafaro A. Conti M. Pigliasco F. Barco S. Bandettini R. Cangemi G. (2023). Biological fluid microsampling for therapeutic drug monitoring: a narrative review. Biomedicines 11 (7), 1962. 10.3390/biomedicines11071962 37509602 PMC10377272

[B34] Cafaro A. Barco S. Pigliasco F. Russo C. Mariani M. Mesini A. (2024). Therapeutic drug monitoring of glycopeptide antimicrobials: an overview of liquid chromatography-tandem mass spectrometry methods. J. Mass Spectrom. Adv. Clin. Lab 31, 33–39. 10.1016/j.jmsacl.2023.12.003 38304144 PMC10831154

[B35] Calucho E. Parolo C. Rivas L. Álvarez-Diduk R. Merkoçi A. (2020). Nanoparticle-based lateral flow assays. In: Comprehensive Analytical Chemistry. Volume 89, edn.: Elsevier; 313–359.

[B36] Cangemi G. Barbagallo L. Cafaro A. Pigliasco F. Goffredo M. Di Paolo A. (2022). Indications for the harmonization of the therapeutic intervals of antibacterial drugs. Biochim. Clin. 46, 347–353. Available online at: https://hdl.handle.net/11567/1204195.

[B37] Cao Y. C. Jin R. Mirkin C. A. (2002). Nanoparticles with raman spectroscopic fingerprints for DNA and RNA detection. Science 297 (5586), 1536–1540. 10.1126/science.297.5586.1536 12202825

[B38] Carvalho V. M. (2012). The coming of age of liquid chromatography coupled to tandem mass spectrometry in the endocrinology laboratory. J. Chromatogr. B 883, 50–58. 10.1016/j.jchromb.2011.08.027 21907642

[B39] Castagnola E. Cangemi G. Mesini A. Castellani C. Martelli A. Cattaneo D. (2021). Pharmacokinetics and pharmacodynamics of antibiotics in cystic fibrosis: a narrative review. Int. Journal Antimicrobial Agents 58 (3), 106381. 10.1016/j.ijantimicag.2021.106381 34157401

[B40] Cattaneo D. Corona A. De Rosa F. G. Gervasoni C. Kocic D. Marriott D. J. (2020). The management of anti-infective agents in intensive care units: the potential role of a ‘fast’pharmacology. Expert Rev. Clin. Pharmacol. 13 (4), 355–366. 10.1080/17512433.2020.1759413 32320302

[B41] Chan K. M. Gleadle J. M. O’Callaghan M. Vasilev K. MacGregor M. (2022). Prostate cancer detection: a systematic review of urinary biosensors. Prostate Cancer Prostatic Dis. 25 (1), 39–46. 10.1038/s41391-021-00480-8 34997229

[B42] Chauhan K. P. Trivedi A. P. Patel D. Gami B. Haridas N. (2014). Monitoring and root cause analysis of clinical biochemistry turn around time at an academic hospital. Indian J. Clin. Biochem. 29 (4), 505–509. 10.1007/s12291-013-0397-x 25298634 PMC4175690

[B43] Chen Q. Chen Z. Liu D. He Z. Wu J. (2020). Constructing an E-nose using metal-ion-induced assembly of graphene oxide for diagnosis of lung cancer *via* exhaled breath. ACS Applied Materials and Interfaces 12 (15), 17713–17724. 10.1021/acsami.0c00720 32203649

[B44] Cheng N. Song Y. Zeinhom M. M. Chang Y.-C. Sheng L. Li H. (2017). Nanozyme-mediated dual immunoassay integrated with smartphone for use in simultaneous detection of pathogens. ACS Appl. Mater. and Interfaces 9 (46), 40671–40680. 10.1021/acsami.7b12734 28914522 PMC8681872

[B45] Ciecierski-Holmes T. Singh R. Axt M. Brenner S. Barteit S. (2022). Artificial intelligence for strengthening healthcare systems in low-and middle-income countries: a systematic scoping review. NPJ Digital Medicine 5 (1), 162. 10.1038/s41746-022-00700-y 36307479 PMC9614192

[B46] Coetzee L. M. Tepper M. E. Perelson L. Glencross D. K. Cassim N. (2020). Timely delivery of laboratory efficiency information, part II: assessing the impact of a turn-around time dashboard at a high-volume laboratory. Afr. J. Laboratory Med. 9 (2), 1–8. 10.4102/ajlm.v9i2.948 32391245 PMC7203269

[B47] Colin P. De Bock L. T'jollyn H. Boussery K. Van Bocxlaer J. (2013). Development and validation of a fast and uniform approach to quantify β-lactam antibiotics in human plasma by solid phase extraction-liquid chromatography–electrospray-tandem mass spectrometry. Talanta 103, 285–293. 10.1016/j.talanta.2012.10.046 23200389

[B48] De A. Lohani A. (2026). Regulatory adoption of AI, ML, computational modeling and simulation in *in-silico* clinical trials for medical devices: a systematic review. Ther. Innovation and Regul. Sci. 60 (1), 45–62. 10.1007/s43441-025-00871-2 41055689

[B49] de Jesús Valle M. J. López F. G. Navarro A. S. (2008). Development and validation of an HPLC method for vancomycin and its application to a pharmacokinetic study. J. Pharmaceutical Biomedical Analysis 48 (3), 835–839. 10.1016/j.jpba.2008.05.040 18639406

[B50] De Pascale G. Montini L. Pennisi M. A. Bernini V. Maviglia R. Bello G. (2014). High dose tigecycline in critically ill patients with severe infections due to multidrug-resistant bacteria. Crit. Care 18 (3), R90. 10.1186/cc13858 24887101 PMC4057423

[B51] De Waele J. J. Carrette S. Carlier M. Stove V. Boelens J. Claeys G. (2014). Therapeutic drug monitoring-based dose optimisation of piperacillin and meropenem: a randomised controlled trial. Intensive Care Medicine 40 (3), 380–387. 10.1007/s00134-013-3187-2 24356862

[B52] Donadello K. Antonucci E. Cristallini S. Roberts J. A. Beumier M. Scolletta S. (2015). β-Lactam pharmacokinetics during extracorporeal membrane oxygenation therapy: a case–control study. Int. Journal Antimicrobial Agents 45 (3), 278–282. 10.1016/j.ijantimicag.2014.11.005 25542059

[B53] Dorofaeff T. Bandini R. M. Lipman J. Ballot D. E. Roberts J. A. Parker S. L. (2016). Uncertainty in antibiotic dosing in critically ill neonate and pediatric patients: can microsampling provide the answers? Clin. Therapeutics 38 (9), 1961–1975. 10.1016/j.clinthera.2016.07.093 27544661

[B54] Durães F. Sousa E. (2019). Omadacycline: a newly approved antibacterial from the class of tetracyclines. Pharmaceuticals 12 (2), 63. 10.3390/ph12020063 31010063 PMC6630996

[B55] Ebad S. A. Alhashmi A. Amara M. Miled A. B. Saqib M. (2025). Artificial intelligence-based software as a medical device (AI-SaMD): a systematic review. In: Healthcare: 2025: (Basel, Switzerland: MDPI), 817. 10.3390/healthcare13070817 PMC11988595 40218113

[B56] Eckardt J.-N. Wendt K. Bornhaeuser M. Middeke J. M. (2021). Reinforcement learning for precision oncology. Cancers 13 (18), 4624. 10.3390/cancers13184624 34572853 PMC8472712

[B57] Edwards B. Milne K. Lawes T. Cook I. Robb A. Gould I. (2012). Is vancomycin MIC “creep” method dependent? Analysis of methicillin-resistant *Staphylococcus aureus* susceptibility trends in blood isolates from north east Scotland from 2006 to 2010. J. Clinical Microbiology 50 (2), 318–325. 10.1128/JCM.05520-11 22135252 PMC3264194

[B58] El-Gaml R. M. El-Khodary N. M. Abozahra R. R. El-Tayar A. A. El-Masry S. M. (2022). Applying pharmacokinetic/pharmacodynamic measurements for linezolid in critically ill patients: optimizing efficacy and reducing resistance occurrence. Eur. J. Clin. Pharmacol. 78 (8), 1301–1310. 10.1007/s00228-022-03340-z 35610318 PMC9283351

[B59] Evans L. Rhodes A. Alhazzani W. Antonelli M. Coopersmith C. M. French C. (2021). Surviving sepsis campaign: international guidelines for management of sepsis and septic shock 2021. Crit. Care Medicine 49 (11), e1063–e1143. 10.1097/ccm.0000000000005337 34605781

[B60] Eyler R. F. Shvets K. (2019). Clinical pharmacology of antibiotics. Clin. J. Am. Soc. Nephrol. 14 (7), 1080–1090. 10.2215/CJN.08140718 30862698 PMC6625637

[B61] Fang Z. Zhang H. Guo J. Guo J. (2024). Overview of therapeutic drug monitoring and clinical practice. Talanta 266, 124996. 10.1016/j.talanta.2023.124996 37562225

[B62] Farin D. Piva G. A. Gozlan I. Kitzes-Cohen R. (1998). A modified HPLC method for the determination of vancomycin in plasma and tissues and comparison to FPIA (TDX). J. Pharmaceutical Biomedical Analysis 18 (3), 367–372. 10.1016/s0731-7085(98)00095-8 10096831

[B63] Flarakos J. Du Y. Gu H. Wang L. Einolf H. J. Chun D. Y. (2017). Clinical disposition, metabolism and *in vitro* drug–drug interaction properties of omadacycline. Xenobiotica 47 (8), 682–696. 10.1080/00498254.2016.1213465 27499331

[B64] Gatti M. Cojutti P. G. Bartoletti M. Tonetti T. Bianchini A. Ramirez S. (2022). Expert clinical pharmacological advice may make an antimicrobial TDM program for emerging candidates more clinically useful in tailoring therapy of critically ill patients. Crit. Care 26 (1), 178. 10.1186/s13054-022-04050-9 35701812 PMC9199203

[B65] Gonçalves-Pereira J. Póvoa P. (2011). Antibiotics in critically ill patients: a systematic review of the pharmacokinetics of β-lactams. Crit. Care 15 (5), R206. 10.1186/cc10441 21914174 PMC3334750

[B66] Goswami B. Singh B. Chawla R. Gupta V. Mallika V. (2010). Turn around time (TAT) as a benchmark of laboratory performance. Indian J. Clin. Biochem. 25 (4), 376–379. 10.1007/s12291-010-0056-4 21966108 PMC2994570

[B67] Griss R. Schena A. Reymond L. Patiny L. Werner D. Tinberg C. E. (2014). Bioluminescent sensor proteins for point-of-care therapeutic drug monitoring. Nat. Chemical Biology 10 (7), 598–603. 10.1038/nchembio.1554 24907901

[B68] Groenendaal W. Von Basum G. Schmidt K. A. Hilbers P. A. Van Riel N. A. (2010). Quantifying the Composition of Human Skin for Glucose Sensor Development. Los Angeles, CA: Sage Publications Sage CA. 10.1177/193229681000400502 PMC2956818 20920423

[B69] Gross A. S. (1998). Best practice in therapeutic drug monitoring. Br. Journal Clinical Pharmacology 46 (2), 95–99. 10.1046/j.1365-2125.1998.00770.x 9723816 PMC1873661

[B70] Guerra V. Y. Dorofaeff T. Parker L. Coulthard M. G. Sparkes L. Lipman J. (2022). Microsampling to support pharmacokinetic clinical studies in pediatrics. Pediatr. Res. 91 (6), 1557–1561. 10.1038/s41390-021-01586-4 34023854

[B71] Guler E. Yilmaz Sengel T. Gumus Z. P. Arslan M. Coskunol H. Timur S. (2017). Mobile phone sensing of cocaine in a lateral flow assay combined with a biomimetic material. Anal. Chemistry 89 (18), 9629–9632. 10.1021/acs.analchem.7b03017 28831804

[B72] Herregodts J. Van Vooren S. Deschuyteneer E. Dhaese S. Stove V. Verstraete A. (2019). Measuring antibiotics in exhaled air in critically ill, non-ventilated patients: a feasibility and proof of concept study. J. Crit. Care 51, 46–50. 10.1016/j.jcrc.2019.01.025 30745285

[B73] Hagel S. Fiedler S. Hohn A. Brinkmann A. Frey O. R. Hoyer H. (2019). Therapeutic drug monitoring-based dose optimisation of piperacillin/tazobactam to improve outcome in patients with sepsis (TARGET): a prospective, multi-centre, randomised controlled trial. Trials 20 (1), 330. 10.1186/s13063-019-3437-x 31171029 PMC6554958

[B74] Hagel S. Bach F. Brenner T. Bracht H. Brinkmann A. Annecke T. (2022). Effect of therapeutic drug monitoring-based dose optimization of piperacillin/tazobactam on sepsis-related organ dysfunction in patients with sepsis: a randomized controlled trial. Intensive Care Medicine 48 (3), 311–321. 10.1007/s00134-021-06609-6 35106617 PMC8866359

[B75] Hale C. M. Seabury R. W. Steele J. M. Darko W. Miller C. D. (2017). Are vancomycin trough concentrations of 15 to 20 mg/L associated with increased attainment of an AUC/MIC≥ 400 in patients with presumed MRSA infection? J. Pharm. Pract. 30 (3), 329–335. 10.1177/0897190016642692 27074786

[B76] Halperin A. Kröger M. Winnik F. M. (2015). Poly (N‐isopropylacrylamide) phase diagrams: fifty years of research. Angew. Chem. Int. Ed. 54 (51), 15342–15367. 10.1002/anie.201506663 26612195

[B77] Hierholzer W. J. Garner J. S. Adams A. B. Craven D. E. Fleming D. W. Forlenza S. W. (1995). Recommendations for preventing the spread of vancomycin resistance: recommendations of the hospital infection control practices advisory committee (HICPAC). Am. J. Infect. Control 23, 87–94. 10.1016/0196-6553(95)90104-3 7639408

[B78] Himmelsbach M. (2012). 10 years of MS instrumental developments–impact on LC–MS/MS in clinical chemistry. J. Chromatogr. B 883, 3–17. 10.1016/j.jchromb.2011.11.038 22177236

[B79] Homma K. Masuda T. Akimoto A. M. Nagase K. Itoga K. Okano T. (2017). Fabrication of micropatterned self‐oscillating polymer brush for direction control of chemical waves. Small 13 (21), 1700041. 10.1002/smll.201700041 28383186

[B80] Hu C. Wang W. Jo J. Garey K. W. (2024). Development and validation of LC-MS/MS for quantifying omadacycline from stool for gut microbiome studies. J. Chromatogr. B 1236, 124057. 10.1016/j.jchromb.2024.124057 38447241

[B81] Hui L.-A. Bodolea C. Vlase L. Hiriscau E. I. Popa A. (2022). Linezolid administration to critically ill patients: intermittent or continuous infusion? A systematic literature search and review. Antibiotics 11 (4), 436. 10.3390/antibiotics11040436 35453188 PMC9025826

[B82] Hui L.-A. Bodolea C. Popa A. Vlase A.-M. Hirişcău E. I. Vlase L. (2024). Linezolid pharmacokinetics in critically ill patients: continuous *versus* intermittent infusion. Antibiotics 13 (10), 961. 10.3390/antibiotics13100961 39452227 PMC11504488

[B83] Ibrahim E. H. Sherman G. Ward S. Fraser V. J. Kollef M. H. (2000). The influence of inadequate antimicrobial treatment of bloodstream infections on patient outcomes in the ICU setting. Chest 118 (1), 146–155. 10.1378/chest.118.1.146 10893372

[B84] Jacinto M. J. Trabuco J. R. Vu B. V. Garvey G. Khodadady M. Azevedo A. M. (2018). Enhancement of lateral flow assay performance by electromagnetic relocation of reporter particles. PLoS One 13 (1), e0186782. 10.1371/journal.pone.0186782 29309424 PMC5757911

[B85] Jacobs A. Taccone F. S. Roberts J. A. Jacobs F. Cotton F. Wolff F. (2018). β-Lactam dosage regimens in septic patients with augmented renal clearance. Antimicrob. Agents Chemotherapy 62 (9), e02534–e02617. 10.1128/AAC.02534-17 29987138 PMC6125556

[B86] Jager N. G. van Hest R. M. Lipman J. Taccone F. S. Roberts J. A. (2016). Therapeutic drug monitoring of anti-infective agents in critically ill patients. Expert Review Clinical Pharmacology 9 (7), 961–979. 10.1586/17512433.2016.1172209 27018631

[B87] Jager N. G. van Hest R. M. Lipman J. Roberts J. A. Cotta M. O. (2019). Antibiotic exposure at the site of infection: principles and assessment of tissue penetration. Expert Review Clinical Pharmacology 12 (7), 623–634. 10.1080/17512433.2019.1621161 31136211

[B88] Jarvis W. R. (1998). Epidemiology, appropriateness, and cost of vancomycin use. Rev. Infect. Dis. 26 (5), 1200–1203. 10.1086/520284 9597253

[B90] Jeon H.-J. Kim S. Park S. Jeong I.-K. Kang J. Kim Y. R. (2021). Optical assessment of tear glucose by smart biosensor based on nanoparticle embedded contact lens. Nano Lett. 21 (20), 8933–8940. 10.1021/acs.nanolett.1c01880 34415172

[B91] Joukhadar C. Frossard M. Mayer B. X. Brunner M. Klein N. Siostrzonek P. (2001). Impaired target site penetration of β-lactams may account for therapeutic failure in patients with septic shock. Crit. Care Medicine 29 (2), 385–391. 10.1097/00003246-200102000-00030 11246321

[B92] Kalil A. C. Van Schooneveld T. C. Fey P. D. Rupp M. E. (2014). Association between vancomycin minimum inhibitory concentration and mortality among patients with *Staphylococcus aureus* bloodstream infections: a systematic review and meta-analysis. Jama 312 (15), 1552–1564. 10.1001/jama.2014.6364 25321910

[B93] Kanazawa H. Yamamoto K. Matsushima Y. Takai N. Kikuchi A. Sakurai Y. (1996). Temperature-responsive chromatography using poly (N-isopropylacrylamide)-modified silica. Anal. Chem. 68 (1), 100–105. 10.1021/ac950359j 21619225

[B94] Kang J.-S. Lee M.-H. (2009). Overview of therapeutic drug monitoring. Korean Journal Internal Medicine 24 (1), 1–10. 10.3904/kjim.2009.24.1.1 19270474 PMC2687654

[B95] Karu N. Deng L. Slae M. Guo A. C. Sajed T. Huynh H. (2018). A review on human fecal metabolomics: methods, applications and the human fecal metabolome database. Anal. Chimica Acta 1030, 1–24. 10.1016/j.aca.2018.05.031 30032758

[B96] Kelly C. J. Karthikesalingam A. Suleyman M. Corrado G. King D. (2019). Key challenges for delivering clinical impact with artificial intelligence. BMC Medicine 17 (1), 195. 10.1186/s12916-019-1426-2 31665002 PMC6821018

[B97] Kenaan A. Li K. Barth I. Johnson S. Song J. Krauss T. F. (2020). Guided mode resonance sensor for the parallel detection of multiple protein biomarkers in human urine with high sensitivity. Biosens. Bioelectron. 153, 112047. 10.1016/j.bios.2020.112047 31999559

[B98] Khatib M. Haick H. (2022). Sensors for volatile organic compounds. ACS Nano 16 (5), 7080–7115. 10.1021/acsnano.1c10827 35511046

[B99] Khoubnasabjafari M. Fathi-Azarbayjani A. Rahimpour E. Jouyban-Gharamaleki V. Kim H. Y. Alffenaar J. (2021). Concentration profile of tobramycin in exhaled breath condensate after inhalation of a single dose: a pilot study. J. Drug Deliv. Sci. Technol. 62, 102394. 10.1016/j.jddst.2021.102394

[B16] Khosravi M. Zare Z. Mojtabaeian S. M. Izadi R. (2024). Artificial intelligence and decision-making in healthcare: a thematic analysis of a systematic review of reviews. Health Serv. Res. Manag. Epidemiol. 11, 1–15. 10.1177/23333928241234863 PMC10916499 38449840

[B100] Kiang T. K. Schmitt V. Ensom M. H. Chua B. Häfeli U. O. (2012). Therapeutic drug monitoring in interstitial fluid: a feasibility study using a comprehensive panel of drugs. J. Pharmaceutical Sciences 101 (12), 4642–4652. 10.1002/jps.23309 22941939

[B101] Kim J. Campbell A. S. de Ávila B. E.-F. Wang J. (2019). Wearable biosensors for healthcare monitoring. Nat. Biotechnology 37 (4), 389–406. 10.1038/s41587-019-0045-y 30804534 PMC8183422

[B102] Kiriazopoulos E. Zaharaki S. Vonaparti A. Vourna P. Panteri‐Petratou E. Gennimata D. (2017). Quantification of three beta‐lactam antibiotics in breast milk and human plasma by hydrophilic interaction liquid chromatography/positive‐ion electrospray ionization mass spectrometry. Drug Test. Analysis 9 (7), 1062–1072. 10.1002/dta.2104 27714984

[B103] Klapkova E. Nescakova M. Melichercik P. Jahoda D. Dunovska K. Cepova J. (2020). Vancomycin and its crystalline degradation products released from bone grafts and different types of bone cement. Folia Microbiol. 65 (3), 475–482. 10.1007/s12223-019-00752-w 31654320

[B104] Kneipp J. Kneipp H. Wittig B. Kneipp K. (2010). Novel optical nanosensors for probing and imaging live cells. Nanomedicine Nanotechnol. Biol. Med. 6 (2), 214–226. 10.1016/j.nano.2009.07.009 19699322

[B105] Koch-Weser J. (1972). Serum drug concentrations as therapeutic guides. N. Engl. J. Med. 287 (5), 227–231. 10.1056/NEJM197208032870505 5037497

[B106] Kono K. (2001). Thermosensitive polymer-modified liposomes. Adv. Drug Delivery Reviews 53 (3), 307–319. 10.1016/s0169-409x(01)00204-6 11744174

[B107] Kousi M. Kalogiouri N. P. Samanidou V. F. (2025). Recent advances in bioanalysis of cephalosporins toward green sample preparation. J. Sep. Sci. 48 (2), e70096. 10.1002/jssc.70096 39973572 PMC11840664

[B108] Krishnamurthy V. M. Semetey V. Bracher P. J. Shen N. Whitesides G. M. (2007). Dependence of effective molarity on linker length for an intramolecular protein− ligand system. J. Am. Chem. Soc. 129 (5), 1312–1320. 10.1021/ja066780e 17263415 PMC2535942

[B109] Kristoffersson A. David-Pierson P. Parrott N. Kuhlmann O. Lave T. Friberg L. (2016). Simulation-based evaluation of PK/PD indices for meropenem across patient groups and experimental designs. Pharm. Research 33 (5), 1115–1125. 10.1007/s11095-016-1856-x 26786016

[B110] Kumps A. H. (1982). Therapeutic drug monitoring: a comprehensive and critical review of analytical methods for anticonvulsive drugs. J. Neurology 228 (1), 1–16. 10.1007/BF00313405 6184454

[B111] Landi F. Bandettini R. Rotulo G. A. Mesini A. Saffioti C. Amoroso L. (2020). Resistance to antibiotics of uropathogen bacteria isolated from urine and blood in pediatric cancer patients: a single center, 12-year study. Pediatr. Infect. Dis. J. 39 (12), 1106–1110. 10.1097/INF.0000000000002854 33021597

[B112] Lee H. Choi T. K. Lee Y. B. Cho H. R. Ghaffari R. Wang L. (2016). A graphene-based electrochemical device with thermoresponsive microneedles for diabetes monitoring and therapy. Nat. Nanotechnology 11 (6), 566–572. 10.1038/nnano.2016.38 26999482

[B113] Legg A. Carmichael S. Chai M. G. Roberts J. A. Cotta M. O. (2023). Beta-lactam dose optimisation in the intensive care unit: targets, therapeutic drug monitoring and toxicity. Antibiotics 12 (5), 870. 10.3390/antibiotics12050870 37237773 PMC10215385

[B114] Lepak A. J. Zhao M. Marchillo K. VanHecker J. (2017). Andes DR: *In vivo* pharmacodynamic evaluation of omadacycline (PTK 0796) against Streptococcus pneumoniae in the murine pneumonia model. Antimicrob. Agents Chemother. 61 (5), e02368–e02416. 10.1128/AAC.02368-16 28193651 PMC5404567

[B115] Lepak A. J. Zhao M. Marchillo K. VanHecker J. (2019). Andes DR: *In vivo* pharmacodynamics of omadacycline against Staphylococcus aureus in the neutropenic murine thigh infection model. Antimicrob. Agents Chemother. 63 (7), e00624–e00719. 10.1128/AAC.00624-19 31036691 PMC6591633

[B116] Leung K. S.-Y. Fong B. M.-W. (2014). LC–MS/MS in the routine clinical laboratory: has its time come? Anal. Bioanalytical Chemistry 406 (9), 2289–2301. 10.1007/s00216-013-7542-5 24337187

[B117] Li C. G. Lee C. Y. Lee K. Jung H. (2013). An optimized hollow microneedle for minimally invasive blood extraction. Biomed. Microdevices 15 (1), 17–25. 10.1007/s10544-012-9683-2 22833155

[B118] Li X. Yang F. Wong J. X. Yu H.-Z. (2017). Integrated smartphone-app-chip system for on-site parts-per-billion-level colorimetric quantitation of aflatoxins. Anal. Chemistry 89 (17), 8908–8916. 10.1021/acs.analchem.7b01379 28719742

[B119] Li Z. Chen H. Wang P. (2019). Lateral flow assay ruler for quantitative and rapid point-of-care testing. Analyst 144 (10), 3314–3322. 10.1039/c9an00374f 30968883 PMC7169999

[B120] Li R. Wang T. Gong L. Dong J. Xiao N. Yang X. (2020). Enhance the effectiveness of clinical laboratory critical values initiative notification by implementing a closed‐loop system: a five‐year retrospective observational study. J. Clin. Laboratory Analysis 34 (2), e23038. 10.1002/jcla.23038 31531906 PMC7031628

[B121] Li W. Fu Y. Picard F. (2024a). Inclusion of dilution quality control samples in quantitative LC-MS bioanalysis. In., vol. Bioanalysis (East Hanover, NJ, United States: Taylor & Francis), 16: 923–925. 10.1080/17576180.2024.2352253 39324496 PMC11486123

[B122] Li Q. Y. Tang B. H. Wu Y. E. Yao B. F. Zhang W. Zheng Y. (2024b). Machine learning: a new approach for dose individualization. Clin. Pharmacol. and Ther. 115 (4), 727–744. 10.1002/cpt.3049 37713106

[B123] Lin Z. Chen D.-y. Zhu Y.-W. Jiang Z.-l. Cui K. Zhang S. (2021). Population pharmacokinetic modeling and clinical application of vancomycin in Chinese patients hospitalized in intensive care units. Sci. Rep. 11 (1), 2670. 10.1038/s41598-021-82312-2 33514803 PMC7846798

[B124] Liu V. X. Fielding-Singh V. Greene J. D. Baker J. M. Iwashyna T. J. Bhattacharya J. (2017). The timing of early antibiotics and hospital mortality in sepsis. Am. Journal Respiratory Critical Care Medicine 196 (7), 856–863. 10.1164/rccm.201609-1848OC 28345952 PMC5649973

[B125] Liu Y. Zhan L. Qin Z. Sackrison J. Bischof J. C. (2021). Ultrasensitive and highly specific lateral flow assays for point-of-care diagnosis. ACS Nano 15 (3), 3593–3611. 10.1021/acsnano.0c10035 33607867

[B126] Loll P. J. Kaplan J. Selinsky B. S. Axelsen P. H. (1999). Vancomycin binding to low-affinity ligands: delineating a minimum set of interactions necessary for high-affinity binding. J. Medicinal Chemistry 42 (22), 4714–4719. 10.1021/jm990361t 10579833

[B127] Ma X. Ahadian S. Liu S. Zhang J. Liu S. Cao T. (2021). Smart contact lenses for biosensing applications. Adv. Intell. Syst. 3 (5), 2000263. 10.1002/aisy.202170047

[B128] Mabilat C. Gros M. F. Nicolau D. Mouton J. W. Textoris J. Roberts J. A. (2020). Diagnostic and medical needs for therapeutic drug monitoring of antibiotics. Eur. J. Clin. Microbiol. and Infect. Dis. 39 (5), 791–797. 10.1007/s10096-019-03769-8 31828686 PMC7182631

[B129] Maekawa-Matsuura M. Fujieda K. Maekawa Y. Nishimura T. Nagase K. Kanazawa H. (2019). LAT1-targeting thermoresponsive liposomes for effective cellular uptake by cancer cells. ACS Omega 4 (4), 6443–6451. 10.1021/acsomega.9b00216

[B130] Magréault S. Jaureguy F. Zahar J.-R. Méchaï F. Toinon D. Cohen Y. (2022). Automated HPLC-MS/MS assay for the simultaneous determination of ten plasma antibiotic concentrations. J. Chromatogr. B 1211, 123496. 10.1016/j.jchromb.2022.123496 36244237

[B131] Maier D. Laubender E. Basavanna A. Schumann S. Güder F. Urban G. A. (2019). Toward continuous monitoring of breath biochemistry: a paper-based wearable sensor for real-time hydrogen peroxide measurement in simulated breath. ACS Sensors 4 (11), 2945–2951. 10.1021/acssensors.9b01403 31610653 PMC6879172

[B132] Majors R. E. (1980). Multidimensional high performance liquid chromatography. J. Chromatogr. Sci. 18 (10), 571–579. 10.1093/chromsci/18.10.571 7451635

[B133] Malmstadt N. Yager P. Hoffman A. S. Stayton P. S. (2003). A smart microfluidic affinity chromatography matrix composed of poly (N-isopropylacrylamide)-coated beads. Anal. Chemistry 75 (13), 2943–2949. 10.1021/ac034274r 12964737

[B134] Mansoor I. Liu Y. Häfeli U. Stoeber B. (2013). Arrays of hollow out-of-plane microneedles made by metal electrodeposition onto solvent cast conductive polymer structures. J. Micromechanics Microengineering 23 (8), 085011. 10.1088/0960-1317/23/8/085011

[B135] Mansoor I. Lai J. Ranamukhaarachchi S. Schmitt V. Lambert D. Dutz J. (2015). A microneedle-based method for the characterization of diffusion in skin tissue using doxorubicin as a model drug. Biomed. Microdevices 17 (3), 61. 10.1007/s10544-015-9967-4 26009275

[B136] Masharina A. Reymond L. Maurel D. Umezawa K. Johnsson K. (2012). A fluorescent sensor for GABA and synthetic GABAB receptor ligands. J. Am. Chem. Soc. 134 (46), 19026–19034. 10.1021/ja306320s 23095089

[B137] Masuda T. Terasaki A. Akimoto A. M. Nagase K. Okano T. Yoshida R. (2015a). Control of swelling–deswelling behavior of a self-oscillating gel by designing the chemical structure. RSC Adv. 5 (8), 5781–5787. 10.1039/c4ra10675j

[B138] Masuda T. Akimoto A. M. Nagase K. Okano T. Yoshida R. (2015b). Design of self-oscillating polymer brushes and control of the dynamic behaviors. Chem. Mater. 27 (21), 7395–7402. 10.1021/acs.chemmater.5b03228

[B139] Matsumoto K. Oda K. Shoji K. Hanai Y. Takahashi Y. Fujii S. (2022). Clinical practice guidelines for therapeutic drug monitoring of vancomycin in the framework of model-informed precision dosing: a consensus review by the Japanese society of chemotherapy and the Japanese society of therapeutic drug monitoring. Pharmaceutics 14 (3), 489. 10.3390/pharmaceutics14030489 35335866 PMC8955715

[B140] Matsuura K. Utoh R. Nagase K. Okano T. (2014). Cell sheet approach for tissue engineering and regenerative medicine. J. Control. Release 190, 228–239. 10.1016/j.jconrel.2014.05.024 24858800

[B141] Matsuura M. Ohshima M. Hiruta Y. Nishimura T. Nagase K. Kanazawa H. (2018). LAT1-targeting thermoresponsive fluorescent polymer probes for cancer cell imaging. Int. Journal Molecular Sciences 19 (6), 1646. 10.3390/ijms19061646 29865203 PMC6032285

[B142] Miyamoto D. Tang Z. Takarada T. Maeda M. (2007). Turbidimetric detection of ATP using polymeric micelles and DNA aptamers. Chem. Commun. (45), 4743–4745. 10.1039/b709775a 18004427

[B143] Moorthy G. S. Vedar C. Zane N. R. Downes K. J. Prodell J. L. DiLiberto M. A. (2020). Development and validation of a volumetric absorptive microsampling-liquid chromatography mass spectrometry method for the analysis of cefepime in human whole blood: application to pediatric pharmacokinetic study. J. Pharmaceutical Biomedical Analysis 179, 113002. 10.1016/j.jpba.2019.113002 31785929 PMC6943186

[B144] Mori T. Maeda M. (2004). Temperature-responsive formation of colloidal nanoparticles from poly (N-isopropylacrylamide) grafted with single-stranded DNA. Langmuir 20 (2), 313–319. 10.1021/la0356194 15743072

[B145] Mori T. Umeno D. Maeda M. (2001). Sequence‐specific affinity precipitation of oligonucleotide using poly (N‐isopropylacrylamide)–oligonucleotide conjugate. Biotechnol. Bioengineering 72 (3), 261–268. 10.1002/1097-0290(20010205)72:3<261::aid-bit2>3.0.co;2-7 11135195

[B146] Moskovits M. (2005). Surface‐enhanced raman spectroscopy: a brief retrospective. J. Raman Spectrosc. Int. J. Orig. Work All Aspects Raman Spectrosc. Incl. High. Order Process. Also Brillouin Rayleigh Scatt. 36 (6‐7), 485–496. 10.1002/jrs.1362

[B147] Mukerjee E. Collins S. Isseroff R. Smith R. (2004). Microneedle array for transdermal biological fluid extraction and *in situ* analysis. Sensors Actuators A Phys. 114 (2-3), 267–275. 10.1016/j.sna.2003.11.008

[B148] Nagase K. (2021). Thermoresponsive interfaces obtained using poly (N-isopropylacrylamide)-based copolymer for bioseparation and tissue engineering applications. Adv. Colloid Interface Sci. 295, 102487. 10.1016/j.cis.2021.102487 34314989

[B149] Nagase K. Kanazawa H. (2020). Temperature-responsive chromatography for bioseparations: a review. Anal. Chim. Acta 1138, 191–212. 10.1016/j.aca.2020.07.075 33161981

[B150] Nagase K. Okano T. (2016). Thermoresponsive-polymer-based materials for temperature-modulated bioanalysis and bioseparations. J. Mater. Chem. B 4 (39), 6381–6397. 10.1039/c6tb01003b 32263447

[B151] Nagase K. Kobayashi J. Kikuchi A. Akiyama Y. Kanazawa H. Okano T. (2007). Interfacial property modulation of thermoresponsive polymer brush surfaces and their interaction with biomolecules. Langmuir 23 (18), 9409–9415. 10.1021/la700956b 17683149

[B152] Nagase K. Kobayashi J. Kikuchi A. Akiyama Y. Kanazawa H. Okano T. (2008). Effects of graft densities and chain lengths on separation of bioactive compounds by nanolayered thermoresponsive polymer brush surfaces. Langmuir 24 (2), 511–517. 10.1021/la701839s 18085801

[B153] Nagase K. Kobayashi J. Okano T. (2009). Temperature-responsive intelligent interfaces for biomolecular separation and cell sheet engineering. J. R. Soc. Interface 6 (Suppl. l_3), S293–S309. 10.1098/rsif.2008.0499.focus 19324682 PMC2690096

[B154] Nagase K. Kimura A. Shimizu T. Matsuura K. Yamato M. Takeda N. (2012). Dynamically cell separating thermo-functional biointerfaces with densely packed polymer brushes. J. Mater. Chem. 22 (37), 19514–19522. 10.1039/c2jm31797d

[B155] Nagase K. Onuma T. Yamato M. Takeda N. Okano T. (2015). Enhanced wettability changes by synergistic effect of micro/nanoimprinted substrates and grafted thermoresponsive polymer brushes. Macromol. Rapid Communications 36 (22), 1965–1970. 10.1002/marc.201500393 26375171

[B156] Nagase K. Sakurada Y. Onizuka S. Iwata T. Yamato M. Takeda N. (2017a). Thermoresponsive polymer-modified microfibers for cell separations. Acta Biomater. 53, 81–92. 10.1016/j.actbio.2017.02.033 28219809

[B157] Nagase K. Shukuwa R. Onuma T. Yamato M. Takeda N. Okano T. (2017b). Micro/nano-imprinted substrates grafted with a thermoresponsive polymer for thermally modulated cell separation. J. Mater. Chem. B 5 (30), 5924–5930. 10.1039/c7tb01251a 32264348

[B158] Nagase K. Nagumo Y. Kim M. Kim H. J. Kyung H. W. Chung H. J. (2017c). Local release of VEGF using fiber mats enables effective transplantation of layered cardiomyocyte sheets. Macromol. Biosci. 17 (8), 1700073. 10.1002/mabi.201700073 28547766

[B159] Nagase K. Okano T. Kanazawa H. (2018a). Poly (N-isopropylacrylamide) based thermoresponsive polymer brushes for bioseparation, cellular tissue fabrication, and nano actuators. Nano-Structures and Nano-Objects 16, 9–23. 10.1016/j.nanoso.2018.03.010

[B160] Nagase K. Yamato M. Kanazawa H. Okano T. (2018b). Poly (N-isopropylacrylamide)-based thermoresponsive surfaces provide new types of biomedical applications. Biomaterials 153, 27–48. 10.1016/j.biomaterials.2017.10.026 29096399

[B161] Nagase K. Hasegawa M. Ayano E. Maitani Y. Kanazawa H. (2019). Effect of polymer phase transition behavior on temperature-responsive polymer-modified liposomes for siRNA transfection. Int. Journal Molecular Sciences 20 (2), 430. 10.3390/ijms20020430 30669495 PMC6358841

[B162] Nagase K. Shukuwa R. Takahashi H. Takeda N. Okano T. (2020). Enhanced mechanical properties and cell separation with thermal control of PIPAAm-brushed polymer-blend microfibers. J. Mater. Chem. B 8 (28), 6017–6026. 10.1039/d0tb00972e 32573640

[B163] Nagase K. Inoue S. Inoue M. Kanazawa H. (2022a). Two-dimensional temperature-responsive chromatography using a poly (N-isopropylacrylamide) brush-modified stationary phase for effective therapeutic drug monitoring. Sci. Reports 12 (1), 2653. 10.1038/s41598-022-06638-1 PMC8850448 35173260

[B164] Nagase K. Takagi H. Nakada H. Ishikawa H. Nagata Y. Aomori T. (2022b). Chromatography columns packed with thermoresponsive-cationic-polymer-modified beads for therapeutic drug monitoring. Sci. Reports 12 (1), 12847. 10.1038/s41598-022-16928-3 PMC9329465 35896711

[B165] Naghavi M. Vollset S. E. Ikuta K. S. Swetschinski L. R. Gray A. P. Wool E. E. (2024). Global burden of bacterial antimicrobial resistance 1990–2021: a systematic analysis with forecasts to 2050. Lancet 404 (10459), 1199–1226. 10.1016/s0140-6736(24)01867-1 39299261 PMC11718157

[B166] Najjar T. A. Al-Dhuwailie A. A. Tekle A. (1995). Comparison of high-performance liquid chromatography with fluorescence polarization immunoassay for the analysis of vancomycin in patients with chronic renal failure. J. Chromatogr. B Biomed. Sci. Appl. 672 (2), 295–299. 10.1016/0378-4347(95)00220-d 8581137

[B167] Nakao M. Kim K. Nagase K. Grainger D. W. Kanazawa H. Okano T. (2019). Phenotypic traits of mesenchymal stem cell sheets fabricated by temperature-responsive cell culture plate: structural characteristics of MSC sheets. Stem Cell Res. and Ther. 10 (1), 353. 10.1186/s13287-019-1431-6 31779694 PMC6883536

[B168] Nakayama M. Okano T. (2011). Multi-targeting cancer chemotherapy using temperature-responsive drug carrier systems. React. Funct. Polym. 71 (3), 235–244. 10.1016/j.reactfunctpolym.2010.08.006

[B169] Nemoto R. Fujieda K. Hiruta Y. Hishida M. Ayano E. Maitani Y. (2019). Liposomes with temperature-responsive reversible surface properties. Colloids Surfaces B Biointerfaces 176, 309–316. 10.1016/j.colsurfb.2019.01.007 30641302

[B170] Nguyen P. Q. Soenksen L. R. Donghia N. M. Angenent-Mari N. M. de Puig H. Huang A. (2021). Wearable materials with embedded synthetic biology sensors for biomolecule detection. Nat. Biotechnol. 39 (11), 1366–1374. 10.1038/s41587-021-00950-3 34183860

[B171] Nielsen E. I. Friberg L. E. (2013). Pharmacokinetic-pharmacodynamic modeling of antibacterial drugs. Pharmacol. Reviews 65 (3), 1053–1090. 10.1124/pr.111.005769 23803529

[B172] Nigo M. Tran H. T. N. Xie Z. Feng H. Mao B. Rasmy L. (2022). PK-RNN-V E: a deep learning model approach to vancomycin therapeutic drug monitoring using electronic health record data. J. Biomed. Inf. 133, 104166. 10.1016/j.jbi.2022.104166 35985620

[B173] Niu A. Yan X. Wang L. Min Y. Hu C. (2013). Utility and necessity of repeat testing of critical values in the clinical chemistry laboratory. PLoS One 8 (11), e80663. 10.1371/journal.pone.0080663 24260448 PMC3834106

[B174] Nordmann P. Picazo J. J. Mutters R. Korten V. Quintana A. Laeuffer J. M. (2011). Comparative activity of carbapenem testing: the COMPACT study. J. Antimicrobial Chemotherapy 66 (5), 1070–1078. 10.1093/jac/dkr056 21393160

[B175] Nuntawong P. Putalun W. Tanaka H. Morimoto S. Sakamoto S. (2022). Lateral flow immunoassay for small-molecules detection in phytoproducts: a review. J. Nat. Med. 76 (3), 521–545. 10.1007/s11418-022-01605-6 35171397 PMC9165253

[B176] Ohmori T. Suzuki A. Niwa T. Ushikoshi H. Shirai K. Yoshida S. (2011). Simultaneous determination of eight β-lactam antibiotics in human serum by liquid chromatography–tandem mass spectrometry. J. Chromatogr. B 879 (15-16), 1038–1042. 10.1016/j.jchromb.2011.03.001 21459052

[B177] Okada A. Kariya M. Irie K. Okada Y. Hiramoto N. Hashimoto H. (2018). Population pharmacokinetics of vancomycin in patients undergoing allogeneic hematopoietic stem‐cell transplantation. J. Clin. Pharmacol. 58 (9), 1140–1149. 10.1002/jcph.1106 29762865

[B178] Onufrak N. J. Forrest A. Gonzalez D. (2016). Pharmacokinetic and pharmacodynamic principles of anti-infective dosing. Clin. Therapeutics 38 (9), 1930–1947. 10.1016/j.clinthera.2016.06.015 27449411 PMC5039113

[B179] Pai M. Moffat K. A. Plumhoff E. Hayward C. P. (2011). Critical values in the coagulation laboratory: results of a survey of the north American specialized coagulation laboratory association. Am. Journal Clinical Pathology 136 (6), 836–841. 10.1309/AJCP8O8GIPPPNUSH 22095367

[B180] Park J.-H. Park E.-K. Cho Y. K. Shin I.-S. Lee H. (2022). Normalizing the optical signal enables robust assays with lateral flow biosensors. Acs Omega 7 (21), 17723–17731. 10.1021/acsomega.2c00793 35664567 PMC9161384

[B181] Pea F. (2020). Teicoplanin and therapeutic drug monitoring: an update for optimal use in different patient populations. J. Infect. Chemother. 26 (9), 900–907. 10.1016/j.jiac.2020.06.006 32624339

[B182] Pea F. Viale P. Furlanut M. (2005). Antimicrobial therapy in critically ill patients: a review of pathophysiological conditions responsible for altered disposition and pharmacokinetic variability. Clin. Pharmacokinetics 44 (10), 1009–1034. 10.2165/00003088-200544100-00002 16176116

[B89] Pfaller M. A. Krogstad D. J. Granich G. G. Murray P. R. (1984). Laboratory evaluation of five assay methods for vancomycin: bioassay, high-pressure liquid chromatography, fluorescence polarization immunoassay, radioimmunoassay, and fluorescence immunoassay. J. Clin. Microbiol. 20 (3), 311–316. 10.1128/JCM.20.3.311-316.1984 6386852 PMC271319

[B183] Pollock N. R. Rolland J. P. Kumar S. Beattie P. D. Jain S. Noubary F. (2012). A paper-based multiplexed transaminase test for low-cost, point-of-care liver function testing. Sci. Translational Medicine 4 (152), 152ra129. 10.1126/scitranslmed.3003981 22993296 PMC3624093

[B184] Prausnitz M. R. Langer R. (2008). Transdermal drug delivery. Nat. Biotechnology 26 (11), 1261–1268. 10.1038/nbt.1504 18997767 PMC2700785

[B185] Qian X. Peng X.-H. Ansari D. O. Yin-Goen Q. Chen G. Z. Shin D. M. (2008). *In vivo* tumor targeting and spectroscopic detection with surface-enhanced raman nanoparticle tags. Nat. Biotechnology 26 (1), 83–90. 10.1038/nbt1377 18157119

[B186] Rahimpour E. Khoubnasabjafari M. Hosseini M. B. Jouyban A. (2021). Copper nanocluster-based sensor for determination of vancomycin in exhaled breath condensate: a synchronous fluorescence spectroscopy. J. Pharmaceutical Biomedical Analysis 196, 113906. 10.1016/j.jpba.2021.113906 33486448

[B187] Ranamukhaarachchi S. A. Padeste C. Dübner M. Häfeli U. O. Stoeber B. Cadarso V. J. (2016a). Integrated hollow microneedle-optofluidic biosensor for therapeutic drug monitoring in sub-nanoliter volumes. Sci. Reports 6 (1), 29075. 10.1038/srep29075 27380889 PMC4933911

[B188] Ranamukhaarachchi S. A. Schneider T. Lehnert S. Sprenger L. Campbell J. R. Mansoor I. (2016b). Development and validation of an artificial mechanical skin model for the study of interactions between skin and microneedles. Macromol. Mater. Eng. 301 (3), 306–314. 10.1002/mame.201500320

[B189] Rao G. G. Konicki R. Cattaneo D. Alffenaar J.-W. Marriott D. J. Neely M. (2020). Therapeutic drug monitoring can improve linezolid dosing regimens in current clinical practice: a review of linezolid pharmacokinetics and pharmacodynamics. Ther. Drug Monitoring 42 (1), 83–92. 10.1097/FTD.0000000000000710 31652190

[B190] Rawson T. M. Wilson R. C. O’Hare D. Herrero P. Kambugu A. Lamorde M. (2021). Optimizing antimicrobial use: challenges, advances and opportunities. Nat. Rev. Microbiol. 19 (12), 747–758. 10.1038/s41579-021-00578-9 34158654

[B191] Reed M. (2000). Optimal antibiotic dosing. The pharmacokinetic-pharmacodynamic interface. Postgrad. Medicine 108 (7 Suppl. Contemporaty), 17–24. 10.3810/pgm.12.2000.suppl10.52 19667545

[B192] Rees V. E. Bulitta J. B. Oliver A. Tsuji B. T. Rayner C. R. Nation R. L. (2016). Resistance suppression by high-intensity, short-duration aminoglycoside exposure against hypermutable and non-hypermutable *Pseudomonas aeruginosa* . J. Antimicrob. Chemother. 71 (11), 3157–3167. 10.1093/jac/dkw297 27521357 PMC5079302

[B193] Revilla N. Martín‐Suárez A. Pérez M. P. González F. M. Fernández de Gatta M. M. (2010). Vancomycin dosing assessment in intensive care unit patients based on a population pharmacokinetic/pharmacodynamic simulation. Br. Journal Clinical Pharmacology 70 (2), 201–212. 10.1111/j.1365-2125.2010.03679.x 20653673 PMC2911550

[B194] Riezk A. Vasikasin V. Wilson R. C. Rawson T. M. Cass A. E. Holmes A. H. (2025). Development of a novel quantitative lateral flow assay for vancomycin for therapeutic drug monitoring. Sci. Rep. 15 (1), 24398. 10.1038/s41598-025-09145-1 40628844 PMC12238248

[B195] Rigo-Bonnin R. Ribera A. Arbiol-Roca A. Cobo-Sacristán S. Padullés A. Murillo Ò. (2017). Development and validation of a measurement procedure based on ultra-high performance liquid chromatography-tandem mass spectrometry for simultaneous measurement of β-lactam antibiotic concentration in human plasma. Clin. Chimica Acta 468, 215–224. 10.1016/j.cca.2017.03.009 28288784

[B196] Roberts J. A. Ulldemolins M. Roberts M. S. McWhinney B. Ungerer J. Paterson D. L. (2010). Therapeutic drug monitoring of β-lactams in critically ill patients: proof of concept. Int. Journal Antimicrobial Agents 36 (4), 332–339. 10.1016/j.ijantimicag.2010.06.008 20685085

[B197] Roberts J. A. Paul S. K. Akova M. Bassetti M. De Waele J. J. Dimopoulos G. (2014a). DALI: defining antibiotic levels in intensive care unit patients: are current β-lactam antibiotic doses sufficient for critically ill patients? Clin. Infectious Diseases 58 (8), 1072–1083. 10.1093/cid/ciu027 24429437

[B198] Roberts J. A. Aziz MMHA Lipman J. Mouton J. W. Vinks A. A. Felton T. W. (2014b). Challenges and potential solutions–individualised antibiotic dosing at the bedside for critically ill patients: a structured review. Lancet Infect. Dis. 14 (6), 498. 10.1016/S1473-3099(14)70036-2 24768475 PMC4181663

[B199] Roberts J. A. Abdul-Aziz M.-H. Davis J. S. Dulhunty J. M. Cotta M. O. Myburgh J. (2016). Continuous *versus* intermittent β-lactam infusion in severe sepsis. A meta-analysis of individual patient data from randomized trials. Am. Journal Respiratory Critical Care Medicine 194 (6), 681–691. 10.1164/rccm.201601-0024OC 26974879

[B200] Rodvold K. A. Pai M. P. (2019). Pharmacokinetics and pharmacodynamics of oral and intravenous omadacycline. Clin. Infect. Dis. 69 (Suppl. ment_1), S16–S22. 10.1093/cid/ciz309 31367744 PMC6669312

[B201] Rose W. Volk C. Dilworth T. J. Sakoulas G. (2022). Approaching 65 years: is it time to consider retirement of vancomycin for treating methicillin-resistant staphylococcus aureus endovascular infections? In: Open Forum Infectious Diseases: Oxford University Press US; 2022: ofac137. 10.1093/ofid/ofac137 PMC9043000 35493116

[B202] Roy D. Brooks W. L. Sumerlin B. S. (2013). New directions in thermoresponsive polymers. Chem. Soc. Rev. 42 (17), 7214–7243. 10.1039/c3cs35499g 23450220

[B203] Ruppert C. Phogat N. Laufer S. Kohl M. Deigner H.-P. (2019). A smartphone readout system for gold nanoparticle-based lateral flow assays: application to monitoring of digoxigenin. Microchim. Acta 186 (2), 119. 10.1007/s00604-018-3195-6 30661134 PMC6339659

[B204] Rybak M. J. (2006). The pharmacokinetic and pharmacodynamic properties of vancomycin. Clin. Infect. Dis. 42 (Suppl. ment_1), S35–S39. 10.1086/491712 16323118

[B205] Rybak M. J. Le J. Lodise T. P. Levine D. P. Bradley J. S. Liu C. (2020). Therapeutic monitoring of vancomycin for serious methicillin-resistant *Staphylococcus aureus* infections: a revised consensus guideline and review by the American society of health-system pharmacists, the infectious diseases society of America, the pediatric infectious diseases society, and the society of infectious diseases pharmacists. Am. J. Health-System Pharm. 77 (11), 835–864. 10.1093/ajhp/zxaa036 32191793

[B206] Saisin L. Amarit R. Somboonkaew A. Gajanandana O. Himananto O. Sutapun B. (2018). Significant sensitivity improvement for camera-based lateral flow immunoassay readers. Sensors 18 (11), 4026. 10.3390/s18114026 30463191 PMC6263405

[B207] Saito K. Hatsugai N. Horikawa K. Kobayashi K. Matsu-Ura T. Mikoshiba K. (2010). Auto-luminescent genetically-encoded ratiometric indicator for real-time Ca2+ imaging at the single cell level. PLoS One 5 (4), e9935. 10.1371/journal.pone.0009935 20376337 PMC2848576

[B208] Sajid M. Kawde A.-N. Daud M. (2015). Designs, formats and applications of lateral flow assay: a literature review. J. Saudi Chem. Soc. 19 (6), 689–705. 10.1016/j.jscs.2014.09.001

[B209] Scherr T. F. Gupta S. Wright D. W. Haselton F. R. (2016). Mobile phone imaging and cloud-based analysis for standardized malaria detection and reporting. Sci. Reports 6 (1), 28645. 10.1038/srep28645 27345590 PMC4921854

[B210] Schlücker S. (2014). Surface‐enhanced raman spectroscopy: concepts and chemical applications. Angew. Chem. Int. Ed. 53 (19), 4756–4795. 10.1002/anie.201205748 24711218

[B211] Seger C. Salzmann L. (2020). After another decade: LC–MS/MS became routine in clinical diagnostics. Clin. Biochemistry 82, 2–11. 10.1016/j.clinbiochem.2020.03.004 32188572

[B212] Sempionatto J. R. Lin M. Yin L. De la Paz E. Pei K. Sonsa-Ard T. (2021). An epidermal patch for the simultaneous monitoring of haemodynamic and metabolic biomarkers. Nat. Biomed. Eng. 5 (7), 737–748. 10.1038/s41551-021-00685-1 33589782

[B213] Sergi C. (2018). Promptly reporting of critical laboratory values in pediatrics: a work in progress. World Journal Clinical Pediatrics 7 (5), 105–110. 10.5409/wjcp.v7.i5.105 30479975 PMC6242778

[B214] Šestáková N. Theurillat R. Sendi P. Thormann W. (2017). Monitoring of cefepime in human serum and plasma by micellar electrokinetic capillary chromatography: improvement of sample preparation and validation by liquid chromatography coupled to mass spectrometry. J. Separation Science 40 (8), 1805–1814. 10.1002/jssc.201601446 28217952

[B215] Shipkova M. Jamoussi H. (2022). Therapeutic drug monitoring of antibiotic drugs: the role of the clinical laboratory. Ther. Drug Monit. 44 (1), 32–49. 10.1097/ftd.0000000000000934 34726200

[B216] Sibieude E. Khandelwal A. Hesthaven J. S. Girard P. Terranova N. (2021). Fast screening of covariates in population models empowered by machine learning. J. Pharmacokinetics Pharmacodynamics 48 (4), 597–609. 10.1007/s10928-021-09757-w PMC8225540 34019213

[B217] Sime F. B. Roberts M. S. Tiong I. S. Gardner J. H. Lehman S. Peake S. L. (2015). Can therapeutic drug monitoring optimize exposure to piperacillin in febrile neutropenic patients with haematological malignancies? A randomized controlled trial. J. Antimicrob. Chemother. 70 (8), 2369–2375. 10.1093/jac/dkv123 25953805

[B218] Sivamani R. K. Stoeber B. Wu G. C. Zhai H. Liepmann D. Maibach H. (2005). Clinical microneedle injection of methyl nicotinate: stratum corneum penetration. Skin Res. Technol. 11 (2), 152–156. 10.1111/j.1600-0846.2005.00107.x 15807814

[B219] Skhirtladze K. Hutschala D. Fleck T. Thalhammer F. Ehrlich M. Vukovich T. (2006). Impaired target site penetration of vancomycin in diabetic patients following cardiac surgery. Antimicrob. Agents Chemotherapy 50 (4), 1372–1375. 10.1128/AAC.50.4.1372-1375.2006 16569854 PMC1426928

[B220] Sou T. Hansen J. Liepinsh E. Backlund M. Ercan O. Grinberga S. (2021). Model‐informed drug development for antimicrobials: translational PK and PK/PD modeling to predict an efficacious human dose for apramycin. Clin. Pharmacol. and Ther. 109 (4), 1063–1073. 10.1002/cpt.2104 33150591 PMC8048880

[B221] Stoeber B. Liepmann D. (2005). Arrays of hollow out-of-plane microneedles for drug delivery. J. Microelectromechanical Systems 14 (3), 472–479. 10.1109/jmems.2005.844843

[B222] Stosch R. Henrion A. Schiel D. Güttler B. (2005). Surface-enhanced raman scattering based approach for quantitative determination of creatinine in human serum. Anal. Chemistry 77 (22), 7386–7392. 10.1021/ac0511647 16285690

[B223] Strambini L. Longo A. Scarano S. Prescimone T. Palchetti I. Minunni M. (2015). Self-powered microneedle-based biosensors for pain-free high-accuracy measurement of glycaemia in interstitial fluid. Biosens. Bioelectron. 66, 162–168. 10.1016/j.bios.2014.11.010 25601169

[B224] Suhang G. Ren Z. Xudong F. Ruoying Z. Xinjun C. Jie J. (2024). Development, validation, and clinical application of a UPLC-MS/MS method for omadacycline determination in human serum. J. Pharmacol. Toxicol. Methods 127, 107503. 10.1016/j.vascn.2024.107503 38574874

[B225] Sun F. Hung H.-C. Sinclair A. Zhang P. Bai T. Galvan D. D. (2016). Hierarchical zwitterionic modification of a SERS substrate enables real-time drug monitoring in blood plasma. Nat. Communications 7 (1), 13437. 10.1038/ncomms13437 27834380 PMC5114600

[B226] Taccone F. S. Laterre P.-F. Dugernier T. Spapen H. Delattre I. Wittebole X. (2010). Insufficient β-lactam concentrations in the early phase of severe sepsis and septic shock. Crit. Care 14 (4), R126. 10.1186/cc9091 20594297 PMC2945087

[B227] Tang Z. Takarada T. Maeda M. (2018). Non-cross-linking aggregation of DNA-carrying polymer micelles triggered by duplex formation. Langmuir 34 (49), 14899–14910. 10.1021/acs.langmuir.8b01840 30086233

[B228] Taylor R. D. MacCoss M. Lawson A. D. (2014). Rings in drugs: miniperspective. J. Medicinal Chemistry 57 (14), 5845–5859. 10.1021/jm4017625 24471928

[B229] Tietjen A. K. Kroemer N. Cattaneo D. Baldelli S. Wicha S. G. (2022). Population pharmacokinetics and target attainment analysis of linezolid in multidrug‐resistant tuberculosis patients. Br. J. Clin. Pharmacol. 88 (4), 1835–1844. 10.1111/bcp.15102 34622478

[B230] Topol E. J. (2019). High-performance medicine: the convergence of human and artificial intelligence. Nat. Medicine 25 (1), 44–56. 10.1038/s41591-018-0300-7 30617339

[B231] Trittler R. Ehrlich M. Galla T. Horch R. Kümmerer K. (2002). New and rapid fully automated method for determination of tazobactam and piperacillin in fatty tissue and serum by column-switching liquid chromatography. J. Chromatogr. B 775 (2), 127–132. 10.1016/s1570-0232(02)00298-2 12113978

[B232] Turner A. P. (2013). Biosensors: sense and sensibility. Chem. Soc. Rev. 42 (8), 3184–3196. 10.1039/c3cs35528d 23420144

[B233] Tzanis E. Manley A. Villano S. Tanaka S. K. Bai S. Loh E. (2017). Effect of food on the bioavailability of omadacycline in healthy participants. J. Clin. Pharmacol. 57 (3), 321–327. 10.1002/jcph.814 27539539 PMC5324643

[B234] Urusov A. E. Zherdev A. V. Dzantiev B. B. (2019). Towards lateral flow quantitative assays: detection approaches. Biosensors 9 (3), 89. 10.3390/bios9030089 31319629 PMC6784366

[B235] van der Gugten J. G. (2020). Tandem mass spectrometry in the clinical laboratory: a tutorial overview. Clin. Mass Spectrom. 15, 36–43. 10.1016/j.clinms.2019.09.002

[B236] Vandenberg O. Durand G. Hallin M. Diefenbach A. Gant V. Murray P. (2020). Consolidation of clinical microbiology laboratories and introduction of transformative technologies. Clin Microbiol. Rev. 33 (2), e00057–e00119. 10.1128/CMR.00057-19 32102900 PMC7048017

[B237] Verch T. Roselle C. Shank-Retzlaff M. (2016). Reduction of dilution error in ELISAs using an internal standard. Bioanalysis 8 (14), 1451–1464. 10.4155/bio-2016-0053 27314462

[B238] Verdier M.-C. Tribut O. Tattevin P. Le Tulzo Y. Michelet C. Bentué-Ferrer D. (2011). Simultaneous determination of 12 β-lactam antibiotics in human plasma by high-performance liquid chromatography with UV detection: application to therapeutic drug monitoring. Antimicrob. Agents Chemotherapy 55 (10), 4873–4879. 10.1128/AAC.00533-11 21788467 PMC3187013

[B239] Vogeser M. Seger C. (2008). A decade of HPLC–MS/MS in the routine clinical laboratory—Goals for further developments. Clin. Biochemistry 41 (9), 649–662. 10.1016/j.clinbiochem.2008.02.017 18374660

[B240] Wang K. Zhu Y. Xu F. Liu L. Shi M. Nie J. (2024). Evaluation of omadacycline dosing regimens in Chinese using population pharmacokinetic-pharmacodynamic analysis. Eur. J. Pharm. Sci. Official J. Eur. Fed. Pharm. Sci. 195, 106713. 10.1016/j.ejps.2024.106713 38295963

[B241] Wang P. M. Cornwell M. Prausnitz M. R. (2005). Minimally invasive extraction of dermal interstitial fluid for glucose monitoring using microneedles. Diabetes Technology and Therapeutics 7 (1), 131–141. 10.1089/dia.2005.7.131 15738711

[B242] Wang M. Yang Y. Min J. Song Y. Tu J. Mukasa D. (2022). A wearable electrochemical biosensor for the monitoring of metabolites and nutrients. Nat. Biomed. Eng. 6 (11), 1225–1235. 10.1038/s41551-022-00916-z 35970928 PMC10432133

[B243] Wang Y. Diao Z. Qu Y. Fan K. Feng C. Lv B. (2025). LC-MS/MS quantification of omadacycline in human plasma for therapeutic drug monitoring: method development and clinical application. Sci. Rep. 15 (1), 27728. 10.1038/s41598-025-13396-3 40730882 PMC12307862

[B244] Wicha S. G. Märtson A. G. Nielsen E. I. Koch B. C. Friberg L. E. Alffenaar J. W. (2021). Infectious diseases: Frof therapeutic drug monitoring to model‐informed precision dosing for antibiotics. Clin. Pharmacol. and Ther. 109 (4), 928–941. 10.1002/cpt.2202 33565627

[B245] Williams P. G. Tabah A. Cotta M. O. Sandaradura I. Kanji S. Scheetz M. H. (2023). International survey of antibiotic dosing and monitoring in adult intensive care units. Crit. Care 27 (1), 241. 10.1186/s13054-023-04527-1 37331935 PMC10278304

[B246] Woillard J.-B. Labriffe M. Prémaud A. Marquet P. (2021). Estimation of drug exposure by machine learning based on simulations from published pharmacokinetic models: the example of tacrolimus. Pharmacol. Research 167, 105578. 10.1016/j.phrs.2021.105578 33775863

[B247] Wong S. H. (1989). Advances in liquid chromatography and related methodologies for therapeutic drug monitoring. J. Pharm. Biomed. Analysis 7 (9), 1011–1032. 10.1016/0731-7085(89)80041-x 2490110

[B248] Wu X.-J. Zhang J. Yu J.-C. Cao G.-Y. Shi Y.-G. Zhang Y.-Y. (2012). Establishment of norvancomycin fluorescence polarization immunoassay for therapeutic drug monitoring. J. Antibiotics 65 (1), 35–39. 10.1038/ja.2011.89 22045420

[B249] Xiao W. Huang C. Xu F. Yan J. Bian H. Fu Q. (2018). A simple and compact smartphone-based device for the quantitative readout of colloidal gold lateral flow immunoassay strips. Sensors Actuators B Chem. 266, 63–70. 10.1016/j.snb.2018.03.110 32288251 PMC7127147

[B250] Xiao Y. Xu L. Qian Y. Xu Y. (2024). Identification and characterization of critical values in therapeutic drug monitoring: a retrospective analysis. Sci. Rep. 14 (1), 11520. 10.1038/s41598-024-62402-7 38769456 PMC11106295

[B251] Yadav R. Bulitta J. B. Nation R. L. Landersdorfer C. B. (2017). Optimization of synergistic combination regimens against carbapenem-and aminoglycoside-resistant clinical *Pseudomonas aeruginosa* isolates *via* mechanism-based pharmacokinetic/pharmacodynamic modeling. Antimicrob. Agents Chemotherapy 61 (1), e01011-16. 10.1128/aac.01011-01016 PMC5192108 27821448

[B252] Yaghoubi S. Zekiy A. O. Krutova M. Gholami M. Kouhsari E. Sholeh M. (2022). Tigecycline antibacterial activity, clinical effectiveness, and mechanisms and epidemiology of resistance: narrative review. Eur. J. Clin. Microbiol. and Infect. Dis. 41 (7), 1003–1022. 10.1007/s10096-020-04121-1 33403565 PMC7785128

[B253] Yamada N. Okano T. Sakai H. Karikusa F. Sawasaki Y. Sakurai Y. (1990). Thermo‐responsive polymeric surfaces; control of attachment and detachment of cultured cells. Die Makromol. Chem. Rapid Commun. 11 (11), 571–576. 10.1002/marc.1990.030111109

[B254] Yamato M. Akiyama Y. Kobayashi J. Yang J. Kikuchi A. Okano T. (2007). Temperature-responsive cell culture surfaces for regenerative medicine with cell sheet engineering. Prog. Polym. Sci. 32 (8-9), 1123–1133. 10.1016/j.progpolymsci.2007.06.002

[B255] Yang Y. Song Y. Bo X. Min J. Pak O. S. Zhu L. (2020). A laser-engraved wearable sensor for sensitive detection of uric acid and tyrosine in sweat. Nat. Biotechnology 38 (2), 217–224. 10.1038/s41587-019-0321-x 31768044

[B256] Yang H. Huang Z. Chen Y. Zhu Y. Cao G. Wang J. (2022). Pharmacokinetics, safety and pharmacokinetics/pharmacodynamics analysis of omadacycline in Chinese healthy subjects. Front. Pharmacol. 13, 869237. 10.3389/fphar.2022.869237 35529438 PMC9068897

[B257] Yin T. Liang H. Huang Q. Zhou B. Tang M. Lou J. (2023). A survey of therapeutic drug monitoring status in China. Ther. Drug Monitoring 45 (2), 151–158. 10.1097/ftd.0000000000001060 PMC10013164 36920501

[B258] Yu Z. Liu J. Yu H. Zhou L. Zhao Y. Zhong L. (2023). Should the trough concentration of vancomycin be abandoned in therapeutic drug monitoring? A multicentre, retrospective study of critically ill patients without any form of dialysis. Int. J. Antimicrob. Agents 61 (6), 106812. 10.1016/j.ijantimicag.2023.106812 37037321

[B259] Zheng X. Zhang F. Wang K. Zhang W. Li Y. Sun Y. (2021). Smart biosensors and intelligent devices for salivary biomarker detection. TrAC Trends Anal. Chem. 140, 116281. 10.1016/j.trac.2021.116281

